# Extensive evaluation of the generalized relevance network approach to inferring gene regulatory networks

**DOI:** 10.1093/gigascience/giy118

**Published:** 2018-09-18

**Authors:** Vladimir Kuzmanovski, Ljupčo Todorovski, Sašo Džeroski

**Affiliations:** 1 Department of Knowledge Technologies, Jožef Stefan Institute, Jamova cesta 39, 1000 Ljubljana, Slovenia; 2 Faculty of Public Administration, University of Ljubljana, Gosarjeva ulica 5, 1000 Ljubljana, Slovenia

**Keywords:** network inference, network reconstruction, relevance network approach, gene regulatory networks

## Abstract

**Background:**

The generalized relevance network approach to network inference reconstructs network links based on the strength of associations between data in individual network nodes. It can reconstruct undirected networks, i.e., relevance networks, *sensu stricto*, as well as directed networks, referred to as causal relevance networks. The generalized approach allows the use of an arbitrary measure of pairwise association between nodes, an arbitrary scoring scheme that transforms the associations into weights of the network links, and a method for inferring the directions of the links. While this makes the approach powerful and flexible, it introduces the challenge of finding a combination of components that would perform well on a given inference task.

**Results:**

We address this challenge by performing an extensive empirical analysis of the performance of 114 variants of the generalized relevance network approach on 47 tasks of gene network inference from time-series data and 39 tasks of gene network inference from steady-state data. We compare the different variants in a multi-objective manner, considering their ranking in terms of different performance metrics. The results suggest a set of recommendations that provide guidance for selecting an appropriate variant of the approach in different data settings.

**Conclusions:**

The association measures based on correlation, combined with a particular scoring scheme of asymmetric weighting, lead to optimal performance of the relevance network approach in the general case. In the two special cases of inference tasks involving short time-series data and/or large networks, association measures based on identifying qualitative trends in the time series are more appropriate.

## Introduction

The genome plays a central role in the control of cellular processes in an organism. The sequencing efforts for different organisms have led to the identification of genes, i.e., individual components of the genome. However, the functions of each gene and its product cannot be studied in isolation. To fully understand genome functionality, we have to consider genes and gene products as highly connected and structured networks of information that flow through a cell. These biological networks are typically referred to as gene regulatory networks (GRNs), where nodes correspond to genes or gene products and edges correspond to biological or chemical interactions among them.

Here, we address the task of inference of GRNs from gene expression data. The rapid advance and wide availability of technology for measuring cellular activities at genome-wide scale have caused enormous interest in methods addressing the GRN inference task in contemporary biology. As a result, a wide repertoire of inference methods has been established [1,2,3,4]. In general, methods for GRN inference take one of two major perspectives on the task [5]. One is the statistical perspective, where the focus is on predicting the presence or absence of interactions between genes and gene products. The other, the mathematical modeling perspective, focuses on establishing models that allow for emulating the dynamical activity of the observed natural system.

Methods following the statistical perspective employ a simple "guilt-by-association" heuristic [6,7], a conjecture that similarity of the expression profiles of a set of genes indicates a shared regulation regime among them. Initially, this conjecture led to methods for inferring undirected, co-expression, and association networks [3], commonly referred to as relevance networks [8]. Later, these methods were first enriched with statistical techniques for estimating conditional independence to distinguish between direct and indirect interactions [9]. Later still, techniques for inferring the direction of a given network interaction were added to the overall inference methods employed, leading to methods for inferring causal, directed networks [10].

Taken together, these developments have led to a generalized relevance network approach [10] that follows the statistical perspective of GRN inference and predicts network links based on the pairwise associations between node expression levels. The degree of a pairwise association is evaluated using a measure of similarity (or distance) between the expression levels of the two corresponding genes. Furthermore, the generalized approach includes scoring methods for refining the original similarity scores toward link weights that distinguish between direct and indirect influences. Finally, the extended approach includes methods for inferring the link directions from data. To make a clear distinction between the original relevance networks and their generalized instances, introduced by [10], we refer to the latter as causal relevance networks (CRNs).

Data for GRN inference come typically from microarray experiments perturbing and stressing genes that produce highly resolved time-series and steady-state measurements of transcript levels. Steady-state measurements are made by perturbing every gene in the network and recording the pseudo state reached after the perturbation. Perturbing every gene is not necessary for time-series data that record gene expression levels over a certain period of time after the perturbation. For both data types, the captured dynamic response of the regulatory effects within a cell should provide robust information about the GRN under consideration [11]. While the original relevance network approach [8] and its extensions toward causal networks [9] have been dealing with steady-state data, the generalized relevance network approach has been proven capable of also handling time-series data [10].

The variety of similarity measures and scoring schemes that can be applied within the generalized relevance network approach makes the approach flexible and applicable in various scenarios. Many surveys emphasize and focus on the flexibility of the relevance network approach by presenting and categorizing its variants. The distinctive aspect of the survey presented here is the focus on selecting an appropriate variant of the relevance network approach for a given dataset. Our basic conjecture is that some variants of the approach perform better than others, in general, and that the performance of the variant is related to the properties of the dataset at hand. Another contribution of our survey is that it includes inference tasks from the two types of data, i.e., steady-state and time-series data.

To test the validity of our conjecture, we perform an extensive comparative analysis of the performance of 114 variants of the relevance network approach on 86 tasks of inferring GRN from data, 47 from time-series data, and 39 from steady-state data. The tasks include inference from real microarray measurements of the microorganisms *Escherichia coli* and *Saccharomyces cerevisiae* (*Yeast*) [12–14], as well as of their simulation counterparts (networks) [10,15]. Additionally, simulated data from *in silico* networks from [12] and [13,16,17] have been included in the study.

Our conjecture is analyzed through three dimensions: the impact of the type of data (steady-state vs time-series), time-series length, and network size on the performance of the different variants of the relevance network approach. The performance of network inference is measured using three different performance metrics widely used for assessment of inferred networks by comparing them to the gold standard, i.e., the set of known "true” interactions among the network nodes.

The study is organized as follows. First, we introduce the generalized relevance network approach and review its variants that stem from different measures of association between network nodes. The survey of the variants of the relevance network approach expose their theoretical advantages and disadvantages, as well as the history of their applications for inference of gene regulatory networks. Section *Materials & Methods* introduces the experimental setup of the comparative analysis in terms of the GRN tasks addressed, datasets employed, and metrics used to measure the performance of the network inference methods. Following section presents and discusses the results, with an emphasis on what they tell as about the most appropriate variant of the relevance network approach for a given GRN inference task. Finally, the last section provides a brief summary of the comparative analysis and an outline of the directions for further research.

## The generalized (causal) relevance network approach

The generalized relevance network approach infers network structure by measuring the pairwise associations between the data observed in the individual network nodes. It follows the more general statistical perspective of GRN inference, where no explicit model of the data is built or assumed. The retrieved knowledge about the pairwise association between nodes is interpreted as the relevance of the individual network links [1,18].

The relevance network approach has been introduced by [8], where pairwise association between gene expression profiles is measured by using mutual information. Different measures of association based on Euclidean distance and correlation coefficients have been previously used for identifying co-expression of genes [19] or association GRNs [19,20]. In more recent studies, the generalized network approach has been extended to include other association measures and additional steps for interpreting the measured associations, such as the symmetry-breaking methods for identifying directions of links or methods for marginal control of the association [10,13,21]. Since they infer link directions and remove spurious links, we can interpret the inferred networks as causal networks, hence the reference causal relevance networks.

Hempel et al. [10] decomposes the CRN approach into three components of (1) inference of pairwise associations, (2) marginal control of association, and (3) breaking symmetry. The first step employs a distance measure, a correlation coefficient, or a mutual-information measure to assess the association between two network nodes. The result of the first step is a symmetric matrix, the elements of which indicate the strength of the undirected network links. The second step of the marginal control of the association scores includes various scoring schemes, which transform the association-scores matrix into a symmetric or an asymmetric matrix of weights of the network links. The last component, as its name indicates, breaks the symmetry of the symmetric network weights matrices, typically by using a time-shifting technique. Note that the latter can only be used on time-series data.

Overall, the main idea behind the CRN approach is to assign a higher relevance to a hypothesized network link, which is identified with a strong pairwise association between the corresponding nodes. Thus, the relevance score provides an opportunity for differentiating the possibilities of existence of individual network links. Finally, to obtain the inferred network structure, one has to decide upon a threshold value used to map the numeric relevance score into a discrete binary value to indicate the validity of the initial hypothesis, i.e., the presence of a network link.

The remainder of the section follows the decomposition of the CRN approach into the three components introduced above. First, we introduce all the measures used to estimate pairwise association between nodes in our comparative study of the CRN-approach variants. Next, we introduce the scoring schemes for marginal control of association. Finally, we introduce the time-shifting method for breaking the symmetry of the association/weight matrices.

### Association measures

The association measures used in this study can be broadly categorized into three clusters of correlation-based, information-based, and distance-based measures. Correlation-based measures treat the expression profiles as data samples and calculate a correlation coefficient between them. Information-based measures treat the expression profiles as random variables and calculate their nonlinear dependence using mutual information. Distance-based measures calculate the association as an inverse of the distance between the observed profiles and can be further clustered in three subgroups. Simple distance measures in the first group treat profiles as vectors. The second group includes a single distance measure of dynamic time wrapping that operates on time series directly. The symbolic measures in the third group operate on symbolic (or qualitative) representations of time-series trends.

In each of the following subsections, we present one of the groups of association measures introduced above. Throughout this section, we use the Greek lower-case letter μ to denote pairwise associations between gene expression profiles and δ for distances between them.

#### Correlation-based measures

Correlation-based measures consider the expression profiles *x* = 〈*x*_1_, *x*_2_, …*x*_*n*_〉 and *y* = 〈*y*_1_, *y*_2_, …*y*_*n*_〉 as population samples. This allows for the use of an arbitrary correlation coefficient over these samples. In particular, we use three of them in this study: the Pearson, Spearman, and Kendall rank correlation coefficients.

The *Pearson correlation coefficient* quantifies the linear relationship between the samples *x* and *y* as

\begin{equation*}
{\mu} _P(x,y) = \frac{\sum _{k=1}^{n}(x_k - \bar{x})(y_k - \bar{y})}{\sqrt{\sum _{k=1}^{n}(x_k - \bar{x})^2} \cdot \sqrt{\sum _{k=1}^{n}(y_k - \bar{y})^2}},
\end{equation*}

where $\bar{x}$ and $\bar{y}$ denote the sample means of *x* and *y*, respectively.

As mentioned before, the Pearson correlation coefficient was first employed for identifying clusters of co-expressed genes by [19]. Later, it has been regularly used as a state-of-the-art association measure integrated and compared with other methods [1,10,22].

The *Spearman rank correlation coefficient* is based on the rank distribution of the observed expression values. It can be used as a more general measure of interdependencies that is not restricted to linear relationships and defines the interdependency between *x* and *y* as:

\begin{equation*}
\mu _S(x,y) = \mu _P(R(x), R(y))
\end{equation*}

where *R*(*u*) = 〈*r*(*u*_1_), *r*(*u*_2_), …*r*(*u*_*n*_)〉 and *r*(*u*_*k*_) denotes the rank of *u* in *u*_*k*_, respectively. The Spearman rank correlation is often used as an association measure in variants of the CRN approach [1,23,24].

The *Kendall rank correlation coefficient* is a measure of correlation between ranks of two samples *x* and *y* defined as [25]:

\begin{equation*}
\mu _K(x,y) = \frac{2(n_c - n_d)}{n(n-1)},
\end{equation*}

where *n*_*c*_ is the number of concordant pairs of points in *x* and *y*, while *n*_*d*_ is the number of discordant pairs. A concordant pair of time points *i* and *j* is concordant if both *x*_*i*_ > *x*_*j*_ and *y*_*i*_ > *y*_*j*_ or both *x*_*i*_ < *x*_*j*_ and *y*_*i*_ < *y*_*j*_. Otherwise, the pair is discordant. The Kendall rank correlation is rarely used as an association measure in the variants of the CRN approach, with a few notable exceptions in recent studies [10].

Correlation-based measures applied for inferring associations among genes in a GRN have been widely used in the domain of network inference. The rank correlation coefficient does not necessarily consider a continuous scale of expression vectors; it considers discrete ranks instead. Note, furthermore, that correlation-based measures dismiss the time component of the time-series data. Finally, note that the resulting pairwise association matrices are symmetric and cannot be used to infer the direction of the network links.

#### Information-based measures

Information-based or information-theoretic measures calculate the association between expression profiles *x* = 〈*x*_1_, *x*_2_, ...*x*_*n*_〉 by considering them to be random variables. The most commonly used metric of this group is simple mutual information [26]. In our study, we use it in a combination with different statistical estimators of entropy and discretization methods, introduced below.

*Mutual information* quantifies the possibly nonlinear interdependencies between two random variables *X* and *Y*. It can be computed by using different entropy estimators but usually fails to discover indirect links, representing them as direct links instead, between the nodes in the reconstructed network [27]. The general form of mutual information (MI) relates the marginal entropies of *X* and *Y*, *H*(*X*) and *H*(*Y*), and their joint entropy *H*(*X*, *Y*):

\begin{equation*}
\mu _I(x,y) = H(X) + H(Y) - H(X,Y).
\end{equation*}

Three estimators of entropy of a given random variable are being widely used for GRN inference: the maximum likelihood estimator, the Miller-Madow estimator, and the shrink entropy estimator.

The *maximum likelihood estimator* assesses the entropy of a given empirical distribution of a random variable *X* following the Shannon entropy definition [28]:

\begin{equation*}
H^{emp}(X) = -\sum _{k=1}^{n} p(x_k) \cdot log(p(x_k)).
\end{equation*}

This estimator is highly dependent on the number of bins *n* and the length of observations, which can increase the bias, while the estimator variance is kept minimal.

The *Miller-Madow estimator* is based on maximum likelihood estimator but corrected with a second additive term representing the asymptotic bias:

\begin{equation*}
H^{mm}(X) = H^{emp}(X) + \frac{|X|-1}{2n},
\end{equation*}

where |*X*| is the number of bins with non-zero probability. The Miller-Madow estimator is preferred over the maximum likelihood estimator due to the reduction of the bias without increasing the variance or the computational cost [28].

The *shrink entropy estimator* [29] regularizes the maximum likelihood estimator. The idea is to combine two different estimators, one with low variance and another one with low bias, by using a shrinking factor λ ∈ [0, 1]:

\begin{equation*}
H^{shrink}(X) = -\sum _{k=1}^{n} p_{\lambda }(x_k) \cdot log(p_{\lambda }(x_k)),
\end{equation*}

where *p*_λ_ is defined as follows:

\begin{equation*}
p_{\lambda }(x_k) = \lambda \frac{1}{|X|} + (1-\lambda )p(x_k).
\end{equation*}

If the value of λ is close to zero, the estimated entropy is close to the value of the basic maximum likelihood estimator. Otherwise, if it is close to 1, the entropy estimation tends to be closer to the bias term.

The statistical estimators are combined with two different methods for discretization of numeric random variables: equal width and equal frequency. All six possible combinations of estimators and discretization methods are used in the comparative analysis of the CRN-approach variants.

*Equal width* is a fixed bin-width discretization method that discretized the values of the numeric variable into equally sized bins, i.e., ranges of variable values. The *equal frequencies* discretization method partitions the range of the given random variable *X* into ranges of an equal number of data points. Thus, it results in bins with different sizes [30]. In both cases, the default number of bins equals the squared root of the number of observations of the variable [31].

The literature overview reveals that the CRN approach often uses mutual information as a measure of association [1,10,13,22,23,27]. In that context, [1] consider mutual information as a baseline relevance network approach variant. Mutual information is capable of discovering nonlinear interdependencies but, as with other simple association measures, is not able to infer the direction of influences and produces undirected networks.

#### Simple distance measures

Simple distance measures assess the strength of gene regulatory interactions by calculating the distance between gene expression profiles. In general, they operate over vectors of values and share the same approach or ground norm. Three different measures have been included in the study: the *L*^10^ Norm (Minkowsky), Euclidean, and Manhattan distance.

The ground norm that appears as a basis for all three distance measures is the so-called *L*^*s*^ Norm or Minkowsky distance:

\begin{equation*}
\delta _L(x,y) = (\sum _{k=1}^{n} |x_k - y_k|^s)^{(1/s)},
\end{equation*}

where *s* represents the dimension of the space in which vectors *x* and *y* are compared [32]. We consider three distance measures corresponding to the three norms of *L*^10^ Norm (*s* = 10), Euclidean distance (*s* = 2), and Manhattan distance (*s* = 1).

The three distance measures share a common limitation of detecting linear interdependencies between expression profiles. The determination of gene regulatory interactions is based on raw vectors, the time component of which is dismissed. They have been applied in the context of relevance networks from the early stage of development of this approach [20] and since then are being regularly used and surveyed [1,3,10,13].

#### Dynamic time warping

Dynamic time warping (DTW) relies on finding an optimal distance mapping between two time series. It tries to capture differences between time series with regard to time and speed of change. Originally developed in the context of speech recognition [33], it has found its use in a wide range of applications in the domains of medicine and bioinformatics [34,35].

The algorithm for calculating the DTW measure proceeds in two steps. First, local distances are calculated for all pairs of points from the two time series using the simple Euclidean distance. Then, the pairs of time points are aligned so that a minimal path is found, where each point is included at least once and the sum of all the distances is minimized. DTW allows specifying constraints of the alignment paths; here we use three of them, *symmetric1*, *symmetric2*, and *asymmetric*. The descriptions of the constraints can be found in the documentation of the ”*dtw*” R-package [36] used in our experiments.

#### Symbolic measures

The simple qualitative distance is based on a qualitative comparison of the shape or trends of time series. In essence, the simple qualitative distance observes the qualitative trend of change of the time-series values between each pair of time points. It compares the observed qualitative trends in the time-series *x* against the ones observed in *y*:

\begin{equation*}
\delta _{QD}(x,y) = \sum _{k=1}^{n-1} \sum _{j=k+1}^{n} \frac{2 \cdot {\it Diff}(q(x_k,x_j),q(y_k,y_j))}{n \cdot (n-1)},
\end{equation*}

with *Diff*(*q*_1_, *q*_2_) a function that defines the difference between different qualitative changes, which are defined as *increase* if *x*_*k*_ < *x*_*j*_, *no-change* if *x*_*k*_ ≈ *x*_*j*_, and *decrease* if *x*_*k*_ > *x*_*j*_.

The simple qualitative distance has been proposed by [37] and has been used in the context of clustering gene expression time series by [38]. The simple qualitative distance can be computed for very short time series, without decreasing the quality of the estimate of the association between them. Furthermore, it captures the nonlinear interdependencies between gene expressions.

Symbolic similarity measures operate on symbolic dynamics in order to uncover patterns of interaction. These similarity measures have been applied in the domain of bioinformatics by [39]. Later, [10] report upon exhaustive research and application of symbolic similarity measures in the domain of relevance networks.

Symbolic similarity measures transform the observed time series into sequences of symbols [40]. The complete guidance of performing this step is also presented in the work by [10]. In this study, we include three symbolic similarity measures of symbol sequence similarity, mutual information over symbol vectors, and linear combination of both.

An important disadvantage of these measures is the computation time if the time series are longer. Hence, possible constraints are applicable with regard to the length of symbol sequences α, which in our case has been determining as follows:

\begin{equation*}
\alpha = \left\lbrace \begin{array}{@{}l@{\quad }l@{}}[1/n], & \text{if}\ n\lt 10 \\
5, & \text{otherwise} \end{array}\right.
\end{equation*}

where *n* denotes the time-series length.

### Scoring schemes

Scoring schemes are considered in order to control the resulting association scores, henceforth *weights*. There are various scoring schemes that can go along with the above-mentioned association measures, but here we limit the set to the ones listed by [10]: reconstruction of accurate cellular networks, context likelihood of relatedness, maximum relevance/minimum redundancy network, and asymmetric weighting.

Accurate Reconstruction of Accurate Cellular NEtworks (ARACNE) is based on the data processing inequality [41] paradigm and states that post-processing cannot improve the already acquired knowledge. In essence, it tests all gene triplets *i*, *j*, and *k*, where all three pairs have mutual information greater than some threshold *I*_0_. For each such triplet, the edge corresponding to the lowest mutual information *I*_1_ is eliminated from the adjacency matrix:

(1) \begin{equation*}
A_{i^{\prime }j^{\prime }} = A_{j^{\prime }i^{\prime }} = \left\lbrace \begin{array}{@{}l@{\quad }l@{}}0, & \text{if}\ I_{i^{\prime } j^{\prime }} \ge I_2(1-\epsilon ) \\
1, & \text{otherwise} \end{array}\right. 
\end{equation*}

where $I_{i^{\prime }j^{\prime }}=argmin\lbrace I_{ij},I_{jk},I_{ik}\rbrace$ is the lowest mutual information of the three, *I*_2_ is the second lowest mutual information, and factor ε is a tolerance parameter with a value between 0 and 1 [9,42]. Moreover, ARACNE removes all edges satisfying $I_{i^{\prime }j^{\prime }} \lt \tau$, where τ is predefined threshold [10].

ARACNE is capable of controlling the regulation of a gene over another gene by modifying the initially inferred network on the basis of mutual information. However, the resulting network is still undirected.

Context Likelihood of Relatedness (CLR) [43] is an extension to the basic relevance network approach proposed by [8]. Unlike ARACNE, CLR performs pairwise comparison of mutual information values. In the second step, it estimates the statistical likelihood of a mutual information value for a given pair of genes (*I*_*kj*_) by comparing it to the marginal (gene-specific) distribution. Thus, two scores are derived, one for gene *k* and one for gene *j*. By making the normality assumption about these distributions, the corresponding scores *z*_*k*_ and *z*_*j*_ are calculated as follows:

(2) \begin{equation*}
z_k = max(0,\frac{1}{\sigma _k}-\frac{\bar{I_{k}}}{I_{kj} \cdot \sigma _k}), 
\end{equation*}

(3) \begin{equation*}
z_j = max(0,\frac{1}{\sigma _j}-\frac{\bar{I_{j}}}{I_{kj} \cdot \sigma _j}). 
\end{equation*}

The final score for a pair of genes is obtained as follows:

(4) \begin{equation*}
z_{kj}=\sqrt{z_k^2 + z_j^2}. 
\end{equation*}

In contrast to ARACNE, CLR does not rely on a global threshold but on local background values computed for of each gene separately. The outcome of CLR is an undirected network.

Maximum Relevance/minimum redundancy NETwork (MRNET) is a supervised method that performs a series of maximum relevance/minimum redundancy gene selection procedures [44]. The expression of a given gene is considered as a target *y* = *x*_*k*_ and the rest of genes from *V* = *x*∖*x*_*k*_ as descriptive variables in the supervised procedure. Given the set *M* of selected variables and pairwise weights *w*_*kj*_, the procedure updates *M* by choosing the variable:

(5) \begin{equation*}
x_j^{MRMR} = argmax(s_j), x_j \in V\setminus M, 
\end{equation*}

that maximizes the score:

(6) \begin{equation*}
s_j = u_j - r_j, 
\end{equation*}

where $r_j=\frac{1}{|M|}\sum _{x_i \in M} w_{ji}$ is the redundancy term and *u*_*j*_ = *w*_*jk*_ is the relevance term.

The above procedure tries to differentiate between direct and indirect links. Direct links are assigned higher importance (relevance) and indirect links lower importance (higher redundancy). Thus, the entries in the final matrix *f*_*kj*_ are calculated as:

(7) \begin{equation*}
f_{kj} = \frac{max[(w_{jk} - r_j),(w_{kj} - r_k)]}{w_{kj}}. 
\end{equation*}

MRNET assigns weights *w* based on simple mutual information and employs an additional parameter τ that is used for eliminating edges with an unimportant score. The algorithm is not capable of inferring directionality in the GRN.

Asymmetric WEighting (AWE) is an asymmetric weighting schema based on the topological aspects of a complete set of pairwise weights obtained from a particular association method [10]. Given a matrix, AWE assumes its columns are genes that are regulated by other genes and its rows are genes that regulate other genes. The asymmetric weights *c*_*kj*_ are then calculated by dividing each entry by the sum of the corresponding column scores:

(8) \begin{equation*}
c_{kj} = w_{kj} \cdot f_{j}, 
\end{equation*}

(9) \begin{equation*}
f_{j} = (\sum _{k=1}^{m} w_{kj})^{-1}. 
\end{equation*}

where *f*_*j*_ corresponds to the amount of regulation received by gene *j* and *c*_*kj*_ to the probability that gene *j* is regulated by gene *k*. The probabilities that the *j*^*th*^ gene is regulated by each of the other genes sum up to unity:

(10) \begin{equation*}
\sum _{k=1}^{m}c_{kj} = \sum _{k=1}^{m} w_{kj} \cdot f_{j} = 1, 
\end{equation*}

This scoring schema is capable of inducing directionality in a GRN. The first application of the schema in the domain of generalized relevance networks is given in [10].

### Time shifting

Time shifting is a method for inferring the direction of an undirected link between two network nodes from time-series data. The main idea is to shift one of the time series, e.g., *X* in one direction, and observe the change of the association μ(*X*, *Y*) using a particular association measure μ. The change of the association measure with the time shift provides information that can be used to infer the direction of the influence, i.e., the direction of the network link. The complete procedure is described by [10] and [45].

We use the time-shifting method as a third, obligatory component of the CRN approach in all cases where the scoring scheme results in undirected network. For the AWE scoring scheme, where the result is a directed network, the use of the time-shifting method is optional. In that case, we have two alternative CRN-approach variants: one with and one without applying time shifting. The time-shifting method is not applicable in case of steady-state data.

## Materials and Methods

In the comparative evaluation of the variants of the CRN approach, we have considered all combinations of association measures and scoring schemes, with time shifting applied where appropriate. There are 114 candidate combinations corresponding to 114 variants of the relevance network approach. Each of the 114 variants was applied to the 47 tasks of GRN inference from time-series data and 39 tasks of GRN inference from steady-state data. Performance was measured by comparing the inferred network structure with the structure of the given network (in case of reconstructing known networks from simulated data) or with the structure of the best known network (in case of real measurements). We use performance measures, one of which is the area under the receiver-operator characteristics curve, and the other two are different versions of the area under the precision-recall curve.

The goal of the comparative analysis is to identify the best-performing variants of the CRN approach and the properties thereof. We are especially interested in finding out what association measures and scoring schemes work best and what are the interactions between them that lead to the best performance. We also investigate the impact of the time-series length and network size on the best-performing variants of the CRN approach.

To identify the best-performing methods for a given set of GRN inference tasks, we proceed as follows. First, for each performance measure and each task, we sort and rank the methods in decreasing order with respect to their performance on the task, so the top-performing method gets the rank of 1 and the worst-performing method the rank of 114. Furthermore, for each performance measure, for each method we calculate the average ranks of the method for the given set of tasks. Finally, we perform a Pareto analysis of the three-dimensional space of performance metrics to identify Pareto fronts of points corresponding to the best-performing methods, i.e., methods with the lowest average ranks.

The remainder of this section provides further details on the experimental setup for performing the comparative analysis. We first introduce the tasks of GRN inference, then provide a detailed description of the performance metrics used and conclude with a brief overview of the implementation details.

### Data description

The comparative study has been conducted using real and simulated micro-array data over time-course and steady-state conditions. In particular, the data or the simulation model used for obtaining data are based on *in silico* networks and real networks of two microorganisms: *Escherichia coli* and *Saccharomyces cerevisiae* (Yeast). We use datasets from five previously published studies on GRN inference and related benchmarks.

The first data source is [10], where datasets were generated by the tool SynTReN [46] on the basis of the well-known gene regulatory networks in *E.coli* and Yeast. We consider subnetworks of 100, 150, and 200 genes, characterized by 121, 202, and 303 existing links with an average node degree of 2.42, 2.46, and 3.03, respectively. In order to guarantee consistency between subnetworks and expression data, SynTReN generates different expression data for each selected subnetwork. Additionally, three level of noise have been considered: 0.0 (deterministic - without noise), 0.1, and 0.5. These values represent the σ parameter of the log-normal distribution ∼*logX*(0, σ), according to which the noise is generated by SynTReN. For each configuration, 6 technical replicates of 10 time points have been generated and the expression data associated with each gene obtained as the average over the replicates. This is necessary to cope with the nondeterministic nature of the SynTReN data generation algorithm. This source has been employed for time-series analysis only (the 18 dataset labels starting with *E1* and *Y1* in Table 1).

**Table 1.** Properties (columns) of the time-series datasets for 47 GRN inference tasks (rows): organism, dataset label, percentage of network nodes covered in the dataset, number of network nodes, and time-series length

Organism	Dataset	Coverage	Size	Length	Noise
E. coli	*E1_1*	100	100	10	0.0
E. coli	*E1_2*	100	150	10	0.0
E. coli	*E1_3*	100	200	10	0.0
E. coli	*E1_4*	100	100	10	0.1
E. coli	*E1_5*	100	150	10	0.1
E. coli	*E1_6*	100	200	10	0.1
E. coli	*E1_7*	100	100	10	0.5
E. coli	*E1_8*	100	150	10	0.5
E. coli	*E1_9*	100	200	10	0.5
E. coli	*E2_1*	100	4,511	6	0
E. coli	*E2_2*	100	4,511	5	0
Yeast	*Y1_1*	100	100	10	0.0
Yeast	*Y1_2*	100	150	10	0.0
Yeast	*Y1_3*	100	200	10	0.0
Yeast	*Y1_4*	100	100	10	0.1
Yeast	*Y1_5*	100	150	10	0.1
Yeast	*Y1_6*	100	200	10	0.1
Yeast	*Y1_7*	100	100	10	0.5
Yeast	*Y1_8*	100	150	10	0.5
Yeast	*Y1_9*	100	200	10	0.5
Yeast	*Y2_1*	100	5,950	5	0
Yeast	*Y2_2*	100	5,950	48	0
Yeast	*Y3_1_2*	100	42	5	0
Yeast	*Y3_1_3*	100	42	5	0
Yeast	*Y3_1_11*	97.6	41	8	0
Yeast	*Y3_1_13*	100	42	5	0
Yeast	*Y3_1_14*	100	42	5	0
Yeast	*Y3_1_15*	100	42	10	0
Yeast	*Y3_2_2*	96	72	5	0
Yeast	*Y3_2_14*	96	72	5	0
Yeast	*Y3_3_2*	96.3	289	5	0
Yeast	*Y3_3_3*	95.7	287	5	0
Yeast	*Y3_3_10*	95.3	286	7	0
Yeast	*Y3_3_13*	96.7	290	5	0
Yeast	*Y3_3_14*	95.7	287	5	0
Yeast	*Y3_4_2*	96.3	181	5	0
Yeast	*Y3_4_3*	95.7	180	5	0
Yeast	*Y3_4_13*	95.2	179	5	0
Yeast	*YI_SON_1*	100	5	10	0
Yeast	*YI_SON_2*	100	5	15	0
Yeast	*YI_SON_3*	100	5	9	0
Yeast	*YI_SON_4*	100	5	9	0
Yeast	*YI_SOFF_1*	100	5	15	0
Yeast	*YI_SOFF_2*	100	5	18	0
Yeast	*YI_SOFF_3*	100	5	18	0
Yeast	*YI_SOFF_4*	100	5	20	0
Yeast	*YI_SOFF_5*	100	5	20	0

Another source of data are the *DREAM4* [16,17] and the *DREAM5 challenges* [12,13]. The former is considered in the analysis of steady-state data, where 10 different *in silico* networks (five of size 10 and five of size 100 genes) have been perturbed with three different approaches, producing, in total, 30 datasets (in Table 2 given with labels starting with *IS2*).

**Table 2.** Properties (columns) of the steady-state datasets for 39 GRN inference tasks (rows): organism, dataset label, percentage of network nodes covered in the dataset, number of network nodes, and number of records (observations)

Organism	Data set	Coverage	Size	Records
E. coli	*E2_3*	100	4,511	342
Yeast	*Y2_3*	100	5,950	238
Yeast	*YI_GLU_1*	100	5	6
Yeast	*YI_GLU_2*	100	5	6
Yeast	*YI_GLU_3*	100	5	6
Yeast	*YI_GAL_1*	100	5	6
Yeast	*YI_GAL_2*	100	5	6
Yeast	*YI_GAL_3*	100	5	6
In silico	*IS1_1*	100	1,643	342
In silico	*IS2_1_1*	100	10	10
In silico	*IS2_1_2*	100	10	10
In silico	*IS2_1_3*	100	10	10
In silico	*IS2_2_1*	100	10	10
In silico	*IS2_2_2*	100	10	10
In silico	*IS2_2_3*	100	10	10
In silico	*IS2_3_1*	100	10	10
In silico	*IS2_3_2*	100	10	10
In silico	*IS2_3_3*	100	10	10
In silico	*IS2_4_1*	100	10	10
In silico	*IS2_4_2*	100	10	10
In silico	*IS2_4_3*	100	10	10
In silico	*IS2_5_1*	100	10	10
In silico	*IS2_5_2*	100	10	10
In silico	*IS2_5_3*	100	10	10
In silico	*IS2_6_1*	100	100	100
In silico	*IS2_6_2*	100	100	100
In silico	*IS2_7_1*	100	100	100
In silico	*IS2_7_2*	100	100	100
In silico	*IS2_8_1*	100	100	100
In silico	*IS2_8_2*	100	100	100
In silico	*IS2_9_1*	100	100	100
In silico	*IS2_9_2*	100	100	100
In silico	*IS2_10_1*	100	100	100
In silico	*IS2_10_2*	100	100	100
In silico	*IS2_11_1*	100	100	100
In silico	*IS2_12_1*	100	100	100
In silico	*IS2_13_1*	100	100	100
In silico	*IS2_14_1*	100	100	100
In silico	*IS2_15_1*	100	100	100

The latter (*DREAM5 challenge*) is considered in the analysis of both time-series and steady-state data. Originally, the challenge provides five networks, of which we consider three: Network1, Network3, and Network4, based on Affymetrix gene expression data of *In silico*, *E.coli*, and *Yeast* networks, respectively, taken from the Gene Expression Omnibus database [47] and collected under a wide range of biological conditions. For each network, a set of experiments has been performed over its genes.

For the case of time-series analysis, we consider Network3 and Network4 with two experiments per network and create four tasks (datasets). Network3 contains 4,511 genes and 2,066 known (existing) links with density of 1.1^−3^, while Network4 has 5,950 genes, 3,940 known links, and density of 3.8^−4^. Time-series lengths vary from 5 to 48 time points. The four rows in Table 1 with dataset labels containing *E2* and *Y2* provide summary description of the four tasks corresponding to the DREAM5 data source for time-series data.

For steady-state data, all three networks are considered, each with one dataset. Network1 contains 1,643 genes and 3,940 interactions (links). Steady-state datasets from Network 1 and Network 3 contain 342 records (observations), while the dataset from Network4 has 238 records. Complete references are given in Table 2, marked with labels: *IS1_1*, *E2_3*, and *Y2_3*, respectively, for Network1, Network3, and Network4.

The fourth data source provides real measurements, collected as a part of the study conducted by [14], which aims to explore changes in expression levels of Yeast genes under diverse environmental stresses, such as heat shock, diauxic shift, diamide treatment, and amino acid starvation. The measurements have been taken at different time points, using microarrays. One network has been observed, where four different independent subnetworks were identified, which are considered as separate networks within this study, with node sizes of 42, 75, 300, and 300. For the observed subnetworks, 13 different stresses have been monitored, thus 52 datasets are available. Since some datasets provide limited coverage of the network nodes, we consider only 20 datasets that have network coverage greater than 95%. The time series observed are of different lengths, from 5 to 11 time points (the last 16 rows in Table 1).

The last data source is a benchmark study that proposes a synthetic network for *in vivo* benchmarking, based on a Yeast gene network [15]. The network is composed of five genes, where the genes regulate each other through a variety of interactions. Microarrays have been measured over time-course and steady-state conditions upon multiple perturbations. Table 1 shows the properties of the time-series datasets, from this source labeled with YI at the beginning. Similarly, Table 2 presents the properties of the steady-state datasets.

### Performance metrics

To evaluate the performance of the inference method on a given task, we perform a matching between the structure (links) of the given/known GRN (true network) and the structure (links) of the inferred GRN (inferred network). Since the output of the inference method is a network connectivity matrix containing numeric link weights, we can perform the matching after setting the threshold value that would decide upon the presence and absence of links. To this end, we set aside metrics that require prior assumptions, i.e., performance metrics that require a predefined or default discrimination threshold. Instead, we follow the standard framework for evaluating network inference and employ *t*hresholding metrics, which consider the variability of the discrimination threshold and avoid setting it to a default value. Thus, methods are evaluated with regard to the complete set of possible thresholds, which results in an analysis of the performance space. For this purpose, two different spaces have been applied: receiver operating characteristic (ROC) curve and precision-recall (PR) curve space.

Both spaces are defined over quantities derived from a confusion matrix. A confusion matrix [48,49] is a matrix that consists of four basic numbers that represent the correctness of link predictions: number of correctly recognized true network links (true positives [TP]), number of correctly recognized absent links in the true network (true negatives [TN]), and links that either have been incorrectly predicted to be present (false positives [FP]) or true network links that were predicted as absent (false negatives [FN]). These basic numbers are further combined in order to express more specific performance perspectives. In the following formulas, we use *P* to denote the number of true network links and *N* to denote the number of absent links in the true network.

**ROC curve**. This is a two-dimensional space that illustrates the performance of a binary classifier as its discrimination threshold is varied [50]. Its dimensions correspond to the two performance metrics of the TP rate (TPR) (Eq. 11) and false positive rate (FPR) (Eq. 12), for various threshold settings. It depicts the relative trade-offs between TPs and FPs, which are interpreted as benefit and cost, respectively.

(11) \begin{equation*}
\text{TPR} = \frac{\text{TP}}{\text{P}} = \frac{\text{TP}}{\text{TP} + \text{FN}} 
\end{equation*}

(12) \begin{equation*}
\text{FPR} = \frac{\text{FP}}{\text{N}} = \frac{\text{FP}}{\text{FP} + \text{TN}} 
\end{equation*}

Since the ROC curve is two dimensional, various summary statistics can be derived from it. Most commonly used is the area under the curve (AUC) that quantifies the area that is found below the curve, which is also considered in our study for the comparative evaluation. The AUC is calculated by integrating the AUC and expressing it as a single quantity (area in two-dimensional space).

The ROC space is a unit two-dimensional space with a total area of 1. Thus, it can be plotted on a two-dimensional plot with both axes ranging from 0 to 1. Furthermore, the ROC curve is monotonic, which to a certain extent guarantees that by considering the curve, an optimal threshold can be found. The ROC curve or analysis overall is suitable for comparison of a classifier with a default classifier (random selection), which is represented in the space as a diagonal line from (0,0) to (1,1).

However, ROC analysis has disadvantages, as well. Mainly, it can be misinterpreted if the problem under consideration is characterized with an imbalanced distribution of class values. This disadvantage can appear due to the fact that true negatives are considered as correct classifications of examples, even though the problem focuses on the correct classification of positive examples (classification of minority class) only. Reconstruction of GRNs is such a problem where we face networks with many nodes, but a very small number of existing links (minority class), and many nonexisting links (majority class). Hence, correct classification of the former is a much more complex task than the correct classification of the latter. The ROC analysis dismisses the complexity of the classification tasks and considers the correct classification of the minority class of existing links to be equally important as the correct classification of nonexisting links.

The AUC quantity also has its own properties. Its values range from 0 to 1; values close to 1 represent better classifiers, while values around 0.5 mean that the classifier is no better than the default (random) classifier. Values below 0.5 mean that the evaluated classifier behaves worse than the default classifier. The disadvantages of the ROC curve are reflected also in the AUC quantity. Namely, considering the problem of GRN reconstruction, we can end up with overall high AUC, increased mainly by the accurate classification of the majority class (correctly predicting nonexisting links).

The PR curve is also a two-dimensional space that defines the performance of a binary classifier as its discrimination threshold is varying [51,52]. Commonly, it is used as a replacement for the ROC curve in the case of highly imbalanced class distribution [53]. The space is defined with two metrics derived from the confusion matrix: recall (Eq. 13) and precision (Eq. 14).

(13) \begin{equation*}
\text{recall} = \text{TPR} = \frac{\text{TP}}{\text{P}} = \frac{\text{TP}}{\text{TP} + \text{FN}} 
\end{equation*}

(14) \begin{equation*}
\text{precision} = \frac{\text{TP}}{\text{TP} + \text{FP}} 
\end{equation*}

Summary statistics can also be derived from the PR curve, such as the commonly used area under the PR curve (AUPRC). In this analysis, we used two different portions of the area under the curve: partial *AUPRC-0.2* and total area *AUPRC*. Guided by the importance of discovering only true links, without an expectation that all of them would be discovered, we consider the 20% of the AUPRC that corresponds to lower recall (up to 0.2), referred to as *AUPRC-0.2*. It means that we try to evaluate a classifier in accordance with the top scored predictions for true links. So, if the classifier has high precision within this region (subspace), then it is considered to be good classifier and can ensure that those links that are predicted with very high scores are true links.

Unlike the ROC curve, the PR curve is not monotonic and, therefore, the performance can vary by varying the discrimination threshold. The curve is plotted in two-dimensional unit space, with a total area of 1, starts from the point (0,1) and finishes at the point (1,0). There is no PR curve for a default (random) classifier, but some agreement exists on how it could be plotted on a graph [53]. The advantage of PR curves over ROC curves is the fact that, for the former, the class imbalance does not affect correct performance estimation. This is because TNs are excluded from the calculations. The exclusion of TNs, however, breaks the monotonic property of the curve. The PR curve can be very sparse in terms of points when dealing with imbalanced data. Therefore, interpolation is recommended [53,54]. To this end, we employ the implementation by [55].

Similarly to *AUC*, *AUPRC* values range from 0 to 1, while *AUPRC-0.2* values range from 0 to 0.2, where higher values indicate better performance. In the remainder of this article, we refer to the three performance measures introduced here as AUROC, AUPRC, and rAUPRC (restricted AUPRC).

### Ranking of methods

The methods we evaluate are the 114 variants of the CRN approach. An exhaustive list of the variants is given in Appendix A, Table 1-5. The tables (and first letter of the labels used to denote the variants) correspond to the groups of association measures mentioned previously.

To rank the variants of the CRN approach according to their performance on the 47 tasks for GRN inference from time-series data and 39 tasks for GRN inference from steady-state data, we first filter out the low-performing methods by testing the statistical significance of the difference between the measured method performance and the performance of a random classifier. In particular, we employ the one-sample Student *t*test to check whether the average performance of a given method on the GRN inference task is significantly higher than 0.5 and 0.1, respectively, for the expected AUROC and AUPRC performance of a random classifier, and 0.2 (for the rAUPRC performance measure). Methods that are not significantly better than a random classifier with respect to at least one of the three performance measures are excluded from further analysis.

**Figure 1:**
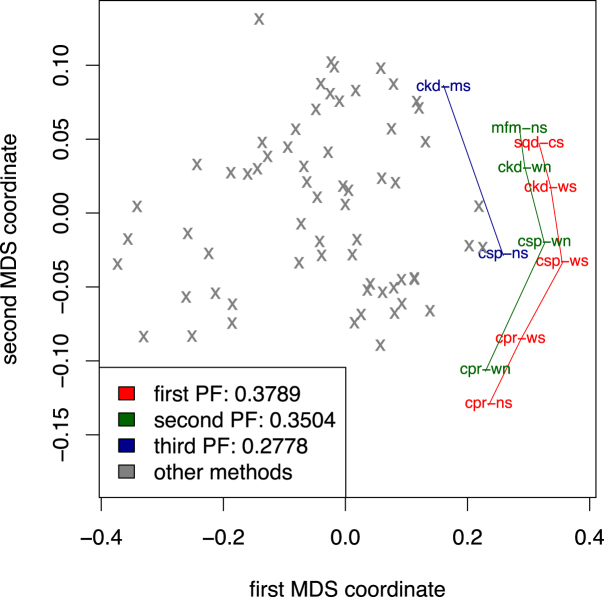
The first three Pareto fronts (PF) in the three-dimensional space of mean rankings of the variants of the CRN approach with respect to the three performance measures of AUROC, AUPRC, and rAUPRC. The rankings of the variants are averaged over all the network reconstruction tasks from Table 1. Each number in the legend represents the hypervolume dominated by the points in the corresponding Pareto front. The two-dimensional projection of the three-dimensional space was obtained using multidimensional scaling.

In the next step, for each performance measure and each GRN inference task, we rank the CRN-approach variants according to their performance on the particular task. Then, we average each method’s ranks over all the tasks to obtain three mean rankings of the CRN-approach variants with respect to the AUROC, AUPRC, and rAUPRC measures. To obtain a joint ranking along the three performance measures, we employ the nondominated sorting algorithm used in multi-objective decision theory [56]. We first embed the variants into a three-dimensional space, where each dimension corresponds to a ranking of the CRN-approach variants with respect to one of the performance measures. Each CRN-approach variant corresponds to a single point in that space, where each coordinate value is the rank of the method according to a particular performance measure. Note that we normalize the method rankings on the [0, 1] scale using a simple linear transformation (*r*_*M*_ − 1)/(*N* − 1), where *r*_*M*_ is the rank of the method *M*, while *N* denotes the number of all compared methods. Figure 1 depicts the projection of the three-dimensional space in two dimensions, obtained by using multidimensional scaling [57]. The red, green, and blue labels and gray points in the graph correspond to the compared CRN-approach variants.

To identify the top-ranked CRN-approach variants, we search for a set of nondominated points in the three-dimensional space, i.e., we identify the Pareto front of the nondominated points in the space. The points in the Pareto front correspond to the methods that are the best performers according to at least one performance measure. After we assign the top ranks to these (Fig. 1) CRN-approach variants, we remove the corresponding points from the three-dimensional space and iteratively continue to identify Pareto fronts in the reduced sets of points until all the methods are ranked.

Table 3 presents the joint ranking along the three performance measures obtained with the nondominated sorting algorithm described above. For each Pareto front, we can calculate the hypervolume of the space dominated by the points on the front. The volume change indicates the magnitude of differences between rankings of the methods in two Pareto fronts. Figure 1 depicts the first three Pareto fronts (red, green, and blue points) in the two-dimensional projection of the original three-dimensional space. They include the 13 top-performing methods according to the three performance measures taken together simultaneously.

**Table 3.** The joint ranking of the CRN-approach variants along the mean rankings with respect to the three performance measures of AUROC, AUPRC, and rAUPRC

PF-I	Dom-HV	CRN-approach variants
1	0.3789	cpr-ns cpr-ws csp-ws ckd-ws sqd-cs
2	0.3504	cpr-wn csp-wn ckd-wn mfm-ns
3	0.2778	csp-ns ckd-ms
4	0.2586	ckd-ns mfm-ws
5	0.2368	mfm-wn saf-wn
6	0.2141	csp-ms mfo-ms mwo-ws mfm-ms saf-ns saw-ns saw-wn saw-ws
7	0.1976	cpr-cs mwo-wn mwo-ms mfm-as sqd-ms saf-ws
8	0.1730	mwo-cs mfs-wn mfs-ms smf-wn saw-cs
9	0.1657	csp-cs mfo-wn mfm-cs mfs-ws smf-ws smw-wn
10	0.1563	ckd-cs mfo-ns mfo-as mfo-ws smw-ns smw-ws
11	0.1339	cpr-ms mfo-cs mwo-ns smf-cs
12	0.1166	mfs-ns smf-ns saf-cs
13	0.1073	ws1-as mfs-as smw-cs
14	0.0943	dmn-as dec-as mfs-cs sss-cs
15	0.0822	d10-as sqd-wn sqd-ws smw-ms saw-ms
16	0.0767	sqd-ns smf-ms saf-ms
17	0.0637	ckd-as mwo-as sqd-as
18	0.0501	csp-as sss-ms
19	0.0293	sss-ns sss-as sss-wn sss-ws

The method rankings are averaged over all the tasks from Table 1. Each row corresponds to a single Pareto front of nondominated points. The column PF-I reports the Pareto front index, Dom-HV reports the volume of the space dominated by the Pareto front, and the last column includes the CRN-approach variants corresponding to the Pareto-front points. The labels of the CRN-approach variants are explained in Appendix A. For example, the third Pareto front consists of two variants *csp-ns* and *ckd-ms*. The first letter of both variants (c) corresponds to *correlation* group of association measures. The second and the third letters describe the association measure itself: *Spearman* and *Kendall* correlation coefficients, respectively, for both variants. Finally, the last two letters refer to a combination of scoring scheme and time-shifting approach, where the first n of the first variant corresponds to *None* (constant or ID scoring scheme applied) and the first letter of the second variant (m) shows that MRNET scoring scheme has been applied. In both cases, the last letter that refers to time shifting use is s, which corresponds to *Spearman* time-shifting approach.

## Results

In the comparative analysis of the performance of the CRN-approach variants, the focus on the top-ranked variants included in the first three Pareto fronts identified with the nondominated sorting algorithm. For each CRN-approach variant in these Pareto fronts, we analyze its composition in terms of the association measure and the scoring scheme employed. We proceed with the analysis as follows. First, we identify the overall top-performing methods on the 47 tasks of GRN inference from time-series data listed in Table 1 and on the 39 tasks of GRN inference from steady-state data listed in Table 2. Next, for experiments with time-series data, we analyze the impact of time-series length and network size on the performance of CRN-approach variants. For the experiments with steady-state data, we analyze the impact of network size only, since the number of records in the dataset (its size) is strongly correlated to the network size (compare the values in the last two columns of Table 2).

### Time-series data

**Figure 2:**
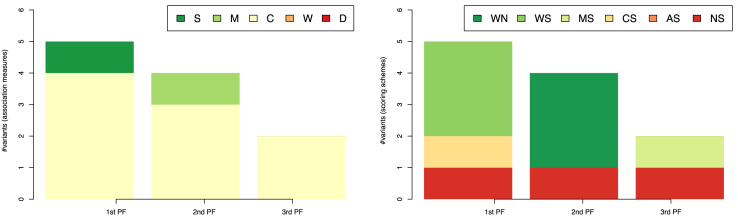
The association measures (left-hand side) and scoring schemes (right-hand side) used by the 11 top-ranked variants of the CRN approach from the first three Pareto fronts (PF) in the three-dimensional space of AUROC-AURPC-rAUPRC mean rankings. The rankings are averaged over the 47 tasks of GRN inference from time-series data listed in Table 1. Legend on the left-hand side: S: the class of symbolic and qualitative association measures; M: association measures based on mutual information; C: correlation-based; W: dynamic time warping; and D: distance-based measures. Legend on the right-hand side: WN: the AWE scoring scheme without time shifting; WS, MS, CS, and AS: the AWE, MRNET, CLR, and ARACNE with time shifting; and NS: the time-shifting method without a scoring scheme.

The comparison of performance on all the tasks of GRN inference from time-series data identifies the correlation-based measures (Fig. 2, left) as the top-performing ones. Correlation-based association measures appear most frequently among the best performers in all three Pareto fronts, dominating by total number of CRN-approach variants. In total, 9 (out of the 11) variants use correlation coefficient in the top three Pareto fronts. In the first Pareto front, correlation-based measures are represented in four (out of five) variants, followed by three appearances (out of four) in the second Pareto front, and two in the third. Variants based on the symbolic and mutual-information measures represent the second most-frequent group in the CRN-approach variants that dominate the performance space. They appear in the first two Pareto fronts, once in each.

In contrast with the clear differences in performance among the association measures, the scoring schemes cannot be so clearly differentiated (Fig. 2, right). Namely, all scoring schemes (except ARACNE) appear within the top three dominant Pareto fronts. The AWE scoring scheme is the most frequent one; it appears in six CRN-approach variants in the first two Pareto fronts. In the first Pareto front, AWE is a component of three variants; in all three variants, it is combined with the time-shifting method (label WS). In the second Pareto front, the AWE scoring scheme is used without the time-shifting method (label WN). The time-shifting method used without scoring scheme (label NS) appears three times among the top-performing CRN-approach variants (once per each Pareto front). Each of the remaining two scoring schemes (MS and CS) appears once among the top-performing CRN-approach variants in the three Pareto fronts.

#### The impact of time-series length

To investigate the impact of time-series length (*l*) on the performance of the CRN-approach variants, we clustered the 47 datasets into two groups of tasks with short (*l* <10, 20 tasks) and long (*l* ≥10, 27 tasks) time series.

**Figure 3:**
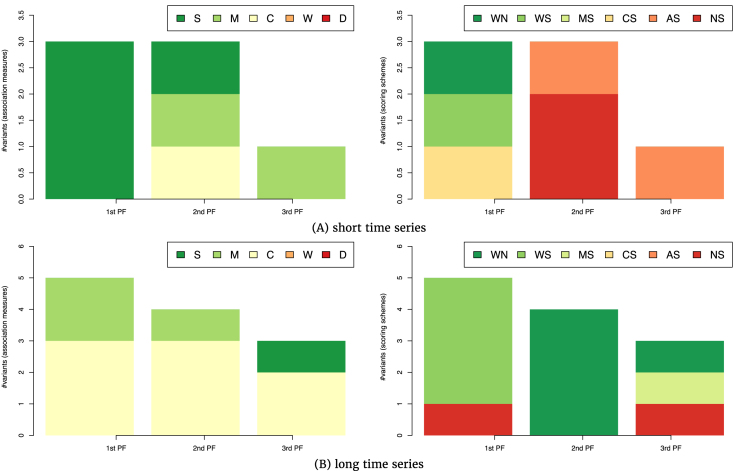
The association measures (left-hand side) and scoring schemes (right-hand side) used in the 7 (A) and 12 (B) top-ranked variants of the CRN approach from the first three Pareto fronts in the three-dimensional space of AUROC-AURPC-rAUPRC mean rankings. The rankings are averaged on the GRN inference tasks involving short (A, 20 tasks) and long (B, 27 tasks) time series.

Figure 3A provides an overview of the seven top-performing CRN-approach variants on the tasks involving short time series. The most frequent association measure among the top performers is the symbolic measure (label S, Fig. 3A, left). Four CRN-approach variants that include symbolic association measures are found in the top two Pareto fronts. Association measures based on mutual information (label M) appear in two variants in the second and third Pareto front. Scoring scheme analysis (Fig. 3A, right) does not show dominance of any particular scoring scheme, except MRNET; all are found among the top-performing variants. The first Pareto front includes two different scoring schemes (labels WN and WS representing AWE as well as CS representing CLR, the latter two combined with time shifting), indicating that the symbolic association measures can be equally well combined with the two scoring schemes of AWE and CLR when tackling tasks involving short time-series data.

The comparison of CRN-approach variants on tasks involving long time series, presented in Fig. 3B, leads to different results. Among the 12 top-ranked CRN-approach variants, the correlation-based association measures prevail; they participate in eight variants distributed among all three Pareto fronts (Fig. 3B, left). Association measures based on mutual information appear in three variants, two of them being in the first Pareto front. A single variant that uses a symbolic association measure is found in the third Pareto front. The results indicate that for long time series, one should prefer correlation over alternative association measures. Differences with respect to the results on short time series are visible for scoring schemes as well (Fig. 3 C, right). The AWE scoring scheme appears in nine variants among the 12 top performers: four times in combination with time shifting and five times without.

In sum, we can conclude that the selection of an appropriate association measure for a given task depends on the time-series length. For short time series, symbolic association measures should be used, while for long ones, one should opt for the correlation-based association measures.

#### The impact of network size

We consider the number of network nodes or genes (*n*) to be a measure of the network size. When analyzing its impact on the performance of the CRN-approach variants, we cluster the GRN inference tasks into two groups of tasks of inferring small (*n* ≤100, 23 tasks) and large (*n* >100, 20 tasks) networks.

**Figure 4:**
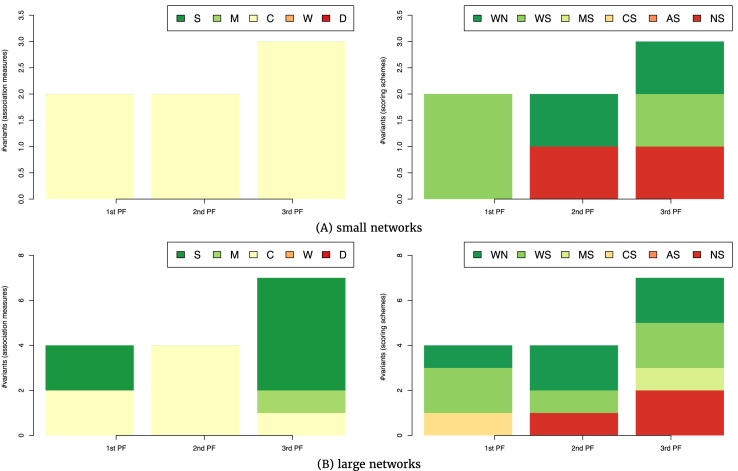
The association measures (left-hand side) and scoring schemes (right-hand side) used in the 7 (A) and 15 (B) top-ranked variants of the CRN approach from the first three Pareto fronts in the three-dimensional space of AUROC-AURPC-rAUPRC mean rankings. The rankings are averaged over the GRN inference tasks involving small (A, 20 tasks of inference from time-series data) and large (B, 27 tasks of inference from time-series data) networks.

Figure 4 depicts the top-performing variants of the CRN approach for tasks involving networks with different sizes. The single top-performing association measure for tasks involving small networks is correlation (see Fig. 4A, left). The distribution of scoring schemes among the seven top-performing variants emphasizes AWE with and without time shifting (labels WS and WN, respectively) present in five out of seven top-ranked variants. Two variants without a scoring scheme appear in the second and third Pareto front (Fig. 4A, right). In sum, for GRN inference tasks involving small networks, one should opt for a combination of a correlation-based association measure and the AWE scoring scheme.

A slightly different distribution of association measures is observed among the top-performing CRN-approach variants on tasks involving large networks (Fig. 4B). Among the 15 top-performing variants approach, 6 employ symbolic association measures, 4 employ measures based on correlation, and a single one in the third Pareto front employs mutual information (Fig. 4 B, left). Similar to the results on the small networks, AWE is prevailing as a scoring scheme used by the top-performing variants (appearing in 11 out of 15), followed by variants without a scoring scheme (Fig. 4B, right).

In sum, the selection of an appropriate association measure for a given task depends on the network size. For tasks involving small networks (up to 100 nodes), correlation-based association measures should be used, while for large ones, one should also consider symbolic measures as another valid option.

### Steady-state data

**Figure 5:**
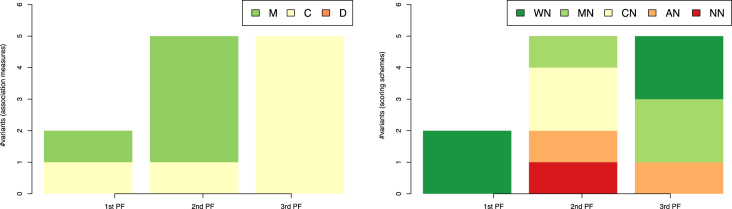
The association measures (left-hand side) and scoring schemes (right-hand side) used by the 12 top-ranked variants of the CRN approach from the first three Pareto fronts (PF) in the three-dimensional space of AUROC-AURPC-rAUPRC mean rankings. The rankings are averaged over the 39 tasks of GRN inference from steady-state data listed in Table 2. Legend on the left-hand side: M: the class of association measures based on mutual information; C: correlation-based and D: distance-based association measures. Legend on the right-hand side: WN, MN, CN, and AN: CRN-approach variants using the AWE, MRNET, CLR, and ARACNE scoring scheme; NN: variants without scoring scheme.

The comparison of performance on all the tasks of GRN inference from steady-state data identifies the CRN-approach variants involving association measures based on correlation and mutual information (Fig. 5, left) as the top-performing ones. Association measures based on mutual information are involved in 5 of the 12 top-performing variants (all 5 being in the first two Pareto fronts). Correlation-based association measures appear among the best performers in all three Pareto fronts, dominating by total number of CRN-approach variants. In total, seven variants using correlation coefficients are found in the leading three Pareto fronts.

In contrast with the notable differences in performance among the variants with different association measures, all scoring schemes can be found among the 12 top-performing variants (Fig. 2, right). The AWE scoring scheme (label WN) can be found in 4 of 11 top-performing variants, while MRNET can be found in 3.

Note that the results obtained on steady-state data tasks resemble the one obtained on tasks involving time-series data. Again, the top-performing CRN-approach variants are the ones using association measures based on correlation and mutual information. Both can be considered valid options for composing a CRN variant to tackle an inference task involving time-series or steady-state data.

**Figure 6:**
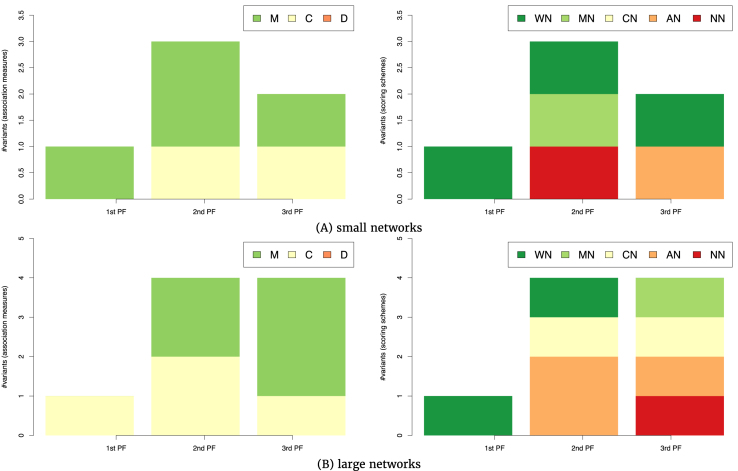
The association measures (left-hand side) and scoring schemes (right-hand side) used in the six (A) and nine (B) top-ranked variants of the CRN approach from the first three Pareto fronts in the three-dimensional space of AUROC-AURPC-rAUPRC mean rankings. The rankings are averaged over the GRN inference tasks involving small (A, 21 tasks of inference from steady-state data) and large (B, 18 tasks of inference from steady-state data) networks.

In contrast, the experimental results of evaluating the impact of network size on the performance of CRN-approach variants on tasks involving steady-state data show no impact of the network size on the performance (Fig. 6). For both small (Fig. 6A, left) and large (Fig. 6B, left) networks, the association measures of choice are based on correlation and mutual information. The results on the selection of the scoring scheme are once again inconclusive, since all scoring schemes are used by the top-performing variants in both groups of tasks involving small and large networks (Fig. 6, right). In any case, we can conclude from Fig. 6 that the network size does not influence the selection of an appropriate CRN-approach variant.

### Discussion

The goal of the discussion presented here is to address the main questions raised in the introduction, i.e., the issue of "What works where?” or *What would be a reasonable choice of association measure and scoring scheme in the generalized relevance network approach for a given task of GRN inference*? The overview of the results of the experiments on all 86 tasks provides a relatively simple answer: the CRN-approach variants using correlation-based associations measure and the AWE scoring scheme perform best. Furthermore, the results show that the AWE scoring scheme works equally well with or without time shifting for inferring link directions from time-series data.

Note that both correlation-based measures and the AWE scoring scheme work over data samples and vectors. This fact already indicates that the particular combination would work equally well for time-series (where the temporal component is largely ignored) and steady-state data. Furthermore, correlation-based measures perform well with other scoring schemes, except for ARACNE. The observation that correlation-based methods, when combined with a certain scoring scheme, yield overall performance improvements on time-series tasks leads to the conclusion that they can perform well with time shifting only, but, in fact, performance improvements can also be gained by selecting an appropriate scoring scheme.

Symbolic association measures have been identified as the second best-performing group of measures that are frequently present among the top-performing CRN-approach variants. In contrast with the correlation-based measures, they operate on temporal data only and are therefore useful only in the context of time-series data. Also, symbolic measures appear to perform well only in combination with the AWE scoring scheme.

Overall, correlation-based association measures show robustness with regard to the selection of a scoring scheme, while the AWE scoring scheme improves performance in general, without limiting the choice of an association measure. For tasks involving steady-state data, top-performing CRN-approach variants also include association measures based on mutual information.

The results of the analysis of method performance on datasets with varying time-series length reveal further *what works where* insights. For short time series, symbolic and mutual information association measures lead to top-performing variants of the CRN approach. Symbolic measures behave robustly and work well in combination with all scoring schemes, except the one that applies time shifting only. This leads to the conclusion that symbolic association measures are robust in general and give more flexibility in choosing a scoring scheme but need to be corrected by a scoring scheme prior to inferring the links directions. Unlike the symbolic association measures, the ones based on mutual information do not show robustness with respect to the selection of a scoring scheme. They perform well only if combined with the ARACNE scoring scheme or the time-shifting method applied without any scoring scheme.

The dominance of the correlation-based association measures increases in the setting of a long time series. Namely, they have been identified in most of the tasks as part of the best-performing method compositions. However, they seem to perform well only in combination with scoring scheme AWE, while time shifting only and MRNET are observed among top performers in one case only. Competing with correlation-based CRN-approach variants are those based on mutual information with strong limitation in choosing a scoring scheme, i.e., AWE with or without time shifting. In sum, when addressing an inference task involving short time series, symbolic association measures are recommended as a robust solution. For long time series, these measures become more dependent on a limited set of scoring schemes. This is a result of the fact that they examine the associations exhaustively throughout the time point’s space. Therefore, for shorter time series, they are capable of complete search of the space, which is not a case for longer time series, where they are constrained due to computational complexity. Conclusively, correlation-based measures can be recommended as a robust solution for long time series, since they are not constrained by the computational complexity issue for retrieval of knowledge from a larger amount of data.

The comparative analysis of method performance over different network sizes shows more consistent results over different settings on the tasks involving steady-state data. Namely, correlation-based association measures outperform all other measures across all network sizes.

For tasks involving inference of small networks from time-series data, the correlation-based group of measures performs well in combination with all scoring schemes, except ARACNE and MRNET. As in the general case, ARACNE performs aggressive cutoffs of inferred links, without considering the difference between estimated associations. However, from these observations, we can conclude that correlation-based association measures are the most robust solution and allow flexibility in choosing a scoring scheme and construction of a customized CRN approach. For tasks involving inference of large networks from time-series data, the association measures of choice are the symbolic ones and mutual information. The former are to be combined with the AWE scoring scheme, while the latter can be used without a scoring scheme or combined with AWE.

Finally, worth mentioning is the observation that distance-based association measures have not been identified among the best-performing association measures in either of the settings considered. Thus, they are excluded from the list of recommended groups of association measures worth considering for tasks of GRN inference from time-series or steady-state data.

## Conclusion

The comparative analysis presented here is based on an extensive empirical evaluation of the performance of 114 variants of the general relevance network approach on 86 tasks of inferring gene regulatory networks from time-series and steady-state data. The 114 CRN-approach variants are based on six general classes of association measures (with a variety of parameter settings) and six scoring schemes, some of which are accompanied by a time-shifting method for inference of the direction of network links from time-series data. The performance of the CRN-approach variants is measured using three different performance metrics widely used in other studies on inferring gene regulatory networks from data.

The main contribution provided here is the general framework for comparative evaluation of the numerous variants of the general relevance network approach to inference of gene regulation networks. The proposed framework is flexible and modular; one can easily extend it along any dimension of comparison, such as adding new association measures, scoring schemes, performance metrics, or network inference tasks. The publicly available source code of the implemented framework allows for simple implementation of such extensions, as well as reproducing the results presented in this study.

The main motivation for the evaluation that we performed is answering the question, "*What works where?*" The answer provides important guidance for applying the generalized relevance network approach in a particular situation in terms of selecting an appropriate combination of an association measure and a scoring scheme that would lead to reasonably good performance on a given dataset. Another important aspect of our survey and comparative analysis is that it involves tasks of inference from both time-series and steady-state data. The results of the comparative analysis lead to the following recommendations for configuring the generalized relevance network approach:

- In general, the safest combination is a correlation-based association measure with the AWE scoring scheme for both time-series and steady-state data.
- The association measures based on simple distances and dynamic time warping never lead to a top-performing variant of the CRN approach.
- For short time series (with less than 10 time points), the general class of symbolic association measures (and the qualitative distance measure, in particular) leads to best-performing variants of the CRN approach. These measures can be combined with an arbitrary scoring scheme.
- For long time series (of at least 10 time points), the general recommendation is to combine a correlation-based association measure with the AWE scoring scheme.
- For large networks with more than 100 nodes, symbolic association measures (combined with the AWE scoring scheme) gain an edge over the correlation-based ones when inducing GRNs from time-series data.

While this set of recommendations provides clear guidance for selecting an appropriate variant of the generalized relevance network approach, further experiments are necessary to strengthen the generality of the results. This is especially true for the results on the impact of network size; too few large-size networks are included in the current set of inference tasks. In future work, one would extend the set of inference tasks with ones that involve networks with a varying number of nodes. Future work could also exploit an important source of input for the relevance network approach, not considered in this study, namely, expert knowledge about the presence or absence of certain links in the network. Note, however, that none of these limitations of our study should represent an obstacle for applying the proposed framework for empirical evaluation. The framework is flexible enough to be used for comparative analysis on extended sets of GRN tasks and CRN-approach variants (methods).

## Availability of supporting data

We implemented all the components and the variants of the CRN approach in the R software environment for statistical computing. We implemented most of the components using standard R functions, except for the association measure based on the dynamic time warping (DTW) measure of distance between time series, for which we used the DTW implementation in the R package *dtw* [36]. The source code of our implementation of the CRN-approach variants in R are publicly available:

- Project name: RN-approach project
- Project home page: https://vkuzmanovski@bitbucket.org/vkuzmanovski/rn-approach.git
- Operating system(s): Platform independent
- Programming language: R
- Other requirements: None
- License: FreeBSD
- RRID: SCR_016488 (SciCrunch.org)

To calculate the values of the performance metrics, we used the functions implemented in the R package for evaluating the performance of classifiers *ROCR* [58]. For performing the Pareto analysis, we used the implementation of the nondominated sorting algorithm in the R package for multi-objective optimization *emoa* [59]. The source code of the R functions used to perform the comparative analysis of the CRN-approach variants that allows for complete reconstruction of its results is also publicly available:

- Project name: RN-evaluation project
- Project home page: https://vkuzmanovski@bitbucket.org/vkuzmanovski/rn-evaluation.git
- Operating system(s): Platform independent
- Programming language: R
- Other requirements: None
- License: FreeBSD

The complete materials, including data and source code, are also available publicly through the GigaDB repository [60].

## Abbreviations

ARACNE: Accurate Reconstruction of Accurate Cellular Networks; AUC: area under the curve; AUPRC: area under the precision-recall curve; AWE: Asymmetric Weighting; CLR: Accurate Reconstruction of Accurate Cellular Networks; CRN: causal relevance network; DTW: dynamic time warping; FN: false negative; FP: false positive; GRN: gene regulatory network; MRNET: Maximum Relevance/minimum redundancy NETwork; PR: precision-recall; ROC: receiver operating characteristic; TN: true negative; TP: true positive.

## Competing interests

The authors declare no competing financial, professional, or personal interests that might have influenced the performance or presentation of the work described in this manuscript.

## Funding

The study was financially supported by the Slovenian Research Agency (through research core funding P2-0103 and P5-0093, as well as project N2-0056, Machine Learning for Systems Sciences), the Slovenian Ministry of Education, Science and Sport (through funding agreement C3330-17-529020), and the European Commission (through grants MAESTRA, HBP SGA2, and LANDMARK).

## Author contributions

S.D. initiated the study and formulated the general methodological problem and the specific application domain. V.K. and L.T. designed and implemented the computational and evaluation framework. All authors conceived and planned the experiments. V.K. and L.T. performed the experiments and analyzed the results. V.K. and L.T. drafted the manuscript. All authors reviewed and approved the final manuscript.

## Supplementary Material

Authors_Response_To_Reviewer_Comments_Revision_1.pdf

GIGA-D-17-00222_(Original_Submission).pdf

GIGA-D-17-00222_Revision_1.pdf

GIGA-D-17-00222_Revision_2.pdf

Response_To_Reviewer_Comments_(Original_Submission).pdf

Reviewer_1_Report_(Original_Submission) -- Frank Emmert-Streib 6/10/2017 Reviewed

Reviewer_2_Report_(Original_Submission) -- Michele Ceccarelli 1/23/2018 Reviewed

Reviewer_2_Report_Revision_1 -- Michele Ceccarelli 7/27/2018 Reviewed

Supplemental File

## APPENDIX A: List of CRN variants

All variants of CRN-approach considered in the study are given in the following tables with full description of their abbreviations. As mentioned before, each variant consists of combination of association measure, scoring scheme and time-shifting, each of which is given as a column in the tables below. All variants are categorized in five categories, based on type of association measure: distance-based measures, dynamic time warping variants, correlation-based measures, mutual information-based measures, and symbolic measures. The last column in each table shows the data type(s) over which particular method has been applied: *ts* refers to ”time-series” and *ss* refers to ”steady-state” data type.

**Table A1.** List of CRN variants within *distance-based* association measures.

Abbreviation	Association measure	Scoring scheme	Time-shifting	Data type
**dmn-nn**	**M**anhatta**n**	**N**one	**N**one	ss
**dmn-ns**	**M**anhatta**n**	**N**one	**S**pearman	ts
**dmn-an**	**M**anhatta**n**	**A**RACNE	**N**one	ss
**dmn-as**	**M**anhatta**n**	**A**RACNE	**S**pearman	ts
**dmn-wn**	**M**anhatta**n**	A**W**E	**N**one	ts,ss
**dmn-ws**	**M**anhatta**n**	A**W**E	**S**pearman	ts
**dmn-cn**	**M**anhatta**n**	**C**LR	**N**one	ss
**dmn-cs**	**M**anhatta**n**	**C**LR	**S**pearman	ts
**dmn-mn**	**M**anhatta**n**	**M**RNET	**N**one	ss
**dmn-ms**	**M**anhatta**n**	**M**RNET	**S**pearman	ts
**dec-nn**	**E**u**c**lidean	**N**one	**N**one	ss
**dec-ns**	**E**u**c**lidean	**N**one	**S**pearman	ts
**dec-an**	**E**u**c**lidean	**A**RACNE	**N**one	ss
**dec-as**	**E**u**c**lidean	**A**RACNE	**S**pearman	ts
**dec-wn**	**E**u**c**lidean	A**W**E	**N**one	ts,ss
**dec-ws**	**E**u**c**lidean	A**W**E	**S**pearman	ts
**dec-cn**	**E**u**c**lidean	**C**LR	**N**one	ss
**dec-cs**	**E**u**c**lidean	**C**LR	**S**pearman	ts
**dec-mn**	**E**u**c**lidean	**M**RNET	**N**one	ss
**dec-ms**	**E**u**c**lidean	**M**RNET	**S**pearman	ts
**d10-nn**	L-**10** norm (Minkowsky)	**N**one	**N**one	ss
**d10-ns**	L-**10** norm (Minkowsky)	**N**one	**S**pearman	ts
**d10-an**	L-**10** norm (Minkowsky)	**A**RACNE	**N**one	ss
**d10-as**	L-**10** norm (Minkowsky)	**A**RACNE	**S**pearman	ts
**d10-wn**	L-**10** norm (Minkowsky)	A**W**E	**N**one	ts,ss
**d10-ws**	L-**10** norm (Minkowsky)	A**W**E	**S**pearman	ts
**d10-cn**	L-**10** norm (Minkowsky)	**C**LR	**N**one	ss
**d10-cs**	L-**10** norm (Minkowsky)	**C**LR	**S**pearman	ts
**d10-mn**	L-**10** norm (Minkowsky)	**M**RNET	**N**one	ss
**d10-ms**	L-**10** norm (Minkowsky)	**M**RNET	**S**pearman	ts

**Table A2.** List of CRN variants within *dynamic time warping (DTW)* association measure with variants.

Abbreviation	Association measure	Scoring scheme	Time-shifting	Data type
**was-ns**	DT**W** - **As**ymmetric constraint path	**N**one	**S**pearman	ts
**was-as**	DT**W** - **As**ymmetric constraint path	**A**RACNE	**S**pearman	ts
**was-wn**	DT**W** - **As**ymmetric constraint path	A**W**E	**N**one	ts
**was-ws**	DT**W** - **As**ymmetric constraint path	A**W**E	**S**pearman	ts
**was-cs**	DT**W** - **As**ymmetric constraint path	**C**LR	**S**pearman	ts
**was-ms**	DT**W** - **As**ymmetric constraint path	**M**RNET	**S**pearman	ts
**ws1-ns**	DT**W** - **S**ymmetric constraint path (**1**)	**N**one	**S**pearman	ts
**ws1-as**	DT**W** - **S**ymmetric constraint path (**1**)	**A**RACNE	**S**pearman	ts
**ws1-wn**	DT**W** - **S**ymmetric constraint path (**1**)	A**W**E	**N**one	ts
**ws1-ws**	DT**W** - **S**ymmetric constraint path (**1**)	A**W**E	**S**pearman	ts
**ws1-cs**	DT**W** - **S**ymmetric constraint path (**1**)	**C**LR	**S**pearman	ts
**ws1-ms**	DT**W** - **S**ymmetric constraint path (**1**)	**M**RNET	**S**pearman	ts
**ws2-ns**	DT**W** - **S**ymmetric constraint path (**2**)	**N**one	**S**pearman	ts
**ws2-as**	DT**W** - **S**ymmetric constraint path (**2**)	**A**RACNE	**S**pearman	ts
**ws2-wn**	DT**W** - **S**ymmetric constraint path (**2**)	A**W**E	**N**one	ts
**ws2-ws**	DT**W** - **S**ymmetric constraint path (**2**)	A**W**E	**S**pearman	ts
**ws2-cs**	DT**W** - **S**ymmetric constraint path (**2**)	**C**LR	**S**pearman	ts
**ws2-ms**	DT**W** - **S**ymmetric constraint path (**2**)	**M**RNET	**S**pearman	ts

**Table A3.** List of CRN variants within *correlation-based* association measures.

Abbreviation	Association measure	Scoring scheme	Time-shifting	Data type
**cpr-nn**	**P**ea**r**son	**N**one	**N**one	ss
**cpr-ns**	**P**ea**r**son	**N**one	**S**pearman	ts
**cpr-an**	**P**ea**r**son	**A**RACNE	**N**one	ss
**cpr-as**	**P**ea**r**son	**A**RACNE	**S**pearman	ts
**cpr-wn**	**P**ea**r**son	A**W**E	**N**one	ts,ss
**cpr-ws**	**P**ea**r**son	A**W**E	**S**pearman	ts
**cpr-cn**	**P**ea**r**son	**C**LR	**N**one	ss
**cpr-cs**	**P**ea**r**son	**C**LR	**S**pearman	ts
**cpr-mn**	**P**ea**r**son	**M**RNET	**N**one	ss
**cpr-ms**	**P**ea**r**son	**M**RNET	**S**pearman	ts
**csp-nn**	**Sp**earman	**N**one	**N**one	ss
**csp-ns**	**Sp**earman	**N**one	**S**pearman	ts
**csp-an**	**Sp**earman	**A**RACNE	**N**one	ss
**csp-as**	**Sp**earman	**A**RACNE	**S**pearman	ts
**csp-wn**	**Sp**earman	A**W**E	**N**one	ts,ss
**csp-ws**	**Sp**earman	A**W**E	**S**pearman	ts
**csp-cn**	**Sp**earman	**C**LR	**N**one	ss
**csp-cs**	**Sp**earman	**C**LR	**S**pearman	ts
**csp-mn**	**Sp**earman	**M**RNET	**N**one	ss
**csp-ms**	**Sp**earman	**M**RNET	**S**pearman	ts
**ckd-nn**	**K**en**d**all	**N**one	**N**one	ss
**ckd-ns**	**K**en**d**all	**N**one	**S**pearman	ts
**ckd-an**	**K**en**d**all	**A**RACNE	**N**one	ss
**ckd-as**	**K**en**d**all	**A**RACNE	**S**pearman	ts
**ckd-wn**	**K**en**d**all	A**W**E	**N**one	ts,ss
**ckd-ws**	**K**en**d**all	A**W**E	**S**pearman	ts
**ckd-cn**	**K**en**d**all	**C**LR	**N**one	ss
**ckd-cs**	**K**en**d**all	**C**LR	**S**pearman	ts
**ckd-mn**	**K**en**d**all	**M**RNET	**N**one	ss
**ckd-ms**	**K**en**d**all	**M**RNET	**S**pearman	ts

**Table A4.** List of CRN variants within *mutual information-based* association measures.

Abbreviation	Association measure	Scoring scheme	Time-shifting	Data type
**mfo-nn**	**M**utual information - Equal **f**requency (Miller-Madow estimator)	**N**one	**N**one	ss
**mfo-ns**	**M**utual information - Equal **f**requency (Miller-Madow estimator)	**N**one	**S**pearman	ts
**mfo-an**	**M**utual information - Equal **f**requency (Miller-Madow estimator)	**A**RACNE	**N**one	ss
**mfo-as**	**M**utual information - Equal **f**requency (Miller-Madow estimator)	**A**RACNE	**S**pearman	ts
**mfo-wn**	**M**utual information - Equal **f**requency (Miller-Madow estimator)	A**W**E	**N**one	ts,ss
**mfo-ws**	**M**utual information - Equal **f**requency (Miller-Madow estimator)	A**W**E	**S**pearman	ts
**mfo-cn**	**M**utual information - Equal **f**requency (Miller-Madow estimator)	**C**LR	**N**one	ss
**mfo-cs**	**M**utual information - Equal **f**requency (Miller-Madow estimator)	**C**LR	**S**pearman	ts
**mfo-mn**	**M**utual information - Equal **f**requency (Miller-Madow estimator)	**M**RNET	**N**one	ss
**mfo-ms**	**M**utual information - Equal **f**requency (Miller-Madow estimator)	**M**RNET	**S**pearman	ts
**mwo-nn**	**M**utual information - Equal **w**idth (Miller-Madow estimator)	**N**one	**N**one	ss
**mwo-ns**	**M**utual information - Equal **w**idth (Miller-Madow estimator)	**N**one	**S**pearman	ts
**mwo-an**	**M**utual information - Equal **w**idth (Miller-Madow estimator)	**A**RACNE	**N**one	ss
**mwo-as**	**M**utual information - Equal **w**idth (Miller-Madow estimator)	**A**RACNE	**S**pearman	ts
**mwo-wn**	**M**utual information - Equal **w**idth (Miller-Madow estimator)	A**W**E	**N**one	ts,ss
**mwo-ws**	**M**utual information - Equal **w**idth (Miller-Madow estimator)	A**W**E	**S**pearman	ts
**mwo-cn**	**M**utual information - Equal **w**idth (Miller-Madow estimator)	**C**LR	**N**one	ss
**mwo-cs**	**M**utual information - Equal **w**idth (Miller-Madow estimator)	**C**LR	**S**pearman	ts
**mwo-mn**	**M**utual information - Equal **w**idth (Miller-Madow estimator)	**M**RNET	**N**one	ss
**mwo-ms**	**M**utual information - Equal **w**idth (Miller-Madow estimator)	**M**RNET	**S**pearman	ts
**mfm-nn**	**M**utual information - Equal **f**requency (**M**aximum likelihood estimator)	**N**one	**N**one	ss
**mfm-ns**	**M**utual information - Equal **f**requency (**M**aximum likelihood estimator)	**N**one	**S**pearman	ts
**mfm-an**	**M**utual information - Equal **f**requency (**M**aximum likelihood estimator)	**A**RACNE	**N**one	ss
**mfm-as**	**M**utual information - Equal **f**requency (**M**aximum likelihood estimator)	**A**RACNE	**S**pearman	ts
**mfm-wn**	**M**utual information - Equal **f**requency (**M**aximum likelihood estimator)	A**W**E	**N**one	ts,ss
**mfm-ws**	**M**utual information - Equal **f**requency (**M**aximum likelihood estimator)	A**W**E	**S**pearman	ts
**mfm-cn**	**M**utual information - Equal **f**requency (**M**aximum likelihood estimator)	**C**LR	**N**one	ss
**mfm-cs**	**M**utual information - Equal **f**requency (**M**aximum likelihood estimator)	**C**LR	**S**pearman	ts
**mfm-mn**	**M**utual information - Equal **f**requency (**M**aximum likelihood estimator)	**M**RNET	**N**one	ss
**mfm-ms**	**M**utual information - Equal **f**requency (**M**aximum likelihood estimator)	**M**RNET	**S**pearman	ts
**mfs-nn**	**M**utual information - Equal **f**requency (**S**hrink entropy estimator)	**N**one	**N**one	ss
**mfs-ns**	**M**utual information - Equal **f**requency (**S**hrink entropy estimator)	**N**one	**S**pearman	ts
**mfs-an**	**M**utual information - Equal **f**requency (**S**hrink entropy estimator)	**A**RACNE	**N**one	ss
**mfs-as**	**M**utual information - Equal **f**requency (**S**hrink entropy estimator)	**A**RACNE	**S**pearman	ts
**mfs-wn**	**M**utual information - Equal **f**requency (**S**hrink entropy estimator)	A**W**E	**N**one	ts,ss
**mfs-ws**	**M**utual information - Equal **f**requency (**S**hrink entropy estimator)	A**W**E	**S**pearman	ts
**mfs-cn**	**M**utual information - Equal **f**requency (**S**hrink entropy estimator)	**C**LR	**N**one	ss
**mfs-cs**	**M**utual information - Equal **f**requency (**S**hrink entropy estimator)	**C**LR	**S**pearman	ts
**mfs-mn**	**M**utual information - Equal **f**requency (**S**hrink entropy estimator)	**M**RNET	**N**one	ss
**mfs-ms**	**M**utual information - Equal **f**requency (**S**hrink entropy estimator)	**M**RNET	**S**pearman	ts

**Table A5.** List of CRN variants within *symbolic* association measures.

Abbreviation	Association measure	Scoring scheme	Time-shifting	Data type
**sqd-ns**	Simple **q**ualitative **d**istance	**N**one	**S**pearman	ts
**sqd-as**	Simple **q**ualitative **d**istance	**A**RACNE	**S**pearman	ts
**sqd-wn**	Simple **q**ualitative **d**istance	A**W**E	**N**one	ts
**sqd-ws**	Simple **q**ualitative **d**istance	A**W**E	**S**pearman	ts
**sqd-cs**	Simple **q**ualitative **d**istance	**C**LR	**S**pearman	ts
**sqd-ms**	Simple **q**ualitative **d**istance	**M**RNET	**S**pearman	ts
**sss-ns**	**S**ymbol **s**equence similarity	**N**one	**S**pearman	ts
**sss-as**	**S**ymbol **s**equence similarity	**A**RACNE	**S**pearman	ts
**sss-wn**	**S**ymbol **s**equence similarity	A**W**E	**N**one	ts
**sss-ws**	**S**ymbol **s**equence similarity	A**W**E	**S**pearman	ts
**sss-cs**	**S**ymbol **s**equence similarity	**C**LR	**S**pearman	ts
**sss-ms**	**S**ymbol **s**equence similarity	**M**RNET	**S**pearman	ts
**smw-ns**	**M**utual information (equal **w**idth) over symbol vectors	**N**one	**S**pearman	ts
**smw-as**	**M**utual information (equal **w**idth) over symbol vectors	**A**RACNE	**S**pearman	ts
**smw-wn**	**M**utual information (equal **w**idth) over symbol vectors	A**W**E	**N**one	ts
**smw-ws**	**M**utual information (equal **w**idth) over symbol vectors	A**W**E	**S**pearman	ts
**smw-cs**	**M**utual information (equal **w**idth) over symbol vectors	**C**LR	**S**pearman	ts
**smw-ms**	**M**utual information (equal **w**idth) over symbol vectors	**M**RNET	**S**pearman	ts
**smf-ns**	**M**utual information (equal **f**requency) over symbol vectors	**N**one	**S**pearman	ts
**smf-as**	**M**utual information (equal **f**requency) over symbol vectors	**A**RACNE	**S**pearman	ts
**smf-wn**	**M**utual information (equal **f**requency) over symbol vectors	A**W**E	**N**one	ts
**smf-ws**	**M**utual information (equal **f**requency) over symbol vectors	A**W**E	**S**pearman	ts
**smf-cs**	**M**utual information (equal **f**requency) over symbol vectors	**C**LR	**S**pearman	ts
**smf-ms**	**M**utual information (equal **f**requency) over symbol vectors	**M**RNET	**S**pearman	ts
**saw-ns**	**A**verage of *sss-ns* and *smw-ns*	**N**one	**S**pearman	ts
**saw-as**	**A**verage of *sss-as* and *smw-as*	**A**RACNE	**S**pearman	ts
**saw-wn**	**A**verage of *sss-wn* and *smw-wn*	A**W**E	**N**one	ts
**saw-ws**	**A**verage of *sss-ws* and *smw-ws*	A**W**E	**S**pearman	ts
**saw-cs**	**A**verage of *sss-cs* and *smw-cs*	**C**LR	**S**pearman	ts
**saw-ms**	**A**verage of *sss-ms* and *smw-ms*	**M**RNET	**S**pearman	ts
**saf-ns**	**A**verage of *sss-ns* and *smf-ns*	**N**one	**S**pearman	ts
**saf-as**	**A**verage of *sss-as* and *smf-as*	**A**RACNE	**S**pearman	ts
**saf-wn**	**A**verage of *sss-wn* and *smf-wn*	A**W**E	**N**one	ts
**saf-ws**	**A**verage of *sss-ws* and *smf-ws*	A**W**E	**S**pearman	ts
**saf-cs**	**A**verage of *sss-cs* and *smf-cs*	**C**LR	**S**pearman	ts
**saf-ms**	**A**verage of *sss-ms* and *smf-ms*	**M**RNET	**S**pearman	ts
