# Supplementary material for: Extensive evaluation of the generalized relevance network approach to inferring gene regulatory networks
Source: Gigascience. 2018 Sep 18;7(11):giy118. doi: 10.1093/gigascience/giy118 (PMC6420648; doi:10.1093/gigascience/giy118)
Supplement: GIGA-D-17-00222_(Original_Submission).pdf [file giy118_giga-d-17-00222_(original_submission).pdf]

GigaScience

Extensive evaluation of the relevance network approach to inferring gene regulatory networks

--Manuscript Draft--

**Manuscript Number:**	GIGA-D-17-00222
**Full Title:**	Extensive evaluation of the relevance network approach to inferring gene regulatory networks
**Article Type:**	Research
**Funding Information:**	Javna Agencija za Raziskovalno Dejavnost RS (P2-0103) — Dr Vladimir Kuzmanovski, Prof. Dr. Sašo Džeroski<br>Javna Agencija za Raziskovalno Dejavnost RS (P5-0093) — Dr Ljupčo Todorovski<br>Javna Agencija za Raziskovalno Dejavnost RS (N2-0056) — Dr Ljupčo Todorovski, Prof. Dr. Sašo Džeroski<br>European Commission (ICT-2013-612944 MAESTRA) — Dr Ljupčo Todorovski, Prof. Dr. Sašo Džeroski
**Abstract:**	The relevance network approach to network inference reconstructs the network links based on the strength of associations between data in individual network nodes. It is a general algorithm that allows the use of an arbitrary measure of pairwise association between nodes, an arbitrary scoring scheme that transforms the associations into weights of the network links, and a method for inferring the directions of the network links. While this makes the relevance network approach powerful and flexible, it introduces the challenge of finding a combination of components that would perform well on a given inference task. This paper addresses this challenge by performing an extensive empirical analysis of the performance of 114 variants of the general relevance network approach on 38 tasks of gene network inference from time-series data. The results of the evaluation reveal that the association measures based on correlation combined with a particular scoring scheme of asymmetric weighting lead to optimal performance of the relevance network approach in the general case. In the two special cases of inference tasks involving short time-series data and/or large networks, association measures based on identifying qualitative trends in the time series are more appropriate.
**Corresponding Author:**	Vladimir Kuzmanovski, Ph.D.<br>Institut Jozef Stefan<br>Ljubljana, SLOVENIA
**Corresponding Author Secondary Information:**	
**Corresponding Author's Institution:**	Institut Jozef Stefan
**Corresponding Author's Secondary Institution:**	
**First Author:**	Vladimir Kuzmanovski, Ph.D.
**First Author Secondary Information:**	
**Order of Authors:**	Vladimir Kuzmanovski, Ph.D.<br>Ljupčo Todorovski<br>Sašo Džeroski
**Order of Authors Secondary Information:**	
**Opposed Reviewers:**	
**Additional Information:**	

Powered by Editorial Manager® and ProduXion Manager® from Aries Systems Corporation

Question	Response
Are you submitting this manuscript to a special series or article collection?	No
**Experimental design and statistics**<br><br>Full details of the experimental design and statistical methods used should be given in the Methods section, as detailed in our Minimum Standards Reporting Checklist. Information essential to interpreting the data presented should be made available in the figure legends.<br><br>Have you included all the information requested in your manuscript?	Yes
**Resources**<br><br>A description of all resources used, including antibodies, cell lines, animals and software tools, with enough information to allow them to be uniquely identified, should be included in the Methods section. Authors are strongly encouraged to cite Research Resource Identifiers (RRIDs) for antibodies, model organisms and tools, where possible.<br><br>Have you included the information requested as detailed in our Minimum Standards Reporting Checklist?	Yes
**Availability of data and materials**<br><br>All datasets and code on which the conclusions of the paper rely must be either included in your submission or deposited in publicly available repositories (where available and ethically appropriate), referencing such data using a unique identifier in the references and in the "Availability of Data and Materials" section of your manuscript.<br><br>Have you have met the above requirement as detailed in our Minimum Standards Reporting Checklist?	Yes

Manuscript
Click here to download Manuscript main.tex

OXFORD
(GIGA)^n SCIENCE

GigaScience, 2017, 1–15

doi: xx.xxxx/xxxx
Manuscript in Preparation
Paper

PAPER

Extensive evaluation of the relevance network approach to inferring gene regulatory networks

Vladimir Kuzmanovski[1,*], Ljupčo Todorovski[1,2] and Sašo Džeroski[1]

[1]Jožef Stefan Institute, Ljubljana,Slovenia and [2]University of Ljubljana, Slovenia

[*]vladimir.kuzmanovski@ijs.si

Abstract

The relevance network approach to network inference reconstructs the network links based on the strength of associations between data in individual network nodes. It is a general algorithm that allows the use of an arbitrary measure of pairwise association between nodes, an arbitrary scoring scheme that transforms the associations into weights of the network links, and a method for inferring the directions of the network links. While this makes the relevance network approach powerful and flexible, it introduces the challenge of finding a combination of components that would perform well on a given inference task. This paper addresses this challenge by performing an extensive empirical analysis of the performance of 114 variants of the general relevance network approach on 38 tasks of gene network inference from time-series data. The results of the evaluation reveal that the association measures based on correlation combined with a particular scoring scheme of asymmetric weighting lead to optimal performance of the relevance network approach in the general case. In the two special cases of inference tasks involving short time-series data and/or large networks, association measures based on identifying qualitative trends in the time series are more appropriate.

Key words: network reconstruction; network inference; relevance network approach; gene regulatory networks

Introduction

The genome of an organism plays a central role in the control of cellular processes in the organism. Genes do not work in isolation, but work in highly connected and structured networks of information flow through a cell. Such biological networks are typically referred to as gene regulatory networks (GRNs). The inference of GRNs using computational and statistical methods gains a lot of attention in contemporary biology when data can be massively collected for a relatively low price.

Inference of GRNs is a reverse engineering task [1] that comprises identification of pairwise interactions between molecules participating in the same biological processes or performing specific biological functions together [2]. In general, the data for GRN inference comes from microarray experiments perturbing and stressing genes that produce highly resolved time-series and steady-state measurements of transcript levels. Such captured dynamic response of transcription and translation within a cell can provide robust information about the GRN under consideration [3].

Literature review discovers the existence of a wide range of approaches for inferring GRNs that try to infer true links out of all available data [4, 5, 6, 7]. Throughout the review articles, the authors cluster the approaches or methods into categories that reflect the way links are inferred, resulting in few different categorizations. The most general one, groups the approaches to network inference in the two broad categories of model-based and lazy (unsupervised) methods, where the group of model-based approaches is further split into supervised and semi-supervised methods [4, 1].

As the name suggests, model-based approaches build a model that capture the knowledge from data and use the model to predict existence of a link between two genes. On the other hand, the lazy approaches capture the knowledge directly from data and instantly use the captured knowledge to predict or estimate existence of a network link. Both categories of approaches operate on two types of data: static data records expression levels of the pseudo state reached after the perturbation and time-course data that record gene expression levels

Compiled on: September 3, 2017.
Draft manuscript prepared by the author.

Key Points

- Recommendations for selecting an appropriate variant of the relevance network approach for a network inference task
- Comprehensive survey of the variants of the relevance network approach to network reconstruction
- Comparison of the performance of 114 variants of the relevance network approach on 38 tasks of gene networks inference along multiple performance metrics simultaneously

over a certain period of time after the perturbation [8].

Our paper focus on the relevance-network approach [8], a lazy approach to network inference that predicts network links based on the pairwise associations between genes. The degree of a pairwise association is evaluated using a measure of similarity between the time series of the expression levels of the two corresponding genes. The variety of similarity measures that can be applied within the relevance-network approach makes the approach flexible and applicable in various scenarios. In contrast with other surveys that focus on its flexibility by presenting and categorizing the variants of the network approach, the survey presented here focuses on the issue of choosing an appropriate variant of the relevance network approach for given data.

To address this issue, we perform an extensive comparative analysis of the performance of 114 variants of the relevance network approach on 38 tasks of inferring gene regulatory networks from time-series data. The tasks include inference from real microarray measurements of the microorganism Saccharomyces cerevisiae (Yeast) as well as simulations of artificial networks in Yeast and Escherichia coli. We analyze the impact of time-series length and network size on the performance of the different variants of the relevance network approach. The performance of network inference is measured and compared using three different performance matrices used widely in the literature on network reconstruction.

The study is organized as follows. First, we introduce the relevance network approach and overview its variants that stem from different measures of association between network nodes. The survey of the variants of the relevance network approach expose their theoretical advantages and disadvantages as well as the history of their applications for inference of gene regulatory networks. Section 3 introduces the experimental setup of the comparative analysis in terms of the GRN tasks addressed, data sets employed and metrics used to measure the performance of the network inference methods. Section 4 presents and discusses the results with the emphasis on what is the most appropriate variant of the relevance network approach for a given GRN inference task. Finally, Section 5 concludes the paper with the brief summary of the comparative analysis and outline of the directions for further research.

Relevance network approach

Relevance network (RN) approach infers knowledge about network structure by measuring the pairwise associations between the data observed in the individual network nodes. No explicit model of the data is built, so the RN approach belongs to the cluster of lazy approaches to network inference. The retrieved knowledge about the pairwise association between nodes is interpreted as a relevance of the individual network links [9, 4].

The early variants of the RN approach Rays et al. [10], Eisen et al. [11] employed Euclidean distance and correlation coefficient to measure the pairwise associations between time series of RNA expression levels. Later on, Butte and Kohane [12] introduces mutual information as a measure of association between genes and references to the approach as "relevance network". In recent studies, RN approach has been extended to include other association measures and additional steps of interpreting the measured associations, such as the symmetry-breaking methods or methods for marginal control of the association [8, 1, 13]. Hempel et al. [8] decomposed the RN approach into three components of (1) inference of pairwise associations, (2) marginal control of association, and (3) breaking symmetry. The first step employs a distance measure, a correlation coefficient or a mutual-information measure to assess the association between two network nodes. The result of the first step is a symmetric matrix, the element of which indicate the strength of the undirected network links. The second step of the marginal control of the association scores includes various scoring schemes, which transform the association-scores matrix into symmetric or asymmetric matrix of the weights of the network links. The last component, as its name indicates, breaks the symmetry of the symmetric network weights matrices, typically using a time-shifting approach.

Overall, the main idea behind the RN approach is to assign a higher relevance to a hypothesized network link, which is identified with a strong pair-wise association between the corresponding nodes. Thus, the relevance score provides an opportunity for differentiating the possibilities of existence of individual network links. Finally, to obtain the inferred network structure, one has to decide upon a threshold value used to map the numeric relevance score into a discrete binary value the indicate the validity of the initial hypothesis, i.e., the presence of a network link.

The reminder of the section follows the decomposition of the RN approach into the three components introduced above. First, we introduce all the measures used to estimate the pairwise association between nodes in our comparative study of the RN-approach variants. Next, we introduce the scoring schemes for marginal control of association. Finally, we introduce the time-shifting method for breaking the symmetry of the association/weight matrices.

Association measures

Association measures used in this study can be broadly categorized into three categories: correlation-based, information-based, and distance-based measures. Correlation-based measures treat the given time series as data samples and calculate the association as a correlation coefficient between them. Information-base measures treat the given time series as random variables and calculate the association as a non-linear dependence quantified by their mutual information. Distance-based measures calculate the association as an inverse of the distance between the observed time series and can be further clustered in three subgroups. Simple distance measures in the first group treat time series as vectors. The second group includes a single distance measure of dynamic time wrapping that operates on time series directly. The symbolic measures in the third group operate on symbolic (or qualitative) representation of the time-series trends.

In each of the following subsections, we are going to present

a single group of association measures as introduced above. Throughout these section, we are going to use the Greek lowercase letters μ to denote the pairwise association between the time series and δ for the distance between the time series.

Correlation-based measures

Correlation-based measures consider the expression time-series $x = \langle x_1, x_2, \ldots x_n \rangle$ and $y = \langle y_1, y_2, \ldots y_n \rangle$ as population samples. This allows for employment of an arbitrary correlation coefficient measured over samples. In particular, we use three of them in this study: Pearson correlation coefficient, Spearman and Kendall rank correlation coefficients.

Person correlation coefficient quantifies the linear relationship between the samples x and y as

$$\mu_P(x,y) = \frac{\sum_{k=1}^{n}(x_k - \bar{x})(y_k - \bar{y})}{\sqrt{\sum_{k=1}^{n}(x_k - \bar{x})^2} \cdot \sqrt{\sum_{k=1}^{n}(y_k - \bar{y})^2}}, \quad (1)$$

where $\bar{x}$ and $\bar{y}$ are the sample means of x and y, respectively.

As mentioned before, Pearson correlation coefficient has been employed within the RN approach by Eisen et al. [11]. Later on, it has been regularly used as a state-of-the-art association measure being integrated and compared with other methods [8, 14, 4].

Spearman rank correlation coefficient is based on the rank distribution of the observed expression values. It can be used as a more general measure of inter-dependencies that is not restricted to linear relationships and defines the inter-dependency between x and y as:

$$\mu_S(x,y) = \mu_P(R(x), R(y)) \quad (2)$$

where $R(u) = \langle r(u_1), r(u_2), \ldots r(u_n) \rangle$ and $r(u_k)$ denotes the rank of u_k in u.

The Spearman's rank correlation is often used as an association measure in variants of the RN approach [15, 16, 4].

Kendall rank correlation coefficient is another measure of correlation between ranks of two samples x and y defined as [17]:

$$\mu_K(x,y) = \frac{2(n_c - n_d)}{n(n-1)}, \quad (3)$$

where n_c is the number of concordant pairs of points in x and y, while n_d is the number of discordant pairs. A concordant pair of time points i and j is concordant, if both $x_i > x_j$ and $y_i > y_j$ or both $x_i < x_j$ and $y_i < y_j$. Otherwise, the pair is discordant.

The Kendall's rank correlation is rarely used as an association measure in the variants of the RN approach, with a few notable exceptions in recent studies [8].

Correlation-based measures applied for inferring associations among genes in a GRN have been widely used in the domain of network reconstruction. The rank correlation coefficient are characterized with the fact that do not consider continuous scale of expression vectors, instead consider them as vector of values or ranks (ranking can be considered as a kind of discretization). Furthermore, correlation-based measures dismiss the time-component of the time-series data. Finally, note that the resulting pairwise association matrices are symmetric and can not infer the direction of the network links.

Information-based measures

Information-based or information-theoretic measures the association between the expression time-series $x = \langle x_1, x_2, \ldots x_n \rangle$ by considering them to be random variables. Most commonly used metric, also and used in our study, is mutual information, also referred to as simple mutual information [18]. In our study, we employ the mutual information in a combination with different statistical estimators of entropy and different discretization methods, introduced below.

Mutual information is a measure to quantify non-linear inter-dependencies between two random variables X and Y. It is computationally tractable by using different entropy estimators, but usually fails to discover indirect links, instead represents them as direct links between the nodes in the reconstructed network [19]. The general form of the mutual information (MI) expresses the marginal entropies $H(X)$ and $H(Y)$, and joint entropy $H(X,Y)$:

$$\mu_I(x,y) = H(X) + H(Y) - H(X,Y). \quad (4)$$

In the domain of GRN inference, three estimators of entropy of a given random variable are being widely used Paninski [20], Meyer et al. [21] and de Matos Simoes and Emmert-Streib [18]: maximum likelihood estimator, Miller-Madow estimator, and shrink entropy estimator.

Maximum likelihood estimator assesses the entropy of a given empirical distribution of a random variable X and follows the Shannon entropy definition [20]:

$$H^{emp}(X) = -\sum_{k=1}^{n} p(x_k) \cdot log(p(x_k)). \quad (5)$$

This estimator is highly dependent on the number of bins n and the length of observations, which can cause enlarging the bias, while the estimator variance is being kept minimal.

Miller-Madow estimator is based on maximum likelihood estimator, but corrected with a second additive term representing the asymptotic bias:

$$H^{mm}(X) = H^{emp}(X) + \frac{|X| - 1}{2n}, \quad (6)$$

where $|X|$ is the number of beans with non-zero probability. The Miller-Madow estimator is preferred over maximum likelihood estimator due to reduction of the bias without changing variance, and without additional computational cost [20].

Shrink entropy estimator [22] uses a general regularization technique to improve the maximum likelihood estimator. The idea is to combine two different estimators, one with low variance and another one with low bias, by using a shrinking factor $\lambda \in [0,1]$. For given random variable X, the entropy is estimated as follows:

$$H^{shrink}(X) = -\sum_{k=1}^{n} p_\lambda(x_k) \cdot log(p_\lambda(x_k)), \quad (7)$$

where p_λ is defined as follows:

$$p_\lambda(x_k) = \lambda \frac{1}{|X|} + (1-\lambda)p(x_k). \quad (8)$$

If the value of λ is close to zero, the estimated entropy is close to the value of maximum likelihood estimator, otherwise, if it is close to one, then the entropy estimation tends to be closer to the bias term.

The statistical estimators are combined with two different methods for discretization of numeric random variables: equal width and equal frequency. All six possible combinations of estimators and discretization methods are being used in the comparative analysis of the RN-approach variants.

Equal width is a fixed bin-width discretization method, that discretized the values of the numeric variable into equally

sized bins, i.e., ranges of variable values. The default number of bins equals the squared root of the number of observations of the variable (in our case, the length of the time series) Meyer et al. [21].

Equal frequencies discretization method partitions the range of the given random variable X into ranges with the equal number of data points. Thus, it results in bins different sizes [23]. The default number of the bins again equals the squared root of the time-series length.

The literature overview reveals that RT approach often uses mutual information as an association [1, 8, 14, 4, 15, 19]. In that context, Emmert-Streib et al. [4] consider mutual information as a base for each RN-approach variant.

Mutual information is capable of discovering non-linear inter-dependencies, but, as other simple association measures, is not able to infer the direction of influence and produces undirected network.

Simple distance measures

Simple distance measures assess the gene regulatory interactions by calculation of the distance between expression time-series. In general, they operate over vectors of values and they share same approach or ground norm. Three different measures has been included in the study: L^{10} Norm, Euclidean distance and Manhattan distance.

The ground norm that appears as a basis for all three distance measures is, so called L^s Norm:

$$\delta_L(x, y) = (\sum_{k=1}^{n} |x_k - y_k|^s)^{(1/s)}, \tag{9}$$

where s is the norm that represents the dimension of the space in which vectors x and y are compared [24]. We consider L^{10} **Norm** (with $s = 10$) as a distance measure, among following two special cases.

Euclidean distance is a special case of the L^s Norm, with $s = 2$:

$$\delta_{ED}(x, y) = \sqrt{\sum_{k=1}^{n} (x_k - y_k)^2}. \tag{10}$$

Furthermore, **Manhattan distance** represents the distance as a shortest path between two points of vectors x and y, placed in a rectangular grid. It is analogous to the L^1 Norm:

$$\delta_{MD}(x, y) = \sum_{k=1}^{n} |x_k - y_k|. \tag{11}$$

The simple distance measures described above are limited to detecting linear inter-dependencies between two time-series. The determination of gene regulatory interactions is based on a raw vector, a time-component of which is dismissed. They have been applied in the context of RN from the early stage of this approach [10] and since then regularly appears as part of RN and review studies [4, 1, 8, 6].

Dynamic time warping

Dynamic time warping (DTW) measure relies on finding an optimal distance mapping between the two time series. It tries to capture differences between time series with regard to the time and speed. Originally developed in the context of speech recognition [25], it found its use in a wide range of applications in the domains of medicine and bioinformatics [26, 27].

DTW algorithm is performed in two steps. First, local distances are calculated for all pair of points in the time series, using simple distance measure, e.g., Euclidean distance, that has been used in our study. Then, the pairs of time points are aligned so that a minimal path is found where each point is included at least once and the sum over the lengths of all links is minimal. DTW allows specifying constraints of the alignment paths, as well as different point-wise local distance. In this study, we consider Euclidean distance as a local distance and three different constraints of alignment paths or step patterns: *symmetric1*, *symmetric2* and *asymmetric*. For full reference and description of these step patterns can be found in the documentation of "*dtw*" R-package [28], which has been to implement DTW for our comparative study.

Symbolic measures

Simple qualitative distance is based on a qualitative comparison of the shape or trends of the time-series. In the essence, simple qualitative distance qualitatively estimates the change in trends among each pairs of time-points in time-series x and compare the trends against a second time-series y:

$$\delta_{QD}(x, y) = \sum_{k=1}^{n-1} \sum_{j=k+1}^{n} \frac{2 \cdot Diff(q(x_k, x_j), q(y_k, y_j))}{n \cdot (n-1)}, \tag{12}$$

with $Diff(q_1, q_2)$ a function that defines the difference between different qualitative changes, which are defined as *increase* if $x_k < x_j$, *no-change* if $x_k \approx x_j$, and *decrease* if $x_k > x_j$.

Simple qualitative distance has been proposed by Todorovski et al. [29] and used in the context of gene regulatory networks in Slavkov et al. [30], where the distance has been applied for performing clustering over gene expression time-series. The simple qualitative distance can be computed for very short time-series, without decreasing the quality of the estimate. Furthermore, it captures the non-linear inter-dependencies between gene expressions.

Symbolic similarity measures operate on a symbolic dynamics in order to uncover patterns of interaction. This similarity measures have been applied in the domain of bioinformatics in Wessel et al. [31]. Later, Hempel et al. [8] did exhaustive research and application of symbolic similarity measures in the domain of RN.

In order, symbolic similarity measures to be applicable on expression time-series, they need to be transformed into symbol sequences, as described in Marwan et al. [32]. The complete guidance of performing this step is also presented in the work of [8].

In this study, we include three symbolic similarity measures, one as a base measure and other two as its variants: **symbol sequence similarity**, **mutual information over symbol vectors**, and **linear combination of both**.

Important disadvantage of these measures is the computation time if the time-series are longer. Hence, possible constraints are applicable with regard to the length of symbol sequences α, which in our case has been determining as follows:

$$\alpha = \begin{cases} [1/n], & \text{if } n < 10 \\ 5, & \text{otherwise} \end{cases} \tag{13}$$

where, n is the number of time-points in the time-series.

Worth mentioning is the fact that all distance measures aforementioned produce undirected network, hence symmetry-breaking techniques are required in order to infer directed links between genes in a GRN.

Following subsection, explains the second component of the RN, scoring schemes.

Scoring schemes

Scoring schemes are considered in order to control the resulting association scores, henceforth *weights*. There are various scoring schemes that can go along with above mentioned association measures, but here we limit the set to the one proposed by Hempel et al. [8]: reconstruction of accurate cellular networks, context likelihood of relatedness, maximum relevance/minimum redundancy network, and asymmetric weighting.

Accurate for the Reconstruction of Accurate Cellular NEtworks (ARACNE) is based on data processing inequality (DPI) [33] paradigm and states that post-processing cannot improve already acquired knowledge. In essence, it performs testing of all gene-triplets (consisted of three genes that have mutual information among them greater than some threshold I_0), such that for each triplet the edge corresponding to the lowest mutual information I_1 among their mutual information, is eliminated from the adjacency matrix:

$$A_{i'j'} = A_{j'i'} = \begin{cases} 0, & \text{if } I_{i'j'} \geq I_2(1-\epsilon) \\ 1, & \text{otherwise} \end{cases} \tag{14}$$

where $I_{i'j'} = argmin\{I_{ij}, I_{jk}, I_{ik}\}$ is the lowest mutual information among them within a gene-triplet, I_2 is the second lowest mutual information, and factor ϵ value is between 0 and 1 and represents tolerance parameter [34, 35].

Moreover, the ARACNE removes all edges satisfying $I_{i'j'} < \tau$, where τ is predefined threshold [8].

ARACNE is capable of controlling the regulation of a gene over another gene, by modifying the initially inferred network on the basis of mutual information. However, the resulting network is still undirected.

Context Likelihood of Relatedness (CLR) [36] is considered as an extension to the basic relevance network approach proposed by Butte and Kohane [12]. As opposite to ARACNE, CLR performs pair-wise comparison of mutual information. At second step, it estimates the statistical likelihood of a mutual information for given pair of genes (I_{kj}), by comparing to the marginal (gene-specific) distribution. Thus, two scores are derived, one for gene k and one for gene j. By making the normality assumption about these distributions, corresponding scores z_k and z_j are calculated as follows:

$$z_k = max(0, \frac{1}{\sigma_k} - \frac{\bar{I_k}}{I_{kj} \cdot \sigma_k}), \tag{15}$$

$$z_j = max(0, \frac{1}{\sigma_j} - \frac{\bar{I_j}}{I_{kj} \cdot \sigma_j}), \tag{16}$$

where, final score for a pair of gene is obtained as follows:

$$z_{kj} = \sqrt{z_k^2 + z_j^2}. \tag{17}$$

As opposite to ARACNE, CLR does not relies on a global thresholds, but on local computed background of each gene. The outcome of CLR is undirected network.

Maximum Relevance/minimum redundancy NETwork (MRNET) is a supervised method that performs series of maximum relevance/minimum redundancy gene selection procedures [37]. Expression of a gene is considered as a target, while the rest genes as description variables in the supervised procedure. Hence, $y = x_k$, with $V = x \setminus x_k$ as description space. Given the set M of selected variables and pairwise weights w_{kj}, the procedure updates M by choosing the variable:

$$x_j^{MRMR} = argmax(s_j), x_j \in V \setminus M, \tag{18}$$

and maximizes the score:

$$s_j = u_j - r_j, \tag{19}$$

where $r_j = \frac{1}{|M|}\sum_{x_i \in M} w_{ji}$ is the redundancy term, while $u_j = w_{jk}$ is the relevance term.

The above procedure tries to differentiate between direct and indirect links. Hence, direct links is assigned with higher importance (relevance), while indirect links with lower importance (redundancy). Thus, the final matrix is calculated with:

$$f_{kj} = \frac{max[(w_{jk} - r_j), (w_{kj} - r_k)]}{w_{kj}}, \tag{20}$$

MRNET assigns weights w based on simple mutual information and employee additional parameter τ that is used for eliminating edges with unimportant score. The algorithm is not capable of inferring directionality of a GRN.

Asymmetric WEighting (AWE) is an asymmetric weighting schema based on topological aspects of complete set of pairwise weights obtained from particular association method [8]. Given in a matrix, AWE assumes columns as genes that are regulated by other genes, and rows as genes that regulates other genes. The asymmetric weight is then calculated by dividing each row by the sum of the corresponding column scores:

$$f_{ij} = (\sum_{k=1}^{m} w_{kj})^{-1}. \tag{21}$$

Thus, the probability that j^{th} gene is regulated sum up to a unit:

$$\sum_{k=1}^{m} c_{kj} = \sum_{k=1}^{m} (w_{kj} \cdot f_{kj}) = 1. \tag{22}$$

The obtained score indicates how likely a gene is regulating another one, which depends not only on the strength of interactions, but also on their amount. This scoring schema is capable of inducing directionality in a GNR. First application of the schema in the domain of RN is given in Hempel et al. [8].

Time shifting

Time shifting is a method for inferring the direction of an undirected link between two nodes from data. The main idea is to shift one of the time series, e.g., X in one direction and observe the change of the association $\mu(X, Y)$, using particular association measure μ. The change of the association measure with the time shift provide useful information that can be used to infer the direction of the influence, i.e., the direction of the network link. The complete procedure is described in Hempel et al. [8] and Yu and Parlitz [38].

We use the time method as a third, obligatory component of the RN approach variant in all cases when the scoring scheme results in an undirected network links. For the AWE scoring scheme, where result of the scoring scheme is a directed network, the application of time shifting method is optional. In that case, we have two alternative RN-approach variants: one with and one without applying the time shifting approach.

Material & Methods

In the comparative evaluation of the variants of the RN approach, we have considered all combinations of the association measures and scoring schemes with the time-shifting method applied where appropriate. There are 114 candidate combinations corresponding to 114 variants of the relevance network approach. Each of the 114 variants was applied to the 38 tasks of GRN inference and the performance of the network inference was measured by comparing the inferred network structure with the structure of the given network (in case of reconstructing known networks from simulated data) or with the structure of the best known network (in case of real measurements). One performance measure is area under the receiver-operator characteristics curve, the other two are two different areas under the precision-recall curve.

The goal of the comparative analysis is to identify the best performing variants of the RN approach and the properties thereof. We are especially interested in finding out what association measures and scoring schemes work best and what are the interactions between them that lead to the best performance. We also investigate the impact of the time-series length and network size on the best performing variants of the RN approach.

To identify the best performing methods for a given set of GRN inference tasks, we proceed as follows. First, for each performance measure and each task, we sort and rank the methods with respect to their decreasing performance on the task, so the top-performing method gets the rank of 1 and the worst-performing method the rank of 114. Furthermore, for each performance measure, we calculate the average ranks of the methods along the given set of tasks. Finally, we perform a Pareto analysis of the three-dimensional space of performance metrics to identify Pareto fronts of points corresponding to the best performing methods, i.e., methods with the lowest average ranks.

The continuation of this section provides further details on the experimental setup for performing the comparative analysis: we first introduce the tasks of GRN inference, then provide detailed description of the performance metrics used and conclude with brief overview of the implementation details.

Data description

The comparative has been conducted using simulated and real time-series data. In particular, the data or the simulation model used for obtaining data are based on two microorganisms: Escherichia coli (henceforth *E.coli*) and Saccharomyces cerevisiae (*Yeast*). We use data sets from three previously published studies on GRN inference.

The first data source is Hempel et al. [8], where datasets were generated by the tool SynTReN [39] on the basis of the well-known gene regulatory networks in E.coli and Yeast. We consider sub-networks of 100, 150 and 200 genes, characterized by 121, 202 and 303 existing links with an average node degree of 2.42, 2.46 and 3.03, respectively. In order to guarantee consistency between sub-networks and expression data, SynTReN generates different expression data for each selected sub-network. Additionally, three level of noise have been considered: 0.0 (deterministic — without noise), 0.1 and 0.5. These values represent the σ parameter of the log-normal distribution $\sim logX(0, \sigma)$, according to which the noise is generated by SynTReN. For each configuration, 6 technical replicates of 10 time points have been generated and the expression data associated with each gene is obtained as the average over the replicates. This is necessary to cope with the non-deterministic nature of the SynTReN data generation algorithm (the 18 data sets labels starting with *E1* and *Y1* in Table 1).

Another source of data is *DREAM5 challenge* [40, 1]: from the five networks available, We consider two, *Network3* and *Network4*, based on Affymetrix gene expression data of E.coli and Yeast, respectively, taken from the Gene Expression Omnibus (GEO) database [41] and collected under a wide range of biological conditions. For each network, a set of experiments has been performed over genes, out of which we consider two experiments per network, and create four tasks (data sets). Network3 contains 4511 genes and 2066 known (existing) links with density of 1.1^{-3}, while Network4 has 5950 genes, 3940 known links and density of 3.8^{-4}. Time series lengths vary from 5 to 48 time points. The four rows in Table 1 with data set labels containing *E2* and *Y2* provide summary description of the four tasks corresponding to the second data source.

Organism	Data set	Coverage	Size	Length	Noise
E. coli	*E1_1*	100	100	10	0.0
E. coli	*E1_2*	100	150	10	0.0
E. coli	*E1_3*	100	200	10	0.0
E. coli	*E1_4*	100	100	10	0.1
E. coli	*E1_5*	100	150	10	0.1
E. coli	*E1_6*	100	200	10	0.1
E. coli	*E1_7*	100	100	10	0.5
E. coli	*E1_8*	100	150	10	0.5
E. coli	*E1_9*	100	200	10	0.5
E. coli	*E2_1*	100	4511	6	0
E. coli	*E2_2*	100	4511	5	0
Yeast	*Y1_1*	100	100	10	0.0
Yeast	*Y1_2*	100	150	10	0.0
Yeast	*Y1_3*	100	200	10	0.0
Yeast	*Y1_4*	100	100	10	0.1
Yeast	*Y1_5*	100	150	10	0.1
Yeast	*Y1_6*	100	200	10	0.1
Yeast	*Y1_7*	100	100	10	0.5
Yeast	*Y1_8*	100	150	10	0.5
Yeast	*Y1_9*	100	200	10	0.5
Yeast	*Y2_1*	100	5950	5	0
Yeast	*Y2_2*	100	5950	48	0
Yeast	*Y3_1_2*	100	42	5	0
Yeast	*Y3_1_3*	100	42	5	0
Yeast	*Y3_1_11*	97.6	41	8	0
Yeast	*Y3_1_13*	100	42	5	0
Yeast	*Y3_1_14*	100	42	5	0
Yeast	*Y3_1_15*	100	42	10	0
Yeast	*Y3_2_2*	96	72	5	0
Yeast	*Y3_2_14*	96	72	5	0
Yeast	*Y3_3_2*	96.3	289	5	0
Yeast	*Y3_3_3*	95.7	287	5	0
Yeast	*Y3_3_10*	95.3	286	7	0
Yeast	*Y3_3_13*	96.7	290	5	0
Yeast	*Y3_3_14*	95.7	287	5	0
Yeast	*Y3_4_2*	96.3	181	5	0
Yeast	*Y3_4_3*	95.7	180	5	0
Yeast	*Y3_4_13*	95.2	179	5	0

Table 1. Properties (columns) of the data sets for 38 GRN inference tasks (rows): organism, data-set label, percentage of network nodes covered in the data set, number of network nodes and time-series length.

Third data source provides real measurements, collected as a part of the study conducted by Gasch et al. [42], which aims to explore changes in expression levels of Yeast genes under diverse environmental stresses, such as heat shock, diauxic shift, diamide treatment, and amino acid starvation. The measurements have been taken at different time points, using microarrays. One network has been observed, where four different independent sub-networks were identified, which are considered

as separate networks within this study, with size of 42, 75, 300 and 300 nodes. For the observed sub-networks 13 different stresses have been monitored, thus 52 datasets are available. Since some data sets provide limited coverage of the network nodes, we consider only 20 data sets that have network coverage greater than 95%. The time-series observed are of different lengths, from 5 to 11 time points (the last 16 rows in Table 1).

Performance metrics

To evaluate the performance of the inference method on a given task, we perform a matching between the structure (links) of the given/known GRN (true network) and the structure (links) of the inferred GRN (inferred network). Since the output of the inference method is a network connectivity matrix containing numeric link weights, we can perform the matching after setting the threshold value that would decide upon the presence and absence of links. To this end, we set aside metrics that require prior assumptions, i.e. performance metrics that require predefined or default discrimination threshold. Instead, we follow the standard framework for evaluating network inference and employ *thresholding metrics*, which consider variability of the discrimination threshold and avoid setting it to default value. Thus, methods are evaluated with regard to the complete set of possible thresholds, which results in analysis of performance space. For this purpose, two different spaces have been applied: *Receiver operating characteristic curve* and *Precision-Recall curve* space.

Both spaces are defined over quantities derived from a confusion matrix. A confusion matrix [43, 44] is a matrix that consists of four basic quantities that represent the correctness of link predictions: number of correctly recognized true network links (true positives, *TP*), number of correctly recognized absent links in the true network (true negatives, *TN*), and links that either have been incorrectly predicted to be present (false positives - *FP*) or true network links that were predicted as absent (false negatives, *FN*). These basic quantities are further combined in order to express more specific performance perspectives. In the following formulas, we are going to use P to denote the number of true network links and N to denote the number of absent links in the true network.

Receiver operating characteristic curve (*ROC curve*) is a two-dimensional space that illustrates the performance of a binary classifier as its discrimination threshold is varied [45]. Its dimensions correspond to two performance metrics of true positive rate, *TPR* (Eq. 23) and false positive rate, *FPR* (Eq. 24) for various threshold settings. It depicts the relative trade-offs between true positives *TP* and false positives *FP*, which are interpreted as *benefit* and *cost*, respectively.

$$\mathrm{TPR} = \frac{\mathrm{TP}}{\mathrm{P}} = \frac{\mathrm{TP}}{\mathrm{TP} + \mathrm{FN}} \tag{23}$$

$$\mathrm{FPR} = \frac{\mathrm{FP}}{\mathrm{N}} = \frac{\mathrm{FP}}{\mathrm{FP} + \mathrm{TN}} \tag{24}$$

Since, the ROC curve is two-dimensional, various summary statistics can be derived. Most commonly used is *Area Under the Curve* (AUC) that quantifies the area that is found below the curve, which is also considered in the study for the comparative evaluation. AUC is calculated by integrating the area under the curve, and express it as a single quantity (area in two-dimensional space).

The ROC space is a unit two-dimensional space with total area of 1. Thus, it can be plotted on a two-dimensional plot with both axes ranging from 0 to 1. Furthermore, the ROC curve is characterized with its monotonicity, i.e. monotonic incremental curve, which to certain extend, guarantees that by considering the curve, optimal threshold can be found. The ROC curve or analysis overall, is suitable for comparison of a classifier with a default classifier (random selection), which is represented in the space as a diagonal line from (0, 0) to (1, 1).

However, the ROC analysis has disadvantages, as well. Mainly, it can be misinterpreted if the problem under consideration is characterized with imbalanced distribution of class values. This disadvantage can appear due to the fact that true negatives are considered as correct classification of examples, even though the problem deems for correct classification of positive examples (classification of minority class) only. Reconstruction of GRNs is such a problem, where we face large networks with very small number of existing links (minority class), and many non-existing links (majority class). Hence, correct classification of the former is much complex tasks, than correct classification of the latter. The ROC analysis dismiss the complexity of the classification tasks and represent the correct classification of minority class as equally important as correct classification of non-existing links.

The AUC quantity, on the other hand, has its own properties. Its values ranges from 0 to 1: values close to 1 represent better classifier, while values around 0.5 means that the classifier is nothing better than the default (random) classifier. Everything below 0.5 means that the evaluated classifier behaves worse than the default classifier. Disadvantages of the ROC curve reflect also to the AUC quantity. Namely, considering the problem of GNR reconstruction, we can end up with overall high AUC, increased mainly by the accurate classification of the majority class (correctly predicting non-existing links).

Precision-Recall curve (*PR curve*) is also a two-dimensional space that illustrates performance of a binary classifier as its discrimination threshold is varying [46, 47]. Commonly, it is referenced as a replacement for ROC curve in case of highly imbalanced class distribution [48]. The space is defined with two metrics derived from the confusion matrix: *recall* (Eq 25) and *precision* (Eq. 26).

$$\mathrm{recall} = \mathrm{TPR} = \frac{\mathrm{TP}}{\mathrm{P}} = \frac{\mathrm{TP}}{\mathrm{TP} + \mathrm{FN}} \tag{25}$$

$$\mathrm{precision} = \frac{\mathrm{TP}}{\mathrm{TP} + \mathrm{FP}} \tag{26}$$

Summary statistics can also be derived from PR curve and commonly used is the *Area Under the PR Curve* (AUPRC). In this analysis, we used two different portions of the area under the curve: partial *AUPRC-0.2* and total area *AUPRC*. Guided by the importance of discovering only true links, without expectation all of them to be discovered, we consider the 20% of the AUPRC that corresponds to the lower recall (up to 0.2), referred to as *AUPRC-0.2*. It means that we try to evaluation a classifier in accordance to the top scored predictions to be true links. So, if the classifier has high precision within this region (sub-space) then it is considered as good classifier and can assure that those links that are predicted with very high scores, are true links.

As opposite to ROC curve, PR curve is not monotonic curve and therefore, the performance can varying while varying the discrimination threshold. The curve is plotted into two-dimensional unit space, with total area of 1, and starts from the point (0, 1) and finish in the point (1, 0). There is no PR curve for a default (random) classifier, instead some agreements on how it could be plotted on a graph [48]. The improvement over ROC curve is the fact that the class imbalance does not

affect correct performance estimation. This is because *TN* are excluded from calculations, which is a property that breaks the monotonicity. The PR curve can be very sparse in terms of points when dealing with imbalanced data. Therefore, interpolation is recommended [48, 49]: to this end we employ the implementation by Brodersen et al. [50].

Similarly to *AUC*, *AUPRC* values are in the range from 0 to 1, while *AUPRC-0.2* values range from 0 to 0.2, where higher values indicate better performance. In the continuation of the paper, we will refer to the three performance measures introduced here as AUROC, AUPRC and rAUPRC (restricted AUPRC).

Ranking of methods

To rank the variants of the RN approach according to their performance on the 38 tasks, we first filter out the low-performing methods by testing the statistical significance of the difference between the measured method performance and the performance of a random classifier. In particular, we employ one-sample Student's t-test to check whether the average method performance on the GRN inference tasks is significantly larger than 0.5 (the expected AUROC and AUPRC performance of a random classifier) and 0.2 (for the rAUPRC performance measure). Methods that are not significantly better than a random classifier with respect to at least one of the three performance measures are extracted from further analysis.

In the next step, for each performance measure and each GRN inference tasks, we rank the RN-approach variants according to their performance on the particular task. Then, we average method ranks over all the tasks to obtain three mean rankings of the RN-approach variants with respect to the AUROC, AUPRC and rAUPRC measures. To obtain a joint ranking along the three performance measures, we employ the non-dominated sorting algorithm used in multi-objective decision theory [51]. We first embed the variants into a three-dimensional space, where each dimension correspond to a ranking of the network-relevance methods with respect to one of the performance measures. Each RN-approach variant corresponds to a single point in that space, where each coordinate equals the ranking of the method to a particular performance measure. Note that we normalize the method rankings on the $[0, 1]$ scale, using a simple linear transformation $(r_M - 1)/(N - 1)$, where r_M is the rank of the method M, while N denotes the number of all compared methods. Figure 1 depicts the projection of the three-dimensional space in two dimensions obtained using multidimensional scaling [52]. The red, green, blue labels and grey points in the graph correspond to the compared RN-approach variants.

To identify the top-ranked RN-approach variants, we search for a set of non-dominated points in the three-dimensional space, i.e., we identify the Pareto front of the non-dominated points in the space. The points in the Pareto front correspond to the methods that are the best performers according to at least one performance measure. After we assign the top ranks to these RN-approach variants, we remove the corresponding points from the three-dimensional space and iteratively continue to identify Pareto fronts in the reduced sets of points until all the methods are ranked.

Table 2 presents the joint ranking along the three performance measures obtained with the non-dominated sorting algorithm described above. For each Pareto front, we can calculate the hypervolume of the space dominated by the points on the front. The volume change indicates the magnitude of differences between rankings of the methods in two Pareto fronts. Figure 1 depicts the first three Pareto fronts (red, green and blue points) in the two-dimensional projection of the original three-dimensional space. They include the 13 top-performing

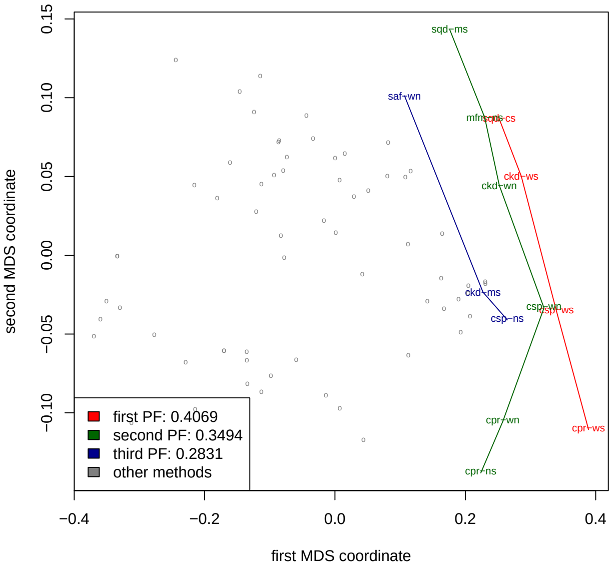

Figure 1. The first three Pareto fronts (PF) in the three-dimensional space of mean rankings of the variants of the RN approach with respect to the three performance measures of AUROC, AUPRC and rAUPRC. The rankings of the variants are averaged over all the network reconstruction tasks from Table 1. Each number in the legend represents the hypervolume dominated by the points in the corresponding Pareto front. The two-dimensional projection of the three-dimensional space was obtained using multidimensional scaling.

methods according to the three performance measures taken together simultaneously.

Availability of Data and Materials

We implemented all the components and the variants of the RN approach in the R software environment for statistical computing. We implemented most of the components using standard R functions, except for the association measure based on the dynamic time warping measure of distance between time series. To this end, we have used the implementation of the DTW in the R package *dtw* [28]. The source code of our implementation of the RN-approach variants in R are publicly available:

- Project name: RN-approach project
- Project home page: https://vkuzmanovski@bitbucket.org/vkuzmanovski/rn-approach.git
- Operating system(s): Platform independent
- Programming language: R
- Other requirements: None
- License: FreeBSD

To measure the performance metrics, we have used the functions implemented in the R package for evaluating the performance of classifiers *ROCR* [53]. For performing the Pareto analysis, we used the implementation of non-dominated sorting algorithm in the R package for multi-objective optimization *emoa* [54]. The source code of the R functions used to perform the comparative analysis of the RN-approach variants that allows for complete reconstruction of its results is also publicly available:

- Project name: RN-evaluation project
- Project home page: https://vkuzmanovski@bitbucket.org/vkuzmanovski/rn-evaluation.git

Table 2. The joint ranking of the RN-approach variants along the mean rankings with respect to the three performance measures of AUROC, AUPRC and rAUPRC. The method rankings are averaged over all the tasks from Table 1. Each row corresponds to a single Pareto front of non-dominated points: the column PF-I reports the Pareto front index, Dom-HV the volume of the space dominated by the Pareto front, and the last column includes the RN-approach variants corresponding to the Pareto-front points.

PF-I	Dom-HV	network-relevance methods
1	0.4069	cpr-ws csp-ws ckd-ws sqd-cs
2	0.3494	cpr-ns cpr-wn csp-wn ckd-wn mfm-ns sqd-ms
3	0.2831	csp-ns ckd-ms saf-wn
4	0.2606	ckd-ns mfm-ws saf-ns saw-ns saw-wn saw-ws
5	0.2362	mfm-as saf-ws
6	0.2306	mfm-wn mfm-ms smf-wn
7	0.2216	csp-ms mfo-ms smf-ws smw-wn
8	0.2110	smw-ns smw-ws saw-cs
9	0.1688	mfo-as mfs-ms smf-ns
10	0.1562	mfo-ns mwo-ms smf-cs saf-cs
11	0.1386	mwo-ns mwo-ws smw-cs
12	0.1309	cpr-cs mwo-wn mfs-ns sss-cs
13	0.1143	csp-cs mfo-wn mfo-ws mfs-as mfs-wn mfs-ws smw-ms
14	0.1062	dmn-as dec-as ws1-as mfm-cs smf-ms saw-ms
15	0.0936	d10-as mwo-cs sqd-wn sqd-ws saf-ms
16	0.0808	cpr-ms ckd-cs mfo-cs sqd-ns
17	0.0619	mwo-as mfs-cs sqd-as sss-ms
18	0.0315	ckd-as sss-wn sss-ws
19	0.0268	csp-as sss-ns sss-as

- Operating system(s): Platform independent
- Programming language: R
- Other requirements: None
- License: FreeBSD

Results

In the comparative analysis of the method performance, we focus on top-performing methods in the first three Pareto fronts identified with the non-dominated sorting algorithm. For each RN-approach variant in these Pareto fronts, we analyze its composition in terms of the association measure and the scoring function employed. We proceed with the analysis as follows. First, we identify the overall top-performing methods on the 38 GRN inference tasks from Table 1. Next, we analyze the impact of two properties of the GRN inference tasks on the RN approach performance: time-series length and network size.

All GRN inference tasks

The comparison of performance with respect to the all GRN inference tasks identifies the correlation-based and symbolic association measures (Figure 2, left) as the top performing ones. Correlation-based association measures appear to be among best performers in all three Pareto fronts, dominating by total number of RN-approach variants. In total, 9 variants using correlation coefficients are found in the leading Pareto fronts (out of 13 variants dominating the performance space). In the first Pareto front, correlation-based measures are represented in three variants, followed by four appearances in the second Pareto front, and two in the third. The symbolic association measures represent the second most-frequent group in the RN-approach variants that dominate the performance space. They appear in all three Pareto fronts: once in each of them. Finally, the two dominant association measures are followed by a group of a single association measure based on mutual information in the second Pareto front.

In contrast with the clear differences in performance among the association measures, the scoring schemes can not be so clearly differentiated (Figure 2, right). Namely, all scoring schemes except ARACNE appear across top three dominant Pareto fronts. The AWE scoring scheme is the most frequent one: it appears in seven RN-approach variants in all three Pareto fronts. In the first Pareto front, AWE is a component of three variants: in all three variants it is used without the time-shifting method. In the other two Pareto fronts, the AWE scoring scheme is combined with the time-shifting method. The other scoring schemes are identified to be equally frequent among the top-performing RN-approach variants in the three Pareto fronts.

The impact of the time-series length

To investigate the impact of time-series length (l) on the performance of the RN-approach variants, we clustered the 38 data sets into three groups of tasks with short ($l \leq 5$), medium-length ($5 < l < 10$), and long ($l \geq 10$) time series.

Figure 3A provides the overview of the seven top-performing methods on the data sets with short time series. Two group of association measures appear: symbolic and mutual information (Figure 3A, left). Four RN-approach variants that include symbolic association measures are found in top two Pareto fronts. Association measures based on mutual information appear in three variants distributed evenly among the three Pareto fronts. Scoring scheme analysis (Figure 3A, right) do not emphasize dominance of any particular scoring scheme: except MRNET, all are among top-performing RN variants.

The performance of the methods on time series with medium length is quite different from the performance on the short time series (Figure 3B). Again, we observe significant dominance of symbolic association measures among the top-ranked method compositions (five out of six method compositions, Figure 3B, left). Additionally, a single variant with a correlation-based association measure appears in the second front. In the case of scoring schemes, distribution of schemes stays similar to one in short time-series settings, except for ARACNE, which is not identified among most dominant method compositions, and MRNET that appears to be single dominant scheme in the third front (Figure 3B, right).

The comparison of RN-approach variants on tasks with long time series, presented in Figure 3C leads to different results.

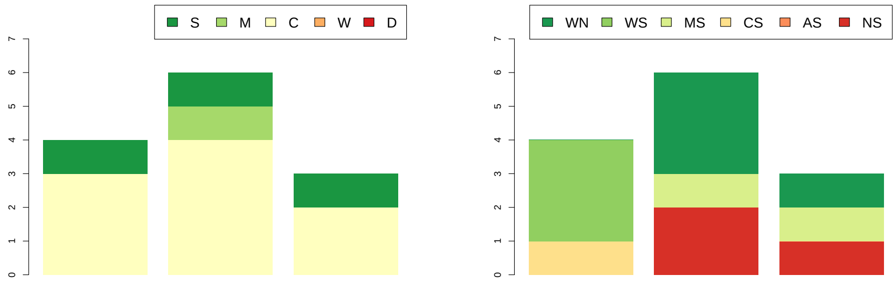

Figure 2. The association measures (left-hand side) and scoring schemes (right-hand side) used by the 13 top-ranked variants of the RN approach from the first three Pareto fronts in the three-dimensional space of AUROC-AURPC-rAUPRC mean rankings. The rankings are averaged on the 38 GRN inference tasks from Table 1. Legend on the left-hand side: S denotes the class of symbolic and qualitative association measures, M — association measures based on mutual information, C — correlation-based, W — dynamic time warping, and D — distance-based association measures. Legend on the right-hand side: WN denotes the AWE scoring scheme without time shifting, WS, MS, CS and AS — the AWE, MRNET, CLR and ARACNE with time shifting, and NS — the time-shifting method without a scoring scheme.

Among the 11 top-ranked RN variants, the correlation-based association measures prevail: they participate in five variants distributed among all three Pareto fronts (Figure 3C, left). Symbolic association measures appear in four variants, two of them being in the third Pareto front. Two variants that use mutual information as association measure are among the top-ranked ones in the first two Pareto fronts. Differences with results on shorter time series are visible among scoring schemes, as well (Figure 3C, right). The AWE scoring scheme appears in nine variants among the 11 top performers: 4 times in combination with time shifting, and 5 times without.

The impact of the network size

We consider the number of network nodes or genes (n) to be a measure of the network size. When analyzing its impact on the performance of the RN approach, we cluster the GRN inference tasks into three groups of tasks with small ($n \leq 100$), medium-size ($100 < n \leq 1000$), and large ($n > 1000$) networks.

Figure 4 gives top-performing variants of the RN approach for tasks with different network sizes. The top-performing association measures for small networks are correlation (seven out of 13), mutual information (four out of 13) and symbolic/qualitative methods (two out of 13, see Figure 4A, left). Correlation-based association measures prevail the first two Pareto fronts, mutual information are included in method compositions in the second and third Pareto fronts. The distribution of scoring schemes among the 13 top-performing variants emphasize AWE (with and without time shifting) prevailing (in 7 out of 13 variants), which is followed by no scoring scheme, CLR and MRNET (Figure 4A, right).

Similar situation is observed across medium networks for both association measures and scoring schemes (Figure 4B). Among the 11 top-performing variants of the RN approach, eight employ association measures based on correlation, two employ symbolic methods and one in the third Pareto front employs mutual information (Figure 4B, left). In regard with the scoring schemes, AWE is even more prevailing as a scoring scheme used by the top-performing method compositions, while MRNET is no longer found among the top performers (Figure 4B, right).

Finally, the results for the large networks show strikingly resolute picture shown in Figure 4C. All four top-performing variants of the RN approach is a combination of a symbolic association measure with the MRNET scoring scheme.

Discussion

The goal of the discussion presented here is to address the main issue raised in the introduction, i.e., the issue of "What Works Where?" or *What would be the reasonable choice of association measure and scoring scheme in the RN approach for a given task of GRN inference*? The first series of experiments on all 38 tasks provides a relatively simple answer: the RN-approach variants based on correlation-based association measure and AWE scoring scheme perform best. Furthermore, the results show that the AWE scoring scheme works equally well with or without time-shifting method for inferring the link directions.

Note that both correlation-based measures and the AWE scoring schemes work over data samples and vectors. This fact might indicate that the particular method combination would work well not only for time-series (temporal component is mostly ignored by these RN-approach variants), but also for steady-state data. Furthermore, correlation-based measures perform well with other scoring schemes, except for the ARACNE. Based on the observation that correlation-based methods in combination with some scoring scheme get improved overall, leads to a conclusion that they can perform well with time-shifting only, but performance improvements can be gained by selecting an appropriate scoring scheme.

Symbolic association measures have been identified as second best performing group of measures that are frequently present among the top-performing RN-approach variants. In contrast with the correlation-based measures, they operate on temporal data only, and are therefore useful only in the context of time-series data. Also, symbolic measures appear to perform well only in combination with AWE scoring scheme.

Overall, correlation-based association measures show robustness with regards to the selection of a scoring scheme, while AWE scoring scheme improves performance in general, without limiting the choice of an association measure.

The results of the analysis of method performance on data sets with varying time-series length reveal further "What Works Where?" insights. For short time series, symbolic and mutual information association measures lead to top-performing variants of the RN approach. Symbolic measures behave robust and work well in combination with all scoring schemes, except the one that apply time-shifting only. This leads to a conclusion that symbolic association measures are robust in general and give more flexibility in choosing a scoring scheme, but need to be corrected by a scoring scheme prior to inferring the links directions. Unlike the symbolic association measures, the ones based on mutual information do

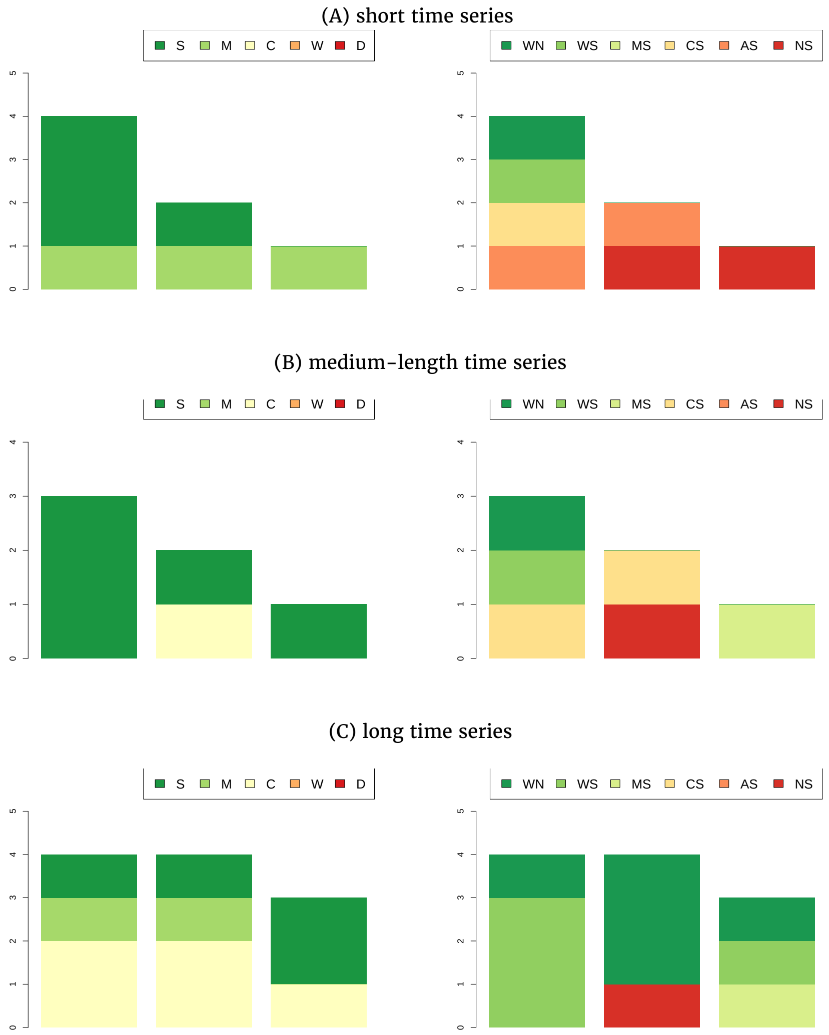

Figure 3. The association measures (left-hand side) and scoring schemes (right-hand side) used in the thirteen top-ranked variants of the RN approach from the first three Pareto fronts in the three-dimensional space of AUROC-AURPC-rAUPRC mean rankings. The rankings are averaged on the network reconstruction tasks involving short (A), medium-length (B) and long (C) time series.

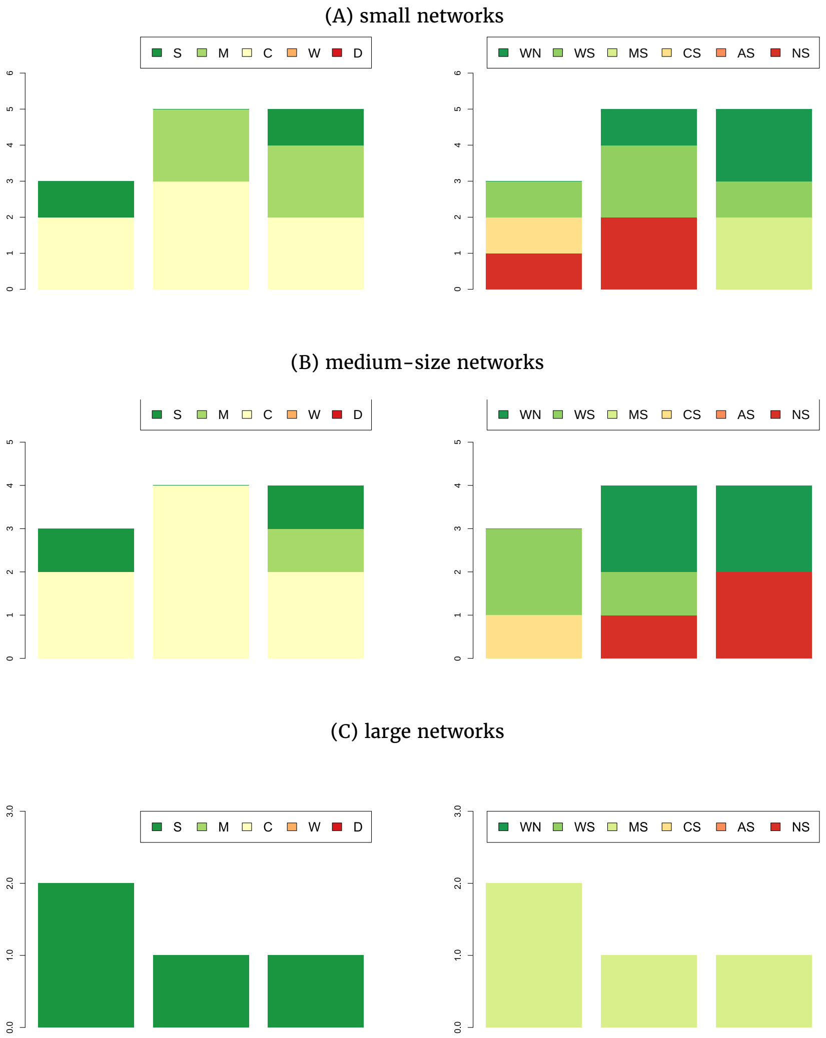

Figure 4. The association measures (left-hand side) and scoring schemes (right-hand side) used in the top-ranked variants of the RN approach from the first three Pareto fronts in the three-dimensional space of AUROC-AURPC-rAUPRC mean rankings. The rankings are averaged on the network reconstruction tasks involving small (A), medium-size (B) and large (C) networks.

not show robustness with respect to the selection of a scoring schemes. They perform well only if combined with ARACNE scoring scheme or time-shifting method is applied without any scoring scheme. Overall, the results also show that for short time series, the selection of the scoring scheme depend on the choice of the association measure.

Among medium-length time series, symbolic association measures fortified the dominance over the rest measures, in combination with variety of scoring schemes. Hence, the robustness of these measures persists and still gives flexibility in choosing a scoring scheme. The only opponent that appears among dominant measures is correlation-based measures in combination with time-shifting only.

The dominance of the correlation-based measures increases in the setting of long time series. Namely, they have been identified in most of the tasks as part of the best performing method compositions. However, their robustness about combination with scoring schemes is limited mostly to AWE, while time-shifting only and MRNET are observed in one case only. Following dominant association measures are symbolic measures with strong limitation in choosing a scoring scheme, i.e. AWE with or without time-shifting. Similarly, mutual information measures appear to be among dominant compositions in two cases, in combination with AWE.

Conclusively, in setting of short time series, symbolic association measures are recommended robust solution, while they become more dependent on limited set of scoring schemes as the length of the time series increases. This is a result of their properties to examine the associations exhaustively throughout the time point's space. Therefore, shorter time series are they are capable of complete search of the space, which is not a case in longer time series, where they are constrained due to the computational complexity. As opposite, correlation-based measures can be recommended as robust solution for long time series, since they are not constrained to retrieve knowledge from larger amount of data.

The comparison analysis of method performance applied over different sizes of a network shows more consistent results over different settings. Namely, correlation-based association measures outperform other measures over different sizes.

In case of small networks, correlation-based group of measures perform well in combination with all scoring schemes, except ARACNE. Similarly as in the general case, ARACNE performs aggressive cutoffs of inferred links without considering the difference between estimated associations of inferred links. However, from the observations we can conclude that correlation-based association measures are most robust solution and give flexibility in choosing a scoring scheme and construction of customized RN approach.

Following dominant group of association measures are symbolic and mutual information that perform well only in combination with AWE scoring scheme. Therefore, on the side of scoring schemes, AWE is robust perforer independently of the choice of an association measure.

Same observations are drawn for the settings of medium sized networks, where the only difference is in the set of scoring schemes, i.e. MRNET does not appear among best performing schemes. Given observation, however, cannot be confirmed in the case of large networks due to lack of sustain number of network reconstruction tasks. Therefore, the discussion of this setting is dismissed.

Finally, worth mentioning is the observation that distance-based association measures have not been identified among best performing association measures in none of the settings considered. Thus, they are excluded from the list of recommended group of association measures worth considering for tasks of GRN inference.

Conclusion

The comparative analysis presented in this paper is based on an extensive empirical evaluation of the performance of 114 variants of the general relevance network approach on 38 tasks of inferring gene regulatory networks from time-series data. The 114 RN-approach variants are based on 6 general classes of association measures (with a variety of parameter settings) and 6 scoring schemes, some of which are accompanied by a time-shifting method for inference of the directions of network links. The performance of the RN-approach variants is measured using three different performance metrics widely used in other studies on inferring gene regulations network from data.

The main contribution of this paper is the general framework for comparative evaluation of the numerous variants of the general relevance network approach to inference of gene regulation networks. The proposed framework is flexible and modular: one can easily extend it along any dimension of comparison, such as adding new association measures, scoring schemes, performance metrics or network inference tasks. The publicly available source code of the implemented framework allows for simple implementation of these extensions as well as reproducing the results presented in this study.

The main motivation for the evaluation performed in this paper is answering the question "What works where?". The answer provides important guidance for applying the general relevance network approach in a particular situation in terms of selecting an appropriate combination of an association measure and a scoring scheme that would lead to reasonably good performance on a given data set. The results of the comparative analysis lead to the following list of recommendations for configuring the relevance network approach:

- In general, the safest combination is the correlation-based association measure with the Asymmetric WEighting (AWE) scoring scheme.
- The association measures based on simple distances and dynamic time warping never lead to a top-performing variant of the RN approach.
- For short and medium-length time series (consisting of less than ten time points), the general class of symbolic association measures and the qualitative distance measure in particular leads to best performing variants of the RN approach; they can be combined with an arbitrary scoring scheme.
- For long time series (consisting of at least 10 time points), the general recommendation for combining a correlation-based association measure with the AWE scoring scheme applies.
- For large networks with more than 1000 nodes, symbolic association measures (combined with the AWE scoring scheme) gain an edge over the correlation-based ones.

While this set of recommendations provides clear guidance for selecting an appropriate variant of the relevance network approach, further experiments are necessary to strengthen the generality of the results. This is especially true for the results on the impact of network size: too few large-size networks are included in the current set of inference tasks. In further work, one would extend the set of inference tasks with ones that involves networks with a large number of nodes.

Another limitation of our study is its current focus on time-series data: the generality of the recommendation rules obtained here should be tested on the tasks of network inference from steady-state data. Another source of input for the relevance network approach, not considered in this study, can come in the form of expert knowledge about the presence or absence of certain links in the network. Note that none of these limitations should represent an obstacle for applying the pro-

posed framework for empirical evaluation on these extended tasks, since the framework is flexible enough to include them in the comparative analysis.

Acknowledgment

The authors acknowledge the financial support of the Slovenian Research Agency (research core funding No. P2-0103, No. P5-0093 and project No. N2-0056 Machine Learning for Systems Sciences) and the European Commission (grant ICT-2013-612944 MAESTRA).

References

1. Marbach D, Costello JC, Küffner R, Vega NM, Prill RJ, Camacho DM, et al. Wisdom of crowds for robust gene network inference. Nature methods 2012;9(8):796–804.
2. Stolovitzky G, Monroe D, Califano A. Dialogue on Reverse-Engineering Assessment and Methods. Annals of the New York Academy of Sciences 2007;1115(1):1–22.
3. Penfold CA, Wild DL. How to infer gene networks from expression profiles, revisited. Interface focus 2011;1(6):857–870.
4. Emmert-Streib F, Glazko G, De Matos Simoes R, et al. Statistical inference and reverse engineering of gene regulatory networks from observational expression data. Frontiers in genetics 2012;3:8.
5. De Jong H. Modeling and simulation of genetic regulatory systems: a literature review. Journal of computational biology 2002;9(1):67–103.
6. Markowetz F, Spang R. Inferring cellular networks–a review. BMC bioinformatics 2007;8(6):S5.
7. Hecker M, Lambeck S, Toepfer S, Van Someren E, Guthke R. Gene regulatory network inference: data integration in dynamic models — a review. Biosystems 2009;96(1):86–103.
8. Hempel S, Koseska A, Nikoloski Z, Kurths J. Unraveling gene regulatory networks from time-resolved gene expression data–a measures comparison study. BMC bioinformatics 2011;12(1):292.
9. Werhli AV, Grzegorczyk M, Husmeier D. Comparative evaluation of reverse engineering gene regulatory networks with relevance networks, graphical gaussian models and bayesian networks. Bioinformatics 2006;22(20):2523–2531.
10. Rays M, Chen Y, Su YA. Use of a cDNA microarray to analyse gene expression patterns in human cancer. Nature genetics 1996;14.
11. Eisen MB, Spellman PT, Brown PO, Botstein D. Cluster analysis and display of genome-wide expression patterns. Proceedings of the National Academy of Sciences 1998;95(25):14863–14868.
12. Butte AJ, Kohane IS. Mutual information relevance networks: functional genomic clustering using pairwise entropy measurements. In: Pac Symp Biocomput, vol. 5; 2000. p. 26.
13. Ceci M, Pio G, Kuzmanovski V, Džeroski S. Semi-supervised multi-view learning for gene network reconstruction. PloS one 2015;10(12):e0144031.
14. Hempel S, Koseska A, Nikoloski Z. Data-driven reconstruction of directed networks. The European Physical Journal B 2013;86(6):250.
15. Zhang B, Horvath S, et al. A general framework for weighted gene co-expression network analysis. Statistical applications in genetics and molecular biology 2005;4(1):1128.
16. Horvath S, Dong J. Geometric interpretation of gene coexpression network analysis. PLoS comput biol 2008;4(8):e1000117.
17. Kendall MG. A new measure of Rank correlation. Biometrika 1938;30(1-2):81.
18. de Matos Simoes R, Emmert-Streib F. Influence of Statistical Estimators of Mutual Information and Data Heterogeneity on the Inference of Gene Regulatory Networks. PLOS ONE 2011 12;6(12):1–14.
19. Soranzo N, Bianconi G, Altafini C. Comparing association network algorithms for reverse engineering of large-scale gene regulatory networks: synthetic versus real data. Bioinformatics 2007;23(13):1640.
20. Paninski L. Estimation of entropy and mutual information. Neural computation 2003;15(6):1191–1253.
21. Meyer PE, Lafitte F, Bontempi G. minet: AR/Bioconductor package for inferring large transcriptional networks using mutual information. BMC bioinformatics 2008;9(1):461.
22. Schäfer J, Strimmer K, et al. A shrinkage approach to large-scale covariance matrix estimation and implications for functional genomics. Statistical applications in genetics and molecular biology 2005;4(1):32.
23. Yang Y, Webb GI. On why discretization works for naive-bayes classifiers. In: Australasian Joint Conference on Artificial Intelligence Springer; 2003. p. 440–452.
24. Prugovecki E. Quantum Mechanics in Hilbert Space. Pure and Applied Mathematics, Elsevier Science; 1982.
25. Sakoe H, Chiba S. Dynamic programming algorithm optimization for spoken word recognition. IEEE transactions on acoustics, speech, and signal processing 1978;26(1):43–49.
26. Aach J, Church GM. Aligning gene expression time series with time warping algorithms. Bioinformatics 2001;17(6):495–508.
27. Caiani E, Porta A, Baselli G, Turiel M, Muzzupappa S, Pagani M, et al. Analysis of cardiac left-ventricular volume based on time warping averaging. Medical and Biological Engineering and Computing 2002;40(2):225–233.
28. Giorgino T, et al. Computing and visualizing dynamic time warping alignments in R: the dtw package. Journal of statistical Software 2009;31(7):1–24.
29. Todorovski L, Cestnik B, Kline M, Lavrač N, Džeroski S. Qualitative clustering of short time-series: A case study of firms reputation data. IDDM-2002 2002;p. 141.
30. Slavkov I, Gjorgjioski V, Struyf J, Džeroski S. Finding explained groups of time-course gene expression profiles with predictive clustering trees. Molecular BioSystems 2010;6(4):729–740.
31. Wessel N, Suhrbier A, Riedl M, Marwan N, Malberg H, Bretthauer G, et al. Detection of time-delayed interactions in biosignals using symbolic coupling traces. EPL (Europhysics Letters) 2009;87(1):10004.
32. Marwan N, Romano MC, Thiel M, Kurths J. Recurrence plots for the analysis of complex systems. Physics reports 2007;438(5):237–329.
33. Cover TM, Thomas JA. Elements of information theory. Wiley series in telecommunications, Wiley; 1991.
34. Basso K, Margolin AA, Stolovitzky G, Klein U, Dalla-Favera R, Califano A. Reverse engineering of regulatory networks in human B cells. Nature genetics 2005;37(4):382–390.
35. Margolin AA, Nemenman I, Basso K, Wiggins C, Stolovitzky G, Dalla Favera R, et al. ARACNE: an algorithm for the reconstruction of gene regulatory networks in a mammalian cellular context. BMC bioinformatics 2006;7(1):S7.
36. Faith JJ, Hayete B, Thaden JT, Mogno I, Wierzbowski J, Cottarel G, et al. Large-Scale Mapping and Validation of Escherichia coli Transcriptional Regulation from a Compendium of Expression Profiles. PLOS Biology

2007 01;5(1):1–13. https://doi.org/10.1371/journal.pbio.0050008.

37. Meyer PE, Kontos K, Lafitte F, Bontempi G. Information-theoretic inference of large transcriptional regulatory networks. EURASIP journal on bioinformatics and systems biology 2007;2007(1):79879.
38. Yu D, Parlitz U. Inferring Network Connectivity by Delayed Feedback Control. PLOS ONE 2011 09;6(9):1–12.
39. Van den Bulcke T, Van Leemput K, Naudts B, van Remortel P, Ma H, Verschoren A, et al. SynTReN: a generator of synthetic gene expression data for design and analysis of structure learning algorithms. BMC bioinformatics 2006;7(1):43.
40. de la Fuente A, Stolovitzky G, The DREAM5 Systems Genetics Challenges; 2010.
41. Barrett T, Troup DB, Wilhite SE, Ledoux P, Evangelista C, Kim IF, et al. NCBI GEO: archive for functional genomics data sets—10 years on. Nucleic acids research 2011;39(suppl 1):D1005–D1010.
42. Gasch AP, Spellman PT, Kao CM, Carmel-Harel O, Eisen MB, Storz G, et al. Genomic expression programs in the response of yeast cells to environmental changes. Molecular biology of the cell 2000;11(12):4241–4257.
43. Stehman SV. Selecting and interpreting measures of thematic classification accuracy. Remote sensing of Environment 1997;62(1):77–89.
44. Sokolova M, Lapalme G. A systematic analysis of performance measures for classification tasks. Information Processing & Management 2009;45(4):427–437.
45. Fawcett T. An introduction to ROC analysis. Pattern recognition letters 2006;27(8):861–874.
46. Goadrich M, Oliphant L, Shavlik J. Learning ensembles of first-order clauses for recall-precision curves: A case study in biomedical information extraction. In: International Conference on Inductive Logic Programming Springer; 2004. p. 98–115.
47. Powers DM. Evaluation: from precision, recall and F-measure to ROC, informedness, markedness and correlation 2011;.
48. Davis J, Goadrich M. The relationship between Precision-Recall and ROC curves. In: Proceedings of the 23rd international conference on Machine learning ACM; 2006. p. 233–240.
49. Keilwagen J, Grosse I, Grau J. Area under precision-recall curves for weighted and unweighted data. PLoS One 2014;9(3):e92209.
50. Brodersen KH, Ong CS, Stephan KE, Buhmann JM. The binormal assumption on precision-recall curves. In: Pattern Recognition (ICPR), 2010 20th International Conference on IEEE; 2010. p. 4263–4266.
51. Srinivas N, Deb K. Muiltiobjective optimization using nondominated sorting in genetic algorithms. Evolutionary computation 1994;2(3):221–248.
52. Cox TF, Cox MAA. Multidimensional Scaling, Second Edition. Chapman & Hall: CRC Monographs on Statistics & Applied Probability, CRC Press; 2000.
53. Sing T, Sander O, Beerenwinkel N, Lengauer T. ROCR: visualizing classifier performance in R. Bioinformatics 2005;21(20):3940–3941.
54. Mersmann O. emoa: Evolutionary multiobjective optimization algorithms. R package version 05-0 2012;.

Reference PDF

Click here to access/download
Supplementary Material
kuzmanovski_atal.pdf
