# Supplementary material for: Extensive evaluation of the generalized relevance network approach to inferring gene regulatory networks
Source: Gigascience. 2018 Sep 18;7(11):giy118. doi: 10.1093/gigascience/giy118 (PMC6420648; doi:10.1093/gigascience/giy118)
Supplement: Supplemental File [file giy118_supplemental_file.pdf]

GigaScience, 2017, 1–20

doi: xx.xxxx/xxxx
Manuscript in Preparation
Paper

PAPER

Extensive evaluation of the generalized relevance network approach to inferring gene regulatory networks

Vladimir Kuzmanovski[1,*], Ljupčo Todorovski[1,2] and Sašo Džeroski[1]

[1]Jožef Stefan Institute, Ljubljana,Slovenia and [2]University of Ljubljana, Slovenia

[*]vladimir.kuzmanovski@ijs.si

Abstract

Background: The generalized relevance network approach to network inference reconstructs network links based on the strength of associations between data in individual network nodes. It can reconstruct undirected networks, i.e. relevance networks, *sensu stricto*, as well as directed networks, referred to as causal relevance networks. The generalized approach allows the use of an arbitrary measure of pairwise association between nodes, an arbitrary scoring scheme that transforms the associations into weights of the network links, and a method for inferring the directions of the links. While this makes the approach powerful and flexible, it introduces the challenge of finding a combination of components that would perform well on a given inference task.

Results: We address this challenge by performing an extensive empirical analysis of the performance of 114 variants of the generalized relevance network approach on 47 tasks of gene network inference from time-series data and 39 tasks of gene network inference from steady-state data. We compare the different variants in a multi-objective manner, considering their ranking in terms of different performance metrics. The results suggest a set of recommendations that provide guidance for selecting an appropriate variant of the approach in different data settings.

Conclusions: The association measures based on correlation, combined with a particular scoring scheme of asymmetric weighting, lead to optimal performance of the relevance network approach in the general case. In the two special cases of inference tasks involving short time-series data and/or large networks, association measures based on identifying qualitative trends in the time series are more appropriate.

Key words: network inference; network reconstruction; relevance network approach; gene regulatory networks

Introduction

The genome plays a central role in the control of cellular processes in the organism. The sequencing efforts for different organisms have led to the identification of genes, i.e. individual components of the genome. However, the functions of each gene and its product can not be studied in isolation. To fully understand genome functionality, we have to consider genes and gene products as highly connected and structured networks of information flow through a cell. These biological networks are typically referred to as gene regulatory networks (GRNs), where nodes correspond to genes or gene products and edges correspond to biological or chemical interactions among them.

The paper addresses the task of inference of gene regulatory networks from gene expression data. The rapid advance and wide availability of technology for measuring cellular activities at genome-wide scale have caused enormous interest in methods addressing the GRN inference task in contemporary biology. As a result, a wide repertoire of inference methods has been established [1, 2, 3, 4]. In general, methods for GRN

Compiled on: September 7, 2018.
Draft manuscript prepared by the author.

Key Points

- Recommendations for selecting an appropriate variant of the generalized relevance network approach for a given network inference task
- Comprehensive survey of the variants of the generalized relevance network approach to network inference
- Comparison of the performance of 114 variants of the generalized relevance network approach on 86 tasks of gene networks inference along multiple performance metrics simultaneously

inference take one of two major perspectives on the task [5]. One is the statistical perspective, where the focus is on predicting the presence or absence of interactions between genes and gene products. The other, mathematical modeling perspective, focuses on establishing models that allow for emulating the dynamical activity of the observed natural system.

Methods following the statistical perspective employ a simple *guilt-by-association* heuristic [6, 7], a conjecture that similarity of the expression profiles of a set of genes indicates a shared regulation regime among them. Initially, this conjecture lead to methods for inferring undirected, co-expression and association networks [3], commonly referred to as relevance networks [8]. Later on, these methods were first enriched with statistical techniques for estimating conditional independence to distinguish between direct and indirect interactions [9]. Further on, techniques for inferring the direction of a given network interaction were added to the overall inference methods employed leading to methods for inferring causal, directed networks [10].

Taken together, these developments have led to a generalized relevance network approach [10] that follows the statistical perspective of GRN inference and predicts network links based on the pairwise associations between node expression levels. The degree of a pairwise association is evaluated using a measure of similarity (or distance) between the expression levels of the two corresponding genes. Furthermore, the generalized approach includes scoring methods for refining the original similarity scores towards link weights that distinguish between direct and indirect influences. Finally, the extended approach includes methods for inferring the link directions from data. To make a clear distinction between the original relevance networks and their generalized instances, introduced by Hempel et al. [10], we refer to the later as causal relevance networks (CRNs).

Data for GRN inference come typically from microarray experiments perturbing and stressing genes that produce highly resolved time-series and steady-state measurements of transcript levels. Steady-state measurements are made by perturbing every gene in the network and recording the pseudo state reached after the perturbation. Perturbing every gene is not necessary for time-series data that record gene expression levels over a certain period of time after the perturbation. For both data types, the captured dynamic response of the regulatory effects within a cell should provide robust information about the GRN under consideration [11]. While the original relevance network approach [8] and its extensions towards causal networks [9] have been dealing with steady-state data, the generalized relevance network approach has been proven capable of handling also time-series data [10].

The variety of similarity measures and scoring schemes that can be applied within the generalized relevance network approach, makes the approach flexible and applicable in various scenarios. Many surveys emphasize and focus on the flexibility of the relevance-network approach by presenting and categorizing its variants. The distinctive aspect of the survey presented here is the focus on selecting an appropriate variant of the relevance-network approach for a given data set. Our basic conjecture is that some variants of the approach perform better than others in general, and that the performance of the variant is related to the properties of the data set at hand. Another contribution of our survey is that it includes inference tasks from the two types of data, i.e. steady-state and time-series data.

To test the validity of our conjecture, we perform an extensive comparative analysis of the performance of 114 variants of the relevance network approach on 86 tasks of inferring GRN from data, 47 from time-series data and 39 from steady-state data. The tasks include inference from real microarray measurements of the microorganisms *Escherichia coli* (henceforth *E.coli*) and *Saccharomyces cerevisiae* (*Yeast*) [12, 13, 14], as well as of their simulation counterparts (networks) [10, 15]. Additionally, simulated data from in-silico networks from de la Fuente and Stolovitzky [12] and Marbach et al. [13, 16, 17] have been included in the study.

Our conjecture is analyzed through three dimensions: the impact of the type of data (steady-state vs. time-series), time-series length, and network size on the performance of the different variants of the relevance network approach. The performance of network inference is measured using three different performance metrics widely used for assessment of inferred networks by comparing them to the gold standard, i.e., the set of known "true" interactions among the network nodes.

The study is organized as follows. First, we introduce the generalized relevance network approach and review its variants that stem from different measures of association between network nodes. The survey of the variants of the relevance network approach expose, their theoretical advantages and disadvantages, as well as the history of their applications for inference of gene regulatory networks. Section 3 introduces the experimental setup of the comparative analysis in terms of the GRN tasks addressed, data sets employed, and metrics used to measure the performance of the network inference methods. Section 4 presents and discusses the results, with an emphasis on what they tell as about the most appropriate variant of the relevance network approach for a given GRN inference task. Finally, Section 5 concludes the paper with a brief summary of the comparative analysis and an outline of the directions for further research.

The generalized (causal) relevance network approach

The generalized relevance network approach infers network structure by measuring the pairwise associations between the data observed in the individual network nodes. It follows the more general statistical perspective of GRN inference, where no explicit model of the data is built or assumed. The retrieved knowledge about the pairwise association between nodes is interpreted as the relevance of the individual network links [18, 1].

The relevance network approach has been introduced by Butte and Kohane [8], where pairwise association between gene

expression profiles is measured by using mutual information. Different measures of association based on Euclidean distance and correlation coefficients have been previously used for identifying co-expression of genes [19] or association GRNs [20, 19]. In more recent studies, the generalized network approach approach has been extended to include other association measures and additional steps of interpreting the measured associations, such as the symmetry-breaking methods for identifying directions of links or methods for marginal control of the association [10, 13, 21]. Since they infer link directions and remove spurious links, we can interpret the inferred networks as causal networks, hence the reference causal relevance networks (CRNs).

Hempel et al. [10] decomposes the CRN approach into three components of (1) inference of pairwise associations, (2) marginal control of association, and (3) breaking symmetry. The first step employs a distance measure, a correlation coefficient or a mutual-information measure to assess the association between two network nodes. The result of the first step is a symmetric matrix, the elements of which indicate the strength of the undirected network links. The second step of the marginal control of the association scores includes various scoring schemes, which transform the association-scores matrix into a symmetric or an asymmetric matrix of weights of the network links. The last component, as its name indicates, breaks the symmetry of the symmetric network weights matrices, typically by using a time-shifting technique. Note that the latter can only be used on time-series data.

Overall, the main idea behind the CRN approach is to assign a higher relevance to a hypothesized network link, which is identified with a strong pair-wise association between the corresponding nodes. Thus, the relevance score provides an opportunity for differentiating the possibilities of existence of individual network links. Finally, to obtain the inferred network structure, one has to decide upon a threshold value used to map the numeric relevance score into a discrete binary value to indicate the validity of the initial hypothesis, i.e., the presence of a network link.

The remainder of the section follows the decomposition of the CRN approach into the three components introduced above. First, we introduce all the measures used to estimate pairwise association between nodes in our comparative study of the CRN-approach variants. Next, we introduce the scoring schemes for marginal control of association. Finally, we introduce the time-shifting method for breaking the symmetry of the association/weight matrices.

Association measures

The association measures used in this study can be broadly categorized into three clusters of correlation-based, information-based, and distance-based measures. Correlation-based measures treat the expression profiles as data samples and calculate a correlation coefficient between them. Information-based measures treat the expression profiles as random variables and calculate their non-linear dependence using mutual information. Distance-based measures calculate the association as an inverse of the distance between the observed profiles and can be further clustered in three subgroups. Simple distance measures in the first group treat profiles as vectors. The second group includes a single distance measure of dynamic time wrapping that operates on time series directly. The symbolic measures in the third group operate on symbolic (or qualitative) representations of time-series trends.

In each of the following subsections, we present one of the groups of association measures introduced above. Throughout this section, we use the Greek lower-case letter μ to denote pairwise associations between gene expression profiles and δ for distances between them.

Correlation-based measures

Correlation-based measures consider the expression profiles $x = \langle x_1, x_2, \ldots x_n \rangle$ and $y = \langle y_1, y_2, \ldots y_n \rangle$ as population samples. This allows for the use of an arbitrary correlation coefficient over these samples. In particular, we use three of them in this study: the Pearson, Spearman and Kendall rank correlation coefficients.

The Pearson correlation coefficient quantifies the linear relationship between the samples x and y as

$$\mu_P(x, y) = \frac{\sum_{k=1}^{n}(x_k - \bar{x})(y_k - \bar{y})}{\sqrt{\sum_{k=1}^{n}(x_k - \bar{x})^2} \cdot \sqrt{\sum_{k=1}^{n}(y_k - \bar{y})^2}},$$

where $\bar{x}$ and $\bar{y}$ denote the sample means of x and y, respectively.

As mentioned before, the Pearson correlation coefficient has been first employed for identifying clusters of co-expressed genes by Eisen et al. [19]. Later on, it has been regularly used as a state-of-the-art association measure integrated and compared with other methods [10, 22, 1].

The Spearman rank correlation coefficient is based on the rank distribution of the observed expression values. It can be used as a more general measure of inter-dependencies that is not restricted to linear relationships and defines the inter-dependency between x and y as:

$$\mu_S(x, y) = \mu_P(R(x), R(y))$$

where $R(u) = \langle r(u_1), r(u_2), \ldots r(u_n) \rangle$ and $r(u_k)$ denotes the rank of u in u_k, respectively. The Spearman's rank correlation is often used as an association measure in variants of the CRN approach [23, 24, 1].

The Kendall rank correlation coefficient is a measure of correlation between ranks of two samples x and y defined as [25]:

$$\mu_K(x, y) = \frac{2(n_c - n_d)}{n(n-1)},$$

where n_c is the number of concordant pairs of points in x and y, while n_d is the number of discordant pairs. A concordant pair of time points i and j is concordant, if both $x_i > x_j$ and $y_i > y_j$ or both $x_i < x_j$ and $y_i < y_j$. Otherwise, the pair is discordant. The Kendall's rank correlation is rarely used as an association measure in the variants of the CRN approach, with a few notable exceptions in recent studies [10].

Correlation-based measures applied for inferring associations among genes in a GRN have been widely used in the domain of network inference. The rank correlation coefficient does not necessarily consider a continuous scale of expression vectors; it considers discrete ranks instead. Note, furthermore, that correlation-based measures dismiss the time-component of the time-series data. Finally, note that the resulting pairwise association matrices are symmetric and can not be used to infer the direction of the network links.

Information-based measures

Information-based or information-theoretic measures calculate the association between expression profiles $x = \langle x_1, x_2, \ldots x_n \rangle$ by considering them to be random variables. The most commonly used metric of this group is simple mutual information [26]. In our study, we use it in a combination with different statistical estimators of entropy and discretization methods, introduced below.

Mutual information quantifies the possibly non-linear inter-dependencies between two random variables X and Y. It

can be computed by using different entropy estimators, but usually fails to discover indirect links, representing them as direct links instead, between the nodes in the reconstructed network [27]. The general form of mutual information (MI) relates the marginal entropies of X and Y, $H(X)$ and $H(Y)$, and their joint entropy $H(X,Y)$:

$$\mu_I(x,y) = H(X) + H(Y) - H(X,Y).$$

Three estimators of entropy of a given random variable are being widely used for GRN inference: the maximum likelihood estimator, the Miller-Madow estimator, and the shrink entropy estimator.

The maximum likelihood estimator assesses the entropy of a given empirical distribution of a random variable X following the Shannon entropy definition [28]:

$$H^{emp}(X) = -\sum_{k=1}^{n} p(x_k) \cdot log(p(x_k)).$$

This estimator is highly dependent on the number of bins n and the length of observations, which can increase the bias, while the estimator variance is kept minimal.

The Miller-Madow estimator is based on maximum likelihood estimator, but corrected with a second additive term representing the asymptotic bias:

$$H^{mm}(X) = H^{emp}(X) + \frac{|X| - 1}{2n},$$

where $|X|$ is the number of bins with non-zero probability. The Miller-Madow estimator is preferred over the maximum likelihood estimator, due to the reduction of the bias without increasing the variance or the computational cost [28].

The shrink entropy estimator [29] regularizes the maximum likelihood estimator. The idea is to combine two different estimators, one with low variance and another one with low bias, by using a shrinking factor $\lambda \in [0,1]$:

$$H^{shrink}(X) = -\sum_{k=1}^{n} p_\lambda(x_k) \cdot log(p_\lambda(x_k)),$$

where p_λ is defined as follows:

$$p_\lambda(x_k) = \lambda \frac{1}{|X|} + (1-\lambda)p(x_k).$$

If the value of λ is close to zero, the estimated entropy is close to the value of the basic maximum likelihood estimator; otherwise, if it is close to one, the entropy estimation tends to be closer to the bias term.

The statistical estimators are combined with two different methods for discretization of numeric random variables: equal width and equal frequency. All six possible combinations of estimators and discretization methods are used in the comparative analysis of the CRN-approach variants.

Equal width is a fixed bin-width discretization method, that discretized the values of the numeric variable into equally-sized bins, i.e., ranges of variable values. The **equal frequencies** discretization method partitions the range of the given random variable X into ranges of an equal number of data points. Thus, it results in bins different sizes [30]. In both cases, the default number of bins equals the squared root of the number of observations of the variable [31].

The literature overview reveals that the CRN approach often uses mutual information as measure of association [13, 10, 22, 1, 23, 27]. In that context, Emmert-Streib et al. [1] consider mutual information as a baseline relevance network approach variant. Mutual information is capable of discovering non-linear inter-dependencies, but, as other simple association measures, is not able to infer the direction of influences and produces undirected networks.

Simple distance measures

Simple distance measures assess the strength of gene regulatory interactions by calculating the distance between gene expression profiles. In general, they operate over vectors of values and share same approach or ground norm. Three different measures have been included in the study: the L^{10} Norm (Minkowsky), Euclidean and Manhattan distance.

The ground norm that appears as a basis for all three distance measures is, the so called L^s Norm or Minkowsky distance:

$$\delta_L(x,y) = (\sum_{k=1}^{n} |x_k - y_k|^s)^{(1/s)},$$

where s is represents the dimension of the space in which vectors x and y are compared [32]. We consider three distance measures corresponding to the three norms of L^{10} **Norm** ($s = 10$), **Euclidean distance** ($s = 2$) and **Manhattan distance** ($s = 1$).

The three distance measures share a common limitation of detecting linear inter-dependencies between expression profiles. The determination of gene regulatory interactions is based on raw vectors, the time-component of which is dismissed. They have been applied in the context of relevance networks from the early stage of development of this approach [20] and since then are being regularly used and surveyed [1, 13, 10, 3].

Dynamic time warping

Dynamic time warping (DTW) relies on finding an optimal distance mapping between two time series. It tries to capture differences between time series with regard to time and speed of change. Originally developed in the context of speech recognition [33], it has found its use in a wide range of applications in the domains of medicine and bioinformatics [34, 35].

The algorithm for calculating the DTW measure proceeds in two steps. First, local distances are calculated for all pairs of points from the two time series, using the simple Euclidean distance. Then, the pairs of time points are aligned so that a minimal path is found, where each point is included at least once and the sum of all the distances is minimized. DTW allows specifying constraints of the alignment paths; here we use three of them *symmetric1*, *symmetric2* and *asymmetric*. The descriptions of the constraints can be found in the documentation of the "*dtw*" R-package [36] used in our experiments.

Symbolic measures

The simple qualitative distance is based on a qualitative comparison of the shape or trends of time series. In essence, the simple qualitative distance observes the qualitative trend of change of the time-series values between each pair of time points. It compares the observed qualitative trends in the time-series x against the ones observed in y:

$$\delta_{QD}(x,y) = \sum_{k=1}^{n-1} \sum_{j=k+1}^{n} \frac{2 \cdot Diff(q(x_k,x_j), q(y_k,y_j))}{n \cdot (n-1)},$$

with $Diff(q_1,q_2)$ a function that defines the difference between different qualitative changes, which are defined as *increase* if $x_k < x_j$, *no-change* if $x_k \approx x_j$, and *decrease* if $x_k > x_j$.

The simple qualitative distance has been proposed by Todorovski et al. [37] and has been used in the context of clustering gene expression time series by Slavkov et al. [38]. The simple

qualitative distance can be computed for very short time-series, without decreasing the quality of the estimate of the association between them. Furthermore, it captures the non-linear inter-dependencies between gene expressions.

Symbolic similarity measures operate on symbolic dynamics in order to uncover patterns of interaction. These similarity measures have been applied in the domain of bioinformatics by Wessel et al. [39]. Later, Hempel et al. [10] report upon exhaustive research and application of symbolic similarity measures in the domain of relevance networks.

Symbolic similarity measures transform the observed time series into sequences of symbols [40]. The complete guidance of performing this step is also presented in the work by Hempel et al. [10]. In this study, we include three symbolic similarity measures of **symbol sequence similarity**, **mutual information over symbol vectors**, and **linear combination of both**.

An important disadvantage of these measures is the computation time if the time-series are longer. Hence, possible constraints are applicable with regard to the length of symbol sequences α, which in our case has been determining as follows:

$$\alpha = \begin{cases} [1/n], & \text{if } n < 10 \\ 5, & \text{otherwise} \end{cases}$$

where n denotes the time-series length.

Scoring schemes

Scoring schemes are considered in order to control the resulting association scores, henceforth *weights*. There are various scoring schemes that can go along with above mentioned association measures, but here we limit the set to the ones listed by Hempel et al. [10]: reconstruction of accurate cellular networks, context likelihood of relatedness, maximum relevance/minimum redundancy network, and asymmetric weighting.

Accurate Reconstruction of Accurate Cellular NEtworks (ARACNE) is based on the data processing inequality (DPI) [41] paradigm and states that post-processing cannot improve the already acquired knowledge. In essence, it tests all gene-triplets i,j, and k, where all three pairs have mutual information greater than some threshold I_0. For each such triplet, the edge corresponding to the lowest mutual information I_1 is eliminated from the adjacency matrix:

$$A_{i'j'} = A_{j'i'} = \begin{cases} 0, & \text{if } I_{i'j'} \geq I_2(1-\epsilon) \\ 1, & \text{otherwise} \end{cases} \quad (1)$$

where $I_{i'j'} = argmin\{I_{ij}, I_{jk}, I_{ik}\}$ is the lowest mutual information of the three, I_2 is the second lowest mutual information, and factor ϵ is a tolerance parameter with a value between 0 and 1 [42, 9]. Moreover, ARACNE removes all edges satisfying $I_{i'j'} < \tau$, where τ is predefined threshold [10].

ARACNE is capable of controlling the regulation of a gene over another gene, by modifying the initially inferred network on the basis of mutual information. However, the resulting network is still undirected.

Context Likelihood of Relatedness (CLR) [43] is an extension to the basic relevance network approach proposed by Butte and Kohane [8]. Unlike ARACNE, CLR performs pair-wise comparison of mutual information values. In the second step, it estimates the statistical likelihood of a mutual information value for a given pair of genes (I_{kj}), by comparing it to the marginal (gene-specific) distribution. Thus, two scores are derived, one for gene k and one for gene j. By making the normality assumption about these distributions, the corresponding scores z_k and z_j are calculated as follows:

$$z_k = max(0, \frac{1}{\sigma_k} - \frac{\bar{I}_k}{I_{kj} \cdot \sigma_k}), \quad (2)$$

$$z_j = max(0, \frac{1}{\sigma_j} - \frac{\bar{I}_j}{I_{kj} \cdot \sigma_j}). \quad (3)$$

The final score for a pair of gene is obtained as follows:

$$z_{kj} = \sqrt{z_k^2 + z_j^2}. \quad (4)$$

In contrast to ARACNE, CLR does not rely on a global threshold, but on local background values computed for of each gene separately. The outcome of CLR is an undirected network.

Maximum Relevance/minimum redundancy NETwork (MRNET) is a supervised method that performs a series of maximum relevance/minimum redundancy gene selection procedures [44]. The expression of a given gene is considered as a target $y = x_k$ and the rest of genes from $V = x \setminus x_k$ as descriptive variables in the supervised procedure. Given the set M of selected variables and pairwise weights w_{kj}, the procedure updates M by choosing the variable:

$$x_j^{MRMR} = argmax(s_j), x_j \in V \setminus M, \quad (5)$$

that maximizes the score:

$$s_j = u_j - r_j, \quad (6)$$

where $r_j = \frac{1}{|M|} \sum_{x_i \in M} w_{ji}$ is the redundancy term and $u_j = w_{jk}$ is the relevance term.

The above procedure tries to differentiate between direct and indirect links. Direct links are assigned higher importance (relevance) and indirect links lower importance (higher redundancy). Thus, the entries in the final matrix f_{kj} are calculated as:

$$f_{kj} = \frac{max[(w_{jk} - r_j), (w_{kj} - r_k)]}{w_{kj}}. \quad (7)$$

MRNET assigns weights w based on simple mutual information and employs an additional parameter τ that is used for eliminating edges with an unimportant score. The algorithm is not capable of inferring directionality in the GRN.

Asymmetric WEighting (AWE) is an asymmetric weighting schema based on the topological aspects of a complete set of pairwise weights obtained from a particular association method [10]. Given a matrix, AWE assumes its columns are genes that are regulated by other genes, and its rows are genes that regulate other genes. The asymmetric weights c_{kj} are then calculated by dividing each entry by the sum of the corresponding column scores:

$$c_{kj} = w_{kj} \cdot f_j, \quad (8)$$

$$f_j = (\sum_{k=1}^{m} w_{kj})^{-1}. \quad (9)$$

where f_j corresponds to the amount of regulation received by gene j and c_{kj} to the probability that gene j is regulated by gene k. The probabilities that the jth gene is regulated by each of the other genes sum up to unity:

$$\sum_{k=1}^{m} c_{kj} = \sum_{k=1}^{m} w_{kj} \cdot f_j = 1, \tag{10}$$

This scoring schema is capable of inducing directionality in a GRN. The first application of the schema in the domain of generalized relevance networks is given in Hempel et al. [10].

Time shifting

Time shifting is a method for inferring the direction of an undirected link between two network nodes from time-series data. The main idea is to shift one of the time series, e.g., X in one direction and observe the change of the association $\mu(X, Y)$, using a particular association measure μ. The change of the association measure with the time shift provides information that can be used to infer the direction of the influence, i.e., the direction of the network link. The complete procedure is described by Hempel et al. [10] and Yu and Parlitz [45].

We use the time shifting method as a third, obligatory component of the CRN approach in all cases where the scoring scheme results in undirected network. For the AWE scoring scheme, where the result is a directed network, the use of the time shifting method is optional. In that case, we have two alternative CRN-approach variants: one with and one without applying time shifting. The time shifting method is not applicable in case of steady state data.

Material & methods

In the comparative evaluation of the variants of the CRN approach, we have considered all combinations of association measures and scoring schemes, with time-shifting applied where appropriate. There are 114 candidate combinations corresponding to 114 variants of the relevance network approach. Each of the 114 variants was applied to the 47 tasks of GRN inference from time-series data and 39 tasks of GRN inference from steady-state data. Performance was measured by comparing the inferred network structure with the structure of the given network (in case of reconstructing known networks from simulated data) or with the structure of the best known network (in case of real measurements). We use then performance measures, one of which is the area under the receiver-operator characteristics curve, and the other two are different versions of the area under the precision-recall curve.

The goal of the comparative analysis is to identify the best performing variants of the CRN approach and the properties thereof. We are especially interested in finding out what association measures and scoring schemes work best and what are the interactions between them that lead to the best performance. We also investigate the impact of the time-series length and network size on the best performing variants of the CRN approach.

To identify the best performing methods for a given set of GRN inference tasks, we proceed as follows. First, for each performance measure and each task, we sort and rank the methods in decreasing order with respect to their performance on the task, so the top-performing method gets the rank of 1 and the worst-performing method the rank of 114. Furthermore, for each performance measure, for each method we calculate the average ranks of the method for the given set of tasks. Finally, we perform a Pareto analysis of the three-dimensional space of performance metrics to identify Pareto fronts of points corresponding to the best performing methods, i.e., methods with the lowest average ranks.

The continuation of this section provides further details on the experimental setup for performing the comparative analysis: we first introduce the tasks of GRN inference, then provide a detailed description of the performance metrics used and conclude with a brief overview of the implementation details.

Data description

The comparative study has been conducted using real and simulated micro-array data over time-course and steady-state conditions. In particular, the data or the simulation model used for obtaining data are based on *in silico* networks and real networks of two microorganisms: *Escherichia coli* (*E.coli*) and *Saccharomyces cerevisiae* (*Yeast*);. We use data sets from five previously published studies on GRN inference and related benchmarks.

The first data source is Hempel et al. [10], where datasets were generated by the tool SynTReN [46] on the basis of the well-known gene regulatory networks in *E.coli* and *Yeast*. We consider sub-networks of 100, 150 and 200 genes, characterized by 121, 202 and 303 existing links with an average node degree of 2.42, 2.46 and 3.03, respectively. In order to guarantee consistency between sub-networks and expression data, SynTReN generates different expression data for each selected sub-network. Additionally, three level of noise have been considered: 0.0 (deterministic — without noise), 0.1 and 0.5. These values represent the σ parameter of the log-normal distribution $\sim logX(0, \sigma)$, according to which the noise is generated by SynTReN. For each configuration, 6 technical replicates of 10 time points have been generated and the expression data associated with each gene obtained as the average over the replicates. This is necessary to cope with the non-deterministic nature of the SynTReN data generation algorithm. This source has been employed for time-series analysis only (the 18 data sets labels starting with *E1* and *Y1* in Table 1).

Another source of data are the *DREAM4* [16, 17] and the *DREAM5 challenges* [12, 13]. The former is considered in the analysis of steady-state data, where 10 different *in silico* networks (five of size 10 and five of size 100 genes) have been perturbed with three different approaches, producing, in total, 30 datasets (in Table 2 given with labels starting with *IS2*).

The latter (*DREAM5 challenge*) is considered in the analysis of both time-series and steady-state data. Originally, the challenge provides five networks, out of which we consider three: *Network1*, *Network3* and *Network4*, based on Affymetrix gene expression data of *In silico*, *E.coli* and *Yeast* networks, respectively, taken from the Gene Expression Omnibus (GEO) database [47] and collected under a wide range of biological conditions. For each network, a set of experiments has been performed over its genes.

For the case of time-series analysis, we consider Network3 and Network4 with two experiments per network, and create four tasks (data sets). Network3 contains 4511 genes and 2066 known (existing) links with density of 1.1^{-3}, while Network4 has 5950 genes, 3940 known links and density of 3.8^{-4}. Time series lengths vary from 5 to 48 time points. The four rows in Table 1 with data set labels containing *E2* and *Y2* provide summary description of the four tasks corresponding to the DREAM5 data source for time-series data.

For steady-state data, all three networks are considered, each with one data set. Network1 contains 1643 genes and 3940 interactions (links). Steady-state datasets from Network 1 and Network 3 contains 342 records (observations), while the dataset from Network4 has 238 records. Complete references

are given in Table 2, marked with labels: *IS1_1*, *E2_3* and *Y2_3*, respectively, for Network1, Network3 and Network4.

Organism	Data set	Coverage	Size	Length	Noise
E. coli	*E1_1*	100	100	10	0.0
E. coli	*E1_2*	100	150	10	0.0
E. coli	*E1_3*	100	200	10	0.0
E. coli	*E1_4*	100	100	10	0.1
E. coli	*E1_5*	100	150	10	0.1
E. coli	*E1_6*	100	200	10	0.1
E. coli	*E1_7*	100	100	10	0.5
E. coli	*E1_8*	100	150	10	0.5
E. coli	*E1_9*	100	200	10	0.5
E. coli	*E2_1*	100	4511	6	0
E. coli	*E2_2*	100	4511	5	0
Yeast	*Y1_1*	100	100	10	0.0
Yeast	*Y1_2*	100	150	10	0.0
Yeast	*Y1_3*	100	200	10	0.0
Yeast	*Y1_4*	100	100	10	0.1
Yeast	*Y1_5*	100	150	10	0.1
Yeast	*Y1_6*	100	200	10	0.1
Yeast	*Y1_7*	100	100	10	0.5
Yeast	*Y1_8*	100	150	10	0.5
Yeast	*Y1_9*	100	200	10	0.5
Yeast	*Y2_1*	100	5950	5	0
Yeast	*Y2_2*	100	5950	48	0
Yeast	*Y3_1_2*	100	42	5	0
Yeast	*Y3_1_3*	100	42	5	0
Yeast	*Y3_1_11*	97.6	41	8	0
Yeast	*Y3_1_13*	100	42	5	0
Yeast	*Y3_1_14*	100	42	5	0
Yeast	*Y3_1_15*	100	42	10	0
Yeast	*Y3_2_2*	96	72	5	0
Yeast	*Y3_2_14*	96	72	5	0
Yeast	*Y3_3_2*	96.3	289	5	0
Yeast	*Y3_3_3*	95.7	287	5	0
Yeast	*Y3_3_10*	95.3	286	7	0
Yeast	*Y3_3_13*	96.7	290	5	0
Yeast	*Y3_3_14*	95.7	287	5	0
Yeast	*Y3_4_2*	96.3	181	5	0
Yeast	*Y3_4_3*	95.7	180	5	0
Yeast	*Y3_4_13*	95.2	179	5	0
Yeast	*YI_SON_1*	100	5	10	0
Yeast	*YI_SON_2*	100	5	15	0
Yeast	*YI_SON_3*	100	5	9	0
Yeast	*YI_SON_4*	100	5	9	0
Yeast	*YI_SOFF_1*	100	5	15	0
Yeast	*YI_SOFF_2*	100	5	18	0
Yeast	*YI_SOFF_3*	100	5	18	0
Yeast	*YI_SOFF_4*	100	5	20	0
Yeast	*YI_SOFF_5*	100	5	20	0

Table 1. Properties (columns) of the time-series data sets for 47 GRN inference tasks (rows): organism, data-set label, percentage of network nodes covered in the data set, number of network nodes and time-series length.

The fourth data source provides real measurements, collected as a part of the study conducted by Gasch et al. [14], which aims to explore changes in expression levels of Yeast genes under diverse environmental stresses, such as heat shock, diauxic shift, diamide treatment, and amino acid starvation. The measurements have been taken at different time points, using microarrays. One network has been observed, where four different independent sub-networks were identified, which are considered as separate networks within this study, with size of 42, 75, 300 and 300 nodes. For the observed sub-networks 13 different stresses have been monitored, thus 52 datasets are available. Since some data sets provide limited coverage of the network nodes, we consider only 20 data sets that have network coverage greater than 95%. The time-series observed are of different lengths, from 5 to 11 time points (the last 16 rows in Table 1).

Organism	Data set	Coverage	Size	Records
E. coli	*E2_3*	100	4511	342
Yeast	*Y2_3*	100	5950	238
Yeast	*YI_GLU_1*	100	5	6
Yeast	*YI_GLU_2*	100	5	6
Yeast	*YI_GLU_3*	100	5	6
Yeast	*YI_GAL_1*	100	5	6
Yeast	*YI_GAL_2*	100	5	6
Yeast	*YI_GAL_3*	100	5	6
In silico	*IS1_1*	100	1643	342
In silico	*IS2_1_1*	100	10	10
In silico	*IS2_1_2*	100	10	10
In silico	*IS2_1_3*	100	10	10
In silico	*IS2_2_1*	100	10	10
In silico	*IS2_2_2*	100	10	10
In silico	*IS2_2_3*	100	10	10
In silico	*IS2_3_1*	100	10	10
In silico	*IS2_3_2*	100	10	10
In silico	*IS2_3_3*	100	10	10
In silico	*IS2_4_1*	100	10	10
In silico	*IS2_4_2*	100	10	10
In silico	*IS2_4_3*	100	10	10
In silico	*IS2_5_1*	100	10	10
In silico	*IS2_5_2*	100	10	10
In silico	*IS2_5_3*	100	10	10
In silico	*IS2_6_1*	100	100	100
In silico	*IS2_6_2*	100	100	100
In silico	*IS2_7_1*	100	100	100
In silico	*IS2_7_2*	100	100	100
In silico	*IS2_8_1*	100	100	100
In silico	*IS2_8_2*	100	100	100
In silico	*IS2_9_1*	100	100	100
In silico	*IS2_9_2*	100	100	100
In silico	*IS2_10_1*	100	100	100
In silico	*IS2_10_2*	100	100	100
In silico	*IS2_11_1*	100	100	100
In silico	*IS2_12_1*	100	100	100
In silico	*IS2_13_1*	100	100	100
In silico	*IS2_14_1*	100	100	100
In silico	*IS2_15_1*	100	100	100

Table 2. Properties (columns) of the steady-state data sets for 39 GRN inference tasks (rows): organism, data-set label, percentage of network nodes covered in the data set, number of network nodes and number of records (observations).

The last data source is a benchmark study that proposes a synthetic network for in vivo benchmarking, based on a Yeast gene network [15]. The network is composed of five genes, where the genes regulating each other through a variety of interactions. Microarrays have been measured over time-course and steady-state conditions upon multiple pertrubations. Table 1 shows the properties of the time-series data sets, from this source labeled with YI at the beginning. Similarly, Table 2 present the properties of the steady-state data sets.

Performance metrics

To evaluate the performance of the inference method on a given task, we perform a matching between the structure (links) of the given/known GRN (true network) and the structure (links) of the inferred GRN (inferred network). Since the output of the inference method is a network connectivity matrix containing numeric link weights, we can perform the matching after setting the threshold value that would decide upon the presence

and absence of links. To this end, we set aside metrics that require prior assumptions, i.e., performance metrics that require a predefined or default discrimination threshold. Instead, we follow the standard framework for evaluating network inference and employ *thresholding metrics*, which consider the variability of the discrimination threshold and avoid setting it to a default value. Thus, methods are evaluated with regard to the complete set of possible thresholds, which results in an analysis of the performance space. For this purpose, two different spaces have been applied: *Receiver Operating Characteristic (ROC) curve* and *Precision-Recall (PR) curve* space.

Both spaces are defined over quantities derived from a confusion matrix. A confusion matrix [48, 49] is a matrix that consists of four basic numbers that represent the correctness of link predictions: number of correctly recognized true network links (true positives, *TP*), number of correctly recognized absent links in the true network (true negatives, *TN*), and links that either have been incorrectly predicted to be present (false positives - *FP*) or true network links that were predicted as absent (false negatives, *FN*). These basic numbers are further combined in order to express more specific performance perspectives. In the following formulas, we are going to use *P* to denote the number of true network links and *N* to denote the number of absent links in the true network.

Receiver Operating Characteristic curve (*ROC curve*). This is a two-dimensional space that illustrates the performance of a binary classifier as its discrimination threshold is varied [50]. Its dimensions correspond to the two performance metrics of true positive rate, *TPR* (Eq. 11) and false positive rate, *FPR* (Eq. 12), for various threshold settings. It depicts the relative trade-offs between true positives *TP* and false positives *FP*, which are interpreted as *benefit* and *cost*, respectively.

$$\mathrm{TPR} = \frac{\mathrm{TP}}{\mathrm{P}} = \frac{\mathrm{TP}}{\mathrm{TP} + \mathrm{FN}} \tag{11}$$

$$\mathrm{FPR} = \frac{\mathrm{FP}}{\mathrm{N}} = \frac{\mathrm{FP}}{\mathrm{FP} + \mathrm{TN}} \tag{12}$$

Since the ROC curve is two-dimensional, various summary statistics can be derived from it. Most commonly used is the *Area Under the Curve* (AUC) that quantifies the area that is found below the curve, which is also considered in our study for the comparative evaluation. AUC is calculated by integrating the area under the curve, and express it as a single quantity (area in two-dimensional space).

The ROC space is a unit two-dimensional space with a total area of 1. Thus, it can be plotted on a two-dimensional plot with both axes ranging from 0 to 1. Furthermore, the ROC curve is monotonic, which to a certain extent guarantees that by considering the curve, an optimal threshold can be found. The ROC curve or analysis overall, is suitable for comparison of a classifier with a default classifier (random selection), which is represented in the space as a diagonal line from $(0,0)$ to $(1,1)$.

However, ROC analysis has disadvantages, as well. Mainly, it can be misinterpreted if the problem under consideration is characterized with an imbalanced distribution of class values. This disadvantage can appear due to the fact that true negatives are considered as correct classifications of examples, even though the problem focuses on the correct classification of positive examples (classification of minority class) only. Reconstruction of GRNs is such a problem, where we face networks with many nodes, but a very small number of existing links (minority class), and many non-existing links (majority class). Hence, correct classification of the former is a much more complex task, than the correct classification of the latter. The ROC analysis dismiss the complexity of the classification tasks and considers the correct classification of the minority class of existing links to be equally important as the correct classification of non-existing links.

The AUC quantity, also has its own properties. Its values range from 0 to 1: values close to 1 represent better classifiers, while values around 0.5 mean that the classifier is no better than the default (random) classifier. Values below 0.5 mean that the evaluated classifier behaves worse than the default classifier. The disadvantages of the ROC curve are reflected also in the AUC quantity. Namely, considering the problem of GRN reconstruction, we can end up with overall high AUC, increased mainly by the accurate classification of the majority class (correctly predicting non-existing links).

Precision-Recall curve (*PR curve*) is also a two-dimensional space that defines the performance of a binary classifier as its discrimination threshold is varying [51, 52]. Commonly, it is used as a replacement for the ROC curve in the case of highly imbalanced class distribution [53]. The space is defined with two metrics derived from the confusion matrix: *recall* (Eq 13) and *precision* (Eq. 14).

$$\mathrm{recall} = \mathrm{TPR} = \frac{\mathrm{TP}}{\mathrm{P}} = \frac{\mathrm{TP}}{\mathrm{TP} + \mathrm{FN}} \tag{13}$$

$$\mathrm{precision} = \frac{\mathrm{TP}}{\mathrm{TP} + \mathrm{FP}} \tag{14}$$

Summary statistics can also be derived from the PR curve, such as the commonly used *Area Under the PR Curve* (AUPRC). In this analysis, we used two different portions of the area under the curve: partial *AUPRC-0.2* and total area *AUPRC*. Guided by the importance of discovering only true links, without an expectation that all of them would be discovered, we consider the 20% of the AUPRC that corresponds to lower recall (up to 0.2), referred to as *AUPRC-0.2*. It means that we try to evaluate a classifier in accordance to the top scored predictions for true links. So, if the classifier has high precision within this region (sub-space), then it is considered to be good classifier and can assure that those links that are predicted with very high scores are true links.

Unlike the ROC curve, the PR curve is not monotonic and therefore, the performance can vary by varying the discrimination threshold. The curve is plotted in two-dimensional unit space, with a total area of 1, starts from the point $(0,1)$ and finishes at the point $(1,0)$. There is no PR curve for a default (random) classifier, but exist some agreements on how it could be plotted on a graph [53]. The advantage of PR curves over ROC curves is the fact that, for the former, the class imbalance does not affect correct performance estimation. This is because *TN* are excluded from the calculations. The exclusion of *TN*, however, breaks the monotonic property of the curve. The PR curve can be very sparse in terms of points when dealing with imbalanced data. Therefore, interpolation is recommended [53, 54]: To this end, we employ the implementation by Brodersen et al. [55].

Similarly to *AUC*, *AUPRC* values are in the range from 0 to 1, while *AUPRC-0.2* values range from 0 to 0.2, where higher values indicate better performance. In the continuation of the paper, we will refer to the three performance measures introduced here as AUROC, AUPRC and rAUPRC (restricted AUPRC).

Ranking of methods

The methods we evaluate are the 114 variants of the CRN approach. An exhaustive list of the variants is given in Appendix

Table 3. The joint ranking of the CRN-approach variants along the mean rankings with respect to the three performance measures of AUROC, AUPRC and rAUPRC. The method rankings are averaged over all the tasks from Table 1. Each row corresponds to a single Pareto front of non-dominated points: the column PF-I reports the Pareto front index, Dom-HV the volume of the space dominated by the Pareto front, and the last column includes the CRN-approach variants corresponding to the Pareto-front points. The labels of the CRN-approach variants are explained in Appendix A. For example, the 3rd Pareto-front consists of two variants *csp-ns ckd-ms*. The first letter of both variants (**c**) corresponds to *correlation* group of association measures. The second and the third letters describe the association measure itself: *Spearman* and *Kendall* correlation coefficients, respectively for both variants. Finally, the last two letters refer to combination of scoring scheme and time-shifting approach, where the first **n** of the first variant corresponds to *None* (constant or ID scoring scheme applied), the first letter of the second variant (**m**) shows that MRNET scoring scheme has been applied. In both cases, the last letter that refers to time-shifting use is **s**, which corresponds to *Spearman* time-shifting approach.

PF-I	Dom-HV	CRN-approach variants
1	0.3789	cpr-ns cpr-ws csp-ws ckd-ws sqd-cs
2	0.3504	cpr-wn csp-wn ckd-wn mfm-ns
3	0.2778	csp-ns ckd-ms
4	0.2586	ckd-ns mfm-ws
5	0.2368	mfm-wn saf-wn
6	0.2141	csp-ms mfo-ms mwo-ws mfm-ms saf-ns saw-ns saw-wn saw-ws
7	0.1976	cpr-cs mwo-wn mwo-ms mfm-as sqd-ms saf-ws
8	0.1730	mwo-cs mfs-wn mfs-ms smf-wn saw-cs
9	0.1657	csp-cs mfo-wn mfm-cs mfs-ws smf-ws smw-wn
10	0.1563	ckd-cs mfo-ns mfo-as mfo-ws smw-ns smw-ws
11	0.1339	cpr-ms mfo-cs mwo-ns smf-cs
12	0.1166	mfs-ns smf-ns saf-cs
13	0.1073	ws1-as mfs-as smw-cs
14	0.0943	dmn-as dec-as mfs-cs sss-cs
15	0.0822	d10-as sqd-wn sqd-ws smw-ms saw-ms
16	0.0767	sqd-ns smf-ms saf-ms
17	0.0637	ckd-as mwo-as sqd-as
18	0.0501	csp-as sss-ms
19	0.0293	sss-ns sss-as sss-wn sss-ws

A, Table 1-5. The tables (and first letter of the labels used to denote the variants) corresponds to the groups of association measures aforementioned.

To rank the variants of the CRN approach according to their performance on the 47 tasks for GRN inference from time-series data and 39 tasks for GRN inference from steady-state data, we first filter out the low-performing methods by testing the statistical significance of the difference between the measured method performance and the performance of a random classifier. In particular, we employ the one-sample Student's t-test to check whether the average performance of a given method on the GRN inference tasks is significantly higher than 0.5 and 0.1, respectively, for the expected AUROC and AUPRC performance of a random classifier, and 0.2 (for the rAUPRC performance measure). Methods that are not significantly better than a random classifier with respect to at least one of the three performance measures are excluded from further analysis.

In the next step, for each performance measure and each GRN inference task, we rank the CRN-approach variants according to their performance on the particular task. Then, we average each method's ranks over all the tasks to obtain three mean rankings of the CRN-approach variants with respect to the AUROC, AUPRC and rAUPRC measures. To obtain a joint ranking along the three performance measures, we employ the non-dominated sorting algorithm used in multi-objective decision theory [56]. We first embed the variants into a three-dimensional space, where each dimension corresponds to a ranking of the CRN-approach variants with respect to one of the performance measures. Each CRN-approach variant corresponds to a single point in that space, where each coordinate value is the rank of the method according to a particular performance measure. Note that we normalize the method rankings on the [0, 1] scale, using a simple linear transformation $(r_M - 1)/(N - 1)$, where r_M is the rank of the method M, while N denotes the number of all compared methods. Figure 1 depicts the projection of the three-dimensional space in two dimensions, obtained by using multidimensional scaling [57]. The red, green, and blue labels and gray points in the graph correspond to the compared CRN-approach variants.

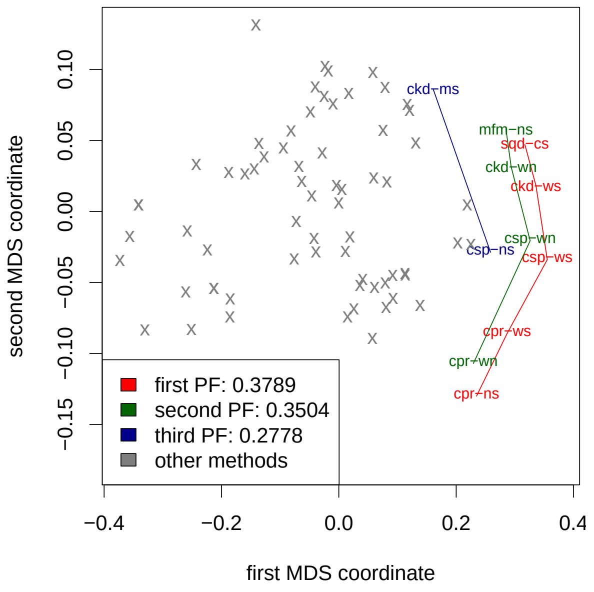

Figure 1. The first three Pareto fronts (PF) in the three-dimensional space of mean rankings of the variants of the CRN approach with respect to the three performance measures of AUROC, AUPRC and rAUPRC. The rankings of the variants are averaged over all the network reconstruction tasks from Table 1. Each number in the legend represents the hypervolume dominated by the points in the corresponding Pareto front. The two-dimensional projection of the three-dimensional space was obtained using multidimensional scaling.

To identify the top-ranked CRN-approach variants, we search for a set of non-dominated points in the three-dimensional space, i.e., we identify the Pareto front of the non-dominated points in the space. The points in the Pareto front correspond to the methods that are the best performers according to at least one performance measure. After we assign the top ranks to these (Figure 1) CRN-approach variants, we remove the corresponding points from the three-dimensional space and iteratively continue to identify Pareto fronts in the reduced sets of points until all the methods are ranked.

Table 3 presents the joint ranking along the three performance measures obtained with the non-dominated sorting algorithm described above. For each Pareto front, we can calculate the hypervolume of the space dominated by the points on the front. The volume change indicates the magnitude of differences between rankings of the methods in two Pareto fronts. Figure 1 depicts the first three Pareto fronts (red, green and blue points) in the two-dimensional projection of the original three-dimensional space. They include the 13 top-performing methods according to the three performance measures taken together simultaneously.

Results

In the comparative analysis of the performance of the CRN-approach variants, the focus on the top-ranked variants included in the first three Pareto fronts identified with the non-dominated sorting algorithm. For each CRN-approach variant in these Pareto fronts, we analyze its composition in terms of the association measure and the scoring scheme employed. We proceed with the analysis as follows. First, we identify the overall top-performing methods on the (a) 47 tasks of GRN inference from time-series data listed in Table 1 and (b) 39 tasks of GRN inference from steady-state data listed in Table 2. Next, for experiments with time-series data, we analyze the impact of time-series length and network size on the performance of CRN-approach variants. For the experiments with steady-state data, we analyze the impact of network size only, since the number of records in the data set (its size) is strongly correlated to the network size (compare the values in the last two columns of Table 2).

Time-series data

The comparison of performance on all the tasks of GRN inference from time-series data identifies the correlation-based measures (Figure 2, left) as the top performing ones. Correlation-based association measures appear most frequently among the best performers in all three Pareto fronts, dominating by total number of CRN-approach variants. In total, 9 (out of the 11) variants use correlation coefficient in the top three Pareto fronts. In the first Pareto front, correlation-based measures are represented in four (out of five) variants, followed by three appearances (out of four) in the second Pareto front, and two in the third. Variants based on the symbolic and mutual-information measures represent the second most-frequent group in the CRN-approach variants that dominate the performance space. They appear in the first two Pareto fronts, once in each.

In contrast with the clear differences in performance among the association measures, the scoring schemes can not be so clearly differentiated (Figure 2, right). Namely, all scoring schemes (except ARACNE) appear within the top three dominant Pareto fronts. The AWE scoring scheme is the most frequent one: it appears in six CRN-approach variants in the first two Pareto fronts. In the first Pareto front, AWE is a component of three variants: in all three variants it is combined with the time-shifting method (label WS). In the second Pareto front, the AWE scoring scheme is used without the time-shifting method (label WN). The time-shifting method used without scoring scheme (label NS) appears three times among the top performing CRN-approach variants (once per each Pareto front). Each of the remaining two scoring schemes (MS and CS) appears once among the top-performing CRN-approach variants in the three Pareto fronts.

The impact of time-series length

To investigate the impact of time-series length (l) on the performance of the CRN-approach variants, we clustered the 47 data sets into two groups of tasks with short ($l < 10$, 20 tasks) and long ($l \geq 10$, 27 tasks) time series.

Figure 3A provides overview of the seven top-performing CRN-approach variants on the tasks involving short time series. The most frequent association measure among the top performers is the symbolic measure (label S, Figure 3A, left). Four CRN-approach variants that include symbolic association measures are found in the top two Pareto fronts. Association measures based on mutual information (label M) appear in two variants in the second and third Pareto front. Scoring scheme analysis (Figure 3A, right) does not show dominance of any particular scoring scheme: except MRNET, all are found among the top-performing variants. The first Pareto front includes two different scoring schemes (labels WN and WS representing AWE as well as CS representing CLR, the latter two combined with time shifting) indicating that the symbolic association measures can be equally well combined with the two scoring schemes of AWE and CLR when tackling tasks involving short time-series data.

The comparison of CRN-approach variants on tasks involving long time series, presented in Figure 3B, leads to different results. Among the 12 top-ranked CRN-approach variants, the correlation-based association measures prevail: they participate in eight variants distributed among all three Pareto fronts (Figure 3B, left). Association measures based on mutual information appear in three variants, two of them being in the first Pareto front. A single variant that uses a symbolic association measure is found in the third Pareto front. The results indicate that for long time series, one should prefer correlation over alternative association measures. Differences with respect to the results on short time series are visible for scoring schemes as well (Figure 3C, right). The AWE scoring scheme appears in nine variants among the 12 top performers: four times in combination with time shifting, and five times without.

In sum, we can conclude that the selection of an appropriate association measure for a given task depends on the time-series length. For short time series, symbolic association measures should be used, while for long ones, one should opt for the correlation-based association measures.

The impact of network size

We consider the number of network nodes or genes (n) to be a measure of the network size. When analyzing its impact on the performance of the CRN-approach variants, we cluster the GRN inference tasks into two groups of tasks of inferring small ($n \leq 100$, 23 tasks) and large ($n > 100$, 20 tasks) networks.

Figure 4 depicts the top-performing variants of the CRN approach for tasks involving networks with different sizes. The single top-performing association measure for tasks involving small networks is correlation (see Figure 4A, left). The distribution of scoring schemes among the seven top-performing variants emphasizes AWE with and without time shifting (labels WS and WN, respectively) present in five out of seven top-ranked variants. Two variants without scoring scheme appear in the second and third Pareto front (Figure 4A, right). In sum,

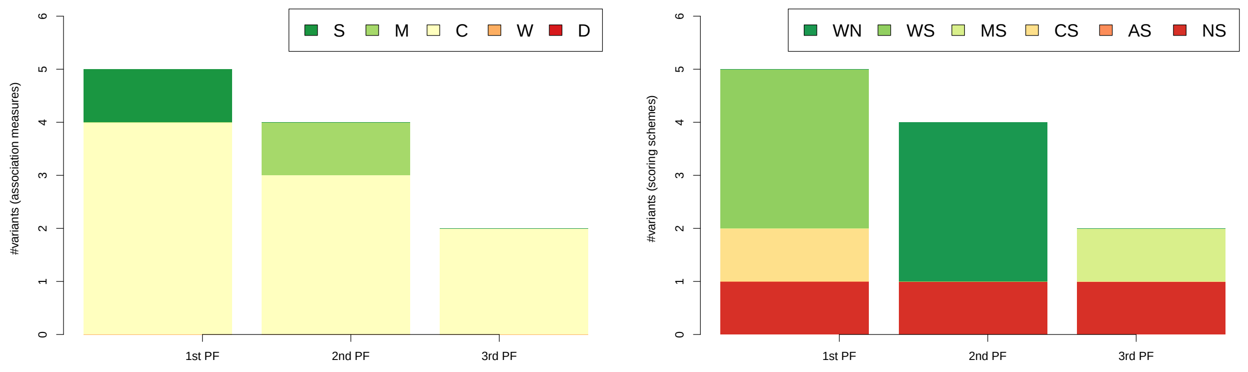

Figure 2. The association measures (left-hand side) and scoring schemes (right-hand side) used by the 11 top-ranked variants of the CRN approach from the first three Pareto fronts (PF) in the three-dimensional space of AUROC-AURPC-rAUPRC mean rankings. The rankings are averaged over the 47 tasks of GRN inference from time-series data listed in Table 1. Legend on the left-hand side: S denotes the class of symbolic and qualitative association measures, M — association measures based on mutual information, C — correlation-based, W — dynamic time warping, and D — distance-based measures. Legend on the right-hand side: WN denotes the AWE scoring scheme without time shifting, WS, MS, CS and AS — the AWE, MRNET, CLR and ARACNE with time shifting, and NS — the time-shifting method without a scoring scheme.

for GRN inference tasks involving small networks, one should opt for a combination of a correlation-based association measure and the AWE scoring scheme.

A slightly different distribution of association measures is observed among the top-performing CRN-approach variants on tasks involving large networks (Figure 4B). Among the 15 top-performing variants approach, six employ symbolic association measures, four employ measures based on correlation and a single one in the third Pareto front employs mutual information (Figure 4B, left). Similarly to the results on the small networks, AWE is prevailing as a scoring scheme used by the top-performing variants (appearing in 11 out of 15), followed by variants without a scoring scheme (Figure 4B, right).

In sum, the selection of an appropriate association measure for a given task depends on the network size. For tasks involving small networks (up to 100 nodes), correlation-based association measures should be used, while for large ones, one should also consider symbolic measures as another valid option.

Steady-state data

The comparison of performance on all the tasks of GRN inference from steady-state data identifies the CRN-approach variants involving association measures based on correlation and mutual information (Figure 5, left) as the top performing ones. Association measures based on mutual information are involved in five out of the 12 top-performing variants (all five being in the first two Pareto fronts). Correlation-based association measures appear among the best performers in all three Pareto fronts, dominating by total number of CRN-approach variants. In total, seven variants using correlation coefficients are found in the leading three Pareto fronts.

In contrast with the notable differences in performance among the variants with different association measures, all scoring schemes can be found among the 12 top-performing variants (Figure 2, right). The AWE scoring scheme (label WN) can be found in four out of 11 top-performing variants, while MRNET can be found in three.

Note that the results obtained on steady-state data tasks resemble the one obtained on tasks involving time-series data. Again, the top-performing CRN-approach variants are the ones using association measures based on correlation and mutual information. Both can be considered valid options for composing a CRN variant to tackle an inference task involving time-series or steady-state data.

In contrast, the experimental results of evaluating the impact of network size on the performance of CRN-approach variants on tasks involving steady-state data show no impact of the network size on the performance (Figure 6). For both small (Figure 6A, left) and large (Figure 6B, left) networks, the association measures of choice are based on correlation and mutual information. The results on the selection of the scoring scheme are once again, inconclusive, since all scoring schemes are used by the top-performing variants in both groups of tasks involving small and large networks (Figure 6, right). In any case, we can conclude from Figure 6 that the network size does not influence the selection of an appropriate CRN-approach variant.

Discussion

The goal of the discussion presented here is to address the main issue raised in the introduction, i.e., the issue of "What Works Where?" or *What would be a reasonable choice of association measure and scoring scheme in the generalized relevance network approach for a given task of GRN inference*? The overview of the results of the experiments on all 86 tasks provides a relatively simple answer: the CRN-approach variants using correlation-based associations measure and the AWE scoring scheme perform best. Furthermore, the results show that the AWE scoring scheme works equally well with or without time-shifting for inferring link directions from time-series data.

Note that both correlation-based measures and the AWE scoring schemes work over data samples and vectors. This fact already indicates that the particular combination would work equally well for time-series (where the temporal component is largely ignored) and steady-state data. Furthermore, correlation-based measures perform well with other scoring schemes, except for ARACNE. The observation that correlation-based methods, when combined with certain scoring scheme, yield overall performance improvements on time-series tasks, leads to the conclusion that they can perform well with time-shifting only, but in fact, performance improvements can also be gained by selecting an appropriate scoring scheme.

Symbolic association measures have been identified as the second best performing group of measures that are frequently present among the top-performing CRN-approach variants. In contrast with the correlation-based measures, they operate on temporal data only, and are therefore useful only in the context of time-series data. Also, symbolic measures appear to per-

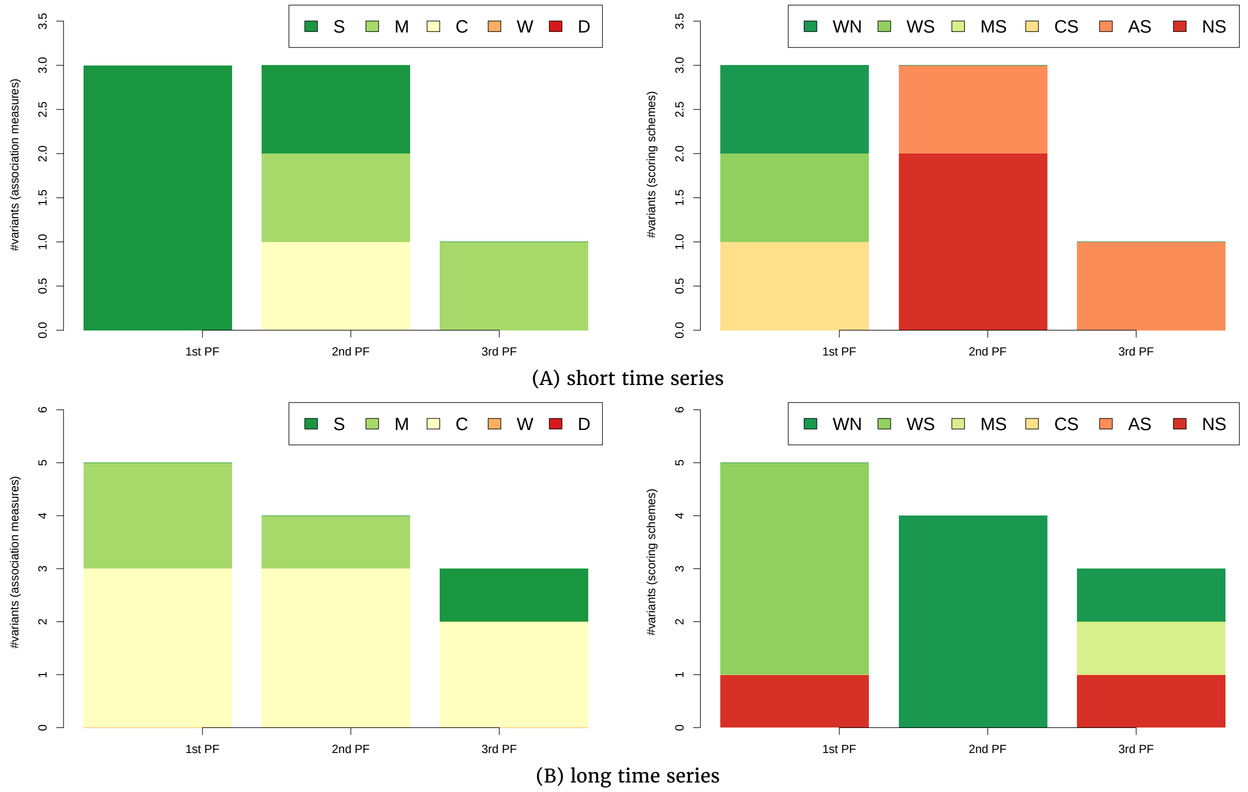

Figure 3. The association measures (left-hand side) and scoring schemes (right-hand side) used in the seven (A) and 12 (B) top-ranked variants of the CRN approach from the first three Pareto fronts in the three-dimensional space of AUROC-AURPC-rAUPRC mean rankings. The rankings are averaged on the GRN inference tasks involving short (A, 20 tasks) and long (B, 27 tasks) time series.

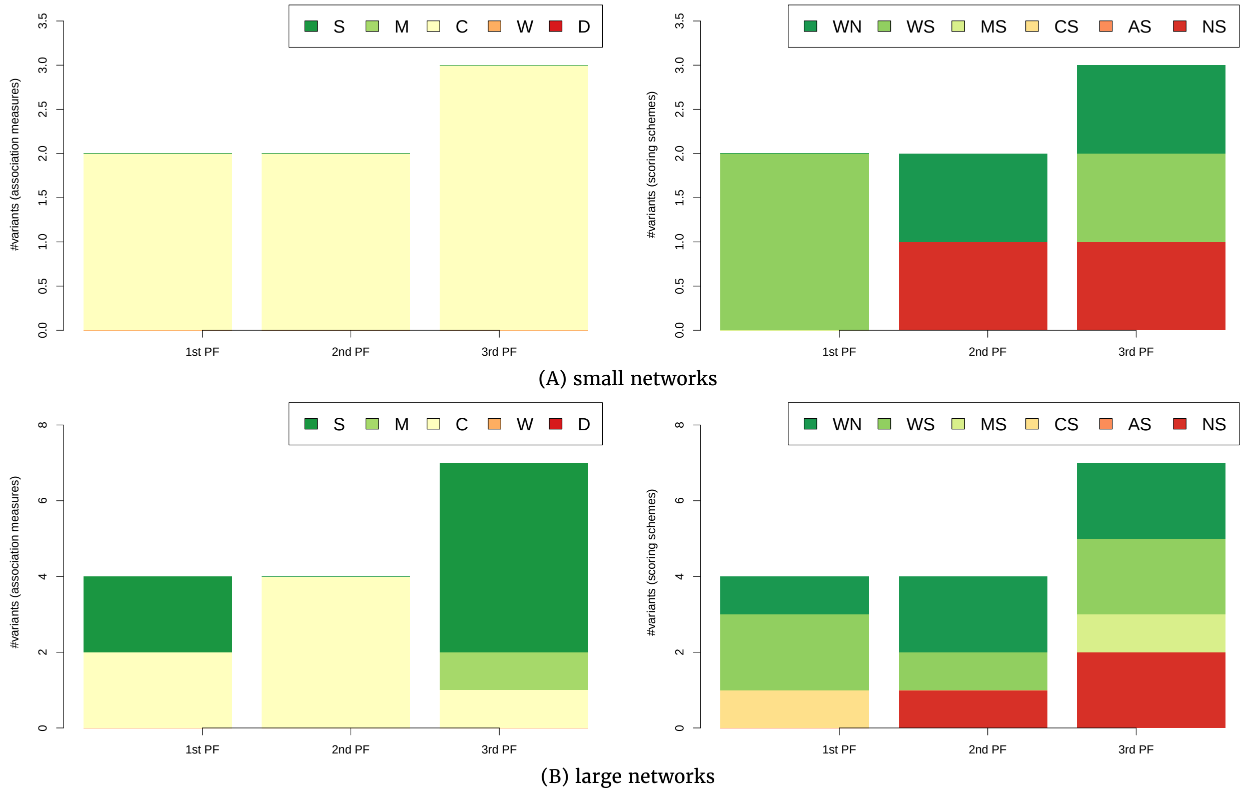

Figure 4. The association measures (left-hand side) and scoring schemes (right-hand side) used in the seven (A) and 15 (B) top-ranked variants of the CRN approach from the first three Pareto fronts in the three-dimensional space of AUROC-AURPC-rAUPRC mean rankings. The rankings are averaged over the GRN inference tasks involving small (A, 20 tasks of inference from time-series data) and large (B, 27 tasks of inference from time-series data) networks.

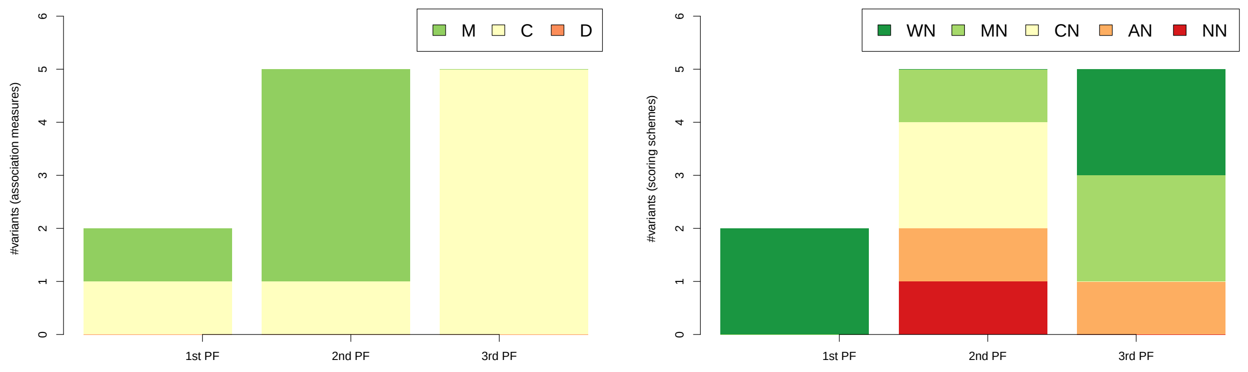

Figure 5. The association measures (left-hand side) and scoring schemes (right-hand side) used by the 12 top-ranked variants of the CRN approach from the first three Pareto fronts (PF) in the three-dimensional space of AUROC-AURPC-rAUPRC mean rankings. The rankings are averaged over the 39 tasks of GRN inference from steady-state data listed in Table 2. Legend on the left-hand side: M denotes the class of association measures based on mutual information, C — correlation-based and D — distance-based association measures. Legend on the right-hand side: WN, MN, CN and AN denote CRN-approach variants using the AWE, MRNET, CLR and ARACNE scoring scheme, while NN — variants without scoring scheme.

form well only in combination with the AWE scoring scheme.

Overall, correlation-based association measures show robustness with regard to the selection of a scoring scheme, while the AWE scoring scheme improves performance in general, without limiting the choice of an association measure. For tasks involving steady-state data, top-performing CRN-approach variants also include association measures based on mutual information.

The results of the analysis of method performance on data sets with varying time-series length reveal further "*What Works Where?*" insights. For short time series, symbolic and mutual information association measures lead to top-performing variants of the CRN approach. Symbolic measures behave robust and work well in combination with all scoring schemes, except the one that applies time-shifting only. This leads to the conclusion that symbolic association measures are robust in general and give more flexibility in choosing a scoring scheme, but need to be corrected by a scoring scheme prior to inferring the links directions. Unlike the symbolic association measures, the ones based on mutual information do not show robustness with respect to the selection of a scoring schemes. They perform well only if combined with the ARACNE scoring scheme or the time-shifting method applied without any scoring scheme.

The dominance of the correlation-based association measures increases in the setting of long time series. Namely, they have been identified in most of the tasks as part of the best performing method compositions. However, they seem to perform well only in combination with scoring scheme AWE, while time-shifting only and MRNET are observed among top performers in one case only. Competing with correlation-based CRN-approach variants are those based on mutual information with strong limitation in choosing a scoring scheme, i.e., AWE with or without time-shifting.

In sum, when addressing an inference task involving short time series, symbolic association measures are recommended as a robust solution. For long time series, these measures become more dependent on a limited set of scoring schemes. This is a result of the fact that they examine the associations exhaustively throughout the time point's space. Therefore, for shorter time series they are capable of complete search of the space, which is not a case for longer time series, where they are constrained due to computational complexity. Conclusively, correlation-based measures can be recommended as a robust solution for long time series, since they are not constrained by computational complexity issue for retrieve knowledge from larger amount of data.

The comparative analysis of method performance over different network sizes shows more consistent results over different settings on the tasks involving steady-state data. Namely, correlation-based association measures outperform all other measures across all network sizes.

For tasks involving inference of small networks from time-series data, the correlation-based group of measures performs well in combination with all scoring schemes, except ARACNE and MRNET. As in the general case, ARACNE performs aggressive cutoffs of inferred links, without considering the difference between estimated associations. However, from the observations we can conclude that correlation-based association measures are the most robust solution and allow flexibility in choosing a scoring scheme and construction of a customized CRN approach. For tasks involving inference of large networks from time-series data, the association measures of choice are the symbolic ones and mutual information. The former are to be combined with the AWE scoring scheme, while the later can be used without a scoring scheme or combined with AWE.

Finally, worth mentioning is the observation that distance-based association measures have not been identified among the best performing association measures in either of the settings considered. Thus, they are excluded from the list of recommended groups of association measures worth considering for tasks of GRN inference from time-series or steady-state data.

Conclusion

The comparative analysis presented in this paper is based on an extensive empirical evaluation of the performance of 114 variants of the general relevance network approach on 86 tasks of inferring gene regulatory networks from time-series and steady-state data. The 114 CRN-approach variants are based on 6 general classes of association measures (with a variety of parameter settings) and 6 scoring schemes, some of which are accompanied by a time-shifting method for inference of the direction of network links from time-series data. The performance of the CRN-approach variants is measured using three different performance metrics widely used in other studies on inferring gene regulatory networks from data.

The main contribution of this paper is the general framework for comparative evaluation of the numerous variants of the general relevance network approach to inference of gene regulation networks. The proposed framework is flexible and modular: one can easily extend it along any dimension of comparison, such as adding new association measures, scoring schemes, performance metrics or network inference tasks. The

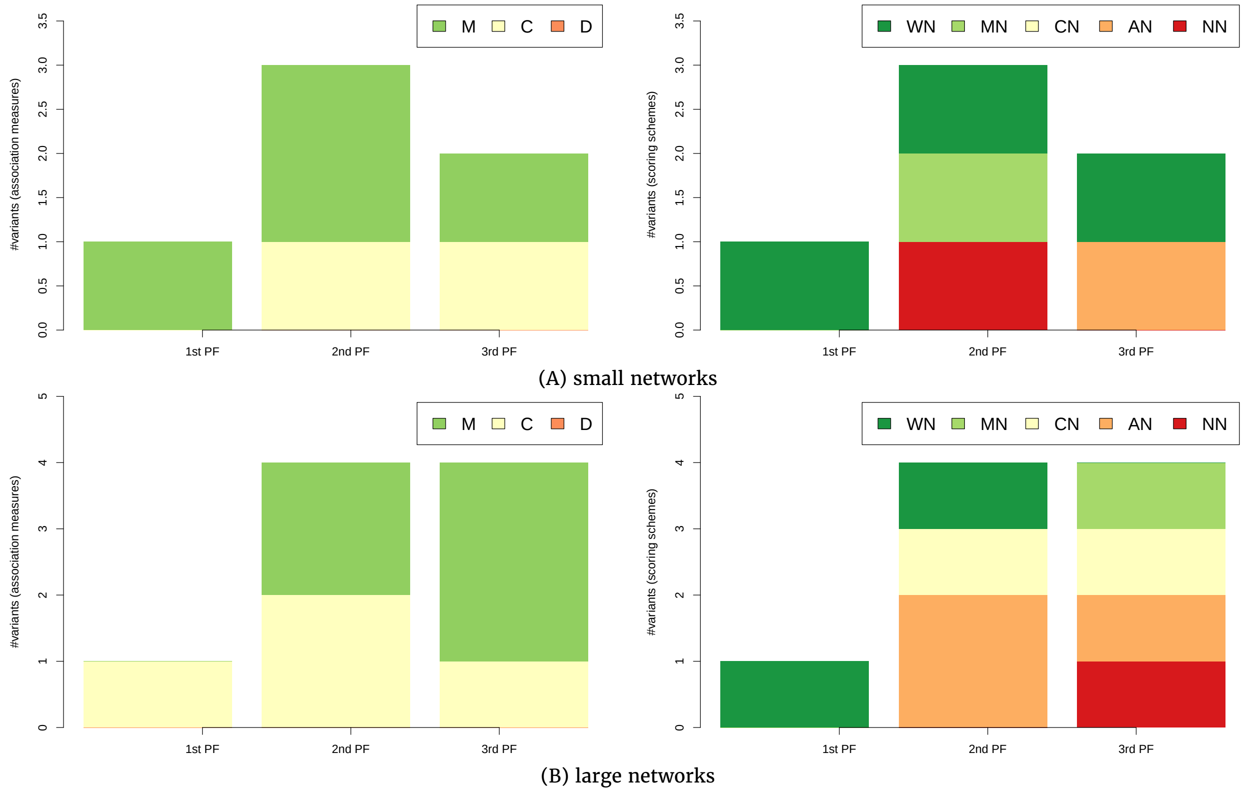

Figure 6. The association measures (left-hand side) and scoring schemes (right-hand side) used in the six (A) and nine (B) top-ranked variants of the CRN approach from the first three Pareto fronts in the three-dimensional space of AUROC-AURPC-rAUPRC mean rankings. The rankings are averaged over the GRN inference tasks involving small (A, 21 tasks of inference from steady-state data) and large (B, 18 tasks of inference from steady-state data) networks.

publicly available source code of the implemented framework allows for simple implementation of such extensions, as well as reproducing the results presented in this study.

The main motivation for the evaluation performed in this paper is answering the question "*What works where?*". The answer provides important guidance for applying the generalized relevance network approach in a particular situation in terms of selecting an appropriate combination of an association measure and a scoring scheme that would lead to reasonably good performance on a given data set. Another important aspect of our survey and comparative analysis is that it involves tasks of inference from both time-series and steady-state data. The results of the comparative analysis lead to the following list of recommendations for configuring the generalized relevance network approach:

- In general, the safest combination is a correlation-based association measure with the Asymmetric WEighting (AWE) scoring scheme for both time-series and steady-state data.
- The association measures based on simple distances and dynamic time warping never lead to a top-performing variant of the CRN approach.
- For short time series (with less than ten time points), the general class of symbolic association measures (and the qualitative distance measure in particular) leads to best performing variants of the CRN approach. These measures can be combined with an arbitrary scoring scheme.
- For long time series (of at least 10 time points), the general recommendation is to combine a correlation-based association measure with the AWE scoring scheme applies.
- For large networks with more than 100 nodes, symbolic association measures (combined with the AWE scoring scheme) gain an edge over the correlation-based ones when inducing GRNs from time-series data.

While this set of recommendations provides clear guidance for selecting an appropriate variant of the generalized relevance network approach, further experiments are necessary to strengthen the generality of the results. This is especially true for the results on the impact of network size: too few large-size networks are included in the current set of inference tasks. In further work, one would extend the set of inference tasks with ones that involve networks with a varying number of nodes.

Future work could also exploit important source of input for the relevance network approach, not considered in this study, namely expert knowledge about the presence or absence of certain links in the network. Note, however, that none of these limitations of out study should represent an obstacle for applying the proposed framework for empirical evaluation. The framework is flexible enough to be used for comparative analysis on extended sets of GRN tasks and CRN-approach variants (methods).

Availability of Data and Materials

We implemented all the components and the variants of the CRN approach in the R software environment for statistical computing. We implemented most of the components using standard R functions, except for the association measure based on the dynamic time warping (DTW) measure of distance between time series, for which we used the DTW implementation in the R package *dtw* [36]. The source code of our implementation of the CRN-approach variants in R are publicly available:

- Project name: RN-approach project
- Project home page: https://vkuzmanovski@bitbucket.org/

vkuzmanovski/rn-approach.git
- Operating system(s): Platform independent
- Programming language: R
- Other requirements: None
- License: FreeBSD
- RRID: SCR_016488 (SciCrunch.org)

To calculate the values of the performance metrics, we have used the functions implemented in the R package for evaluating the performance of classifiers *ROCR* [58]. For performing the Pareto analysis, we used the implementation of the non-dominated sorting algorithm in the R package for multi-objective optimization *emoa* [59]. The source code of the R functions used to perform the comparative analysis of the CRN-approach variants that allows for complete reconstruction of its results is also publicly available:

- Project name: RN-evaluation project
- Project home page: https://vkuzmanovski@bitbucket.org/vkuzmanovski/rn-evaluation.git
- Operating system(s): Platform independent
- Programming language: R
- Other requirements: None
- License: FreeBSD

The complete materials, including data and source code, are available publicly through a *GigaDB repository* [60], as well.

Authors' contributions

S.D. initiated the study, formulated the general methodological problem and the specific application domain. V.K. and L.T. designed and implemented the computational and evaluation framework. All authors conceived and planned the experiments, while V.K. and L.T. performed them and analyzed their results. V.K. and L.T. drafted the manuscript. All authors reviewed and approved the final manuscript.

Funding

The study has been financially supported by the Slovenian Research Agency (through research core fundings: No. P2-0103, No. P5-0093, as well as the project No. N2-0056, Machine Learning for Systems Sciences), the Slovenian Ministry of Education, Science and Sport (through the funding agreement No. C3330-17-529020) and the European Commission (through the grants MAESTRA, HBP SGA2, and LANDMARK).

Acknowledgment

The authors are immensely grateful for the support obtained from dr. rer. nat. Sabrina Hempel (Leibniz-Institute for Agricultural Engineering and Bioeconomy (ATB), Germany) and Prof. dr. Zoran Nikoloski (University of Potsdam, Germany). They provided implementation of the Asymmetric WEighting relevance network method for gene network inference. They also provided a part of the datasets used in this study and helped to better understand them.

References

1. Emmert-Streib F, Glazko G, De Matos Simoes R, et al. Statistical inference and reverse engineering of gene regulatory networks from observational expression data. Frontiers in genetics 2012;3:8.
2. De Jong H. Modeling and simulation of genetic regulatory systems: a literature review. Journal of computational biology 2002;9(1):67–103.
3. Markowetz F, Spang R. Inferring cellular networks–a review. BMC bioinformatics 2007;8(6):S5.
4. Hecker M, Lambeck S, Toepfer S, Van Someren E, Guthke R. Gene regulatory network inference: data integration in dynamic models — a review. Biosystems 2009;96(1):86–103.
5. Frank Emmert-Streib MD, Haibe-Kains B. Untangling statistical and biological models to understand network inference: the need for a genomics network ontology. Frontiers in Genetics;5:299.
6. Joshua M Stuart DKSKK Eran Segal. A Gene-Coexpression Network for Global Discovery of Conserved Genetic Modules. Science 2003;302:249–255.
7. Stolovitzky G, Monroe D, Califano A. Dialogue on Reverse-Engineering Assessment and Methods. Annals of the New York Academy of Sciences 2007;1115(1):1–22.
8. Butte AJ, Kohane IS. Mutual information relevance networks: functional genomic clustering using pairwise entropy measurements. In: Pac Symp Biocomput, vol. 5; 2000. p. 26.
9. Margolin AA, Nemenman I, Basso K, Wiggins C, Stolovitzky G, Dalla Favera R, et al. ARACNE: an algorithm for the reconstruction of gene regulatory networks in a mammalian cellular context. BMC bioinformatics 2006;7(1):S7.
10. Hempel S, Koseska A, Nikoloski Z, Kurths J. Unraveling gene regulatory networks from time-resolved gene expression data–a measures comparison study. BMC bioinformatics 2011;12(1):292.
11. Penfold CA, Wild DL. How to infer gene networks from expression profiles, revisited. Interface focus 2011;1(6):857–870.
12. de la Fuente A, Stolovitzky G, The DREAM5 Systems Genetics Challenges; 2010.
13. Marbach D, Costello JC, Küffner R, Vega NM, Prill RJ, Camacho DM, et al. Wisdom of crowds for robust gene network inference. Nature methods 2012;9(8):796–804.
14. Gasch AP, Spellman PT, Kao CM, Carmel-Harel O, Eisen MB, Storz G, et al. Genomic expression programs in the response of yeast cells to environmental changes. Molecular biology of the cell 2000;11(12):4241–4257.
15. Cantone I, Marucci L, Iorio F, Ricci MA, Belcastro V, Bansal M, et al. A Yeast Synthetic Network for In Vivo Assessment of Reverse-Engineering and Modeling Approaches. Cell 2009;137(1):172 – 181. http://www.sciencedirect.com/science/article/pii/S0092867409001561.
16. Marbach D, Schaffter T, Mattiussi C, Floreano D. Generating realistic in silico gene networks for performance assessment of reverse engineering methods. Journal of computational biology 2009;16(2):229–239.
17. Marbach D, Prill RJ, Schaffter T, Mattiussi C, Floreano D, Stolovitzky G. Revealing strengths and weaknesses of methods for gene network inference. Proceedings of the national academy of sciences 2010;107(14):6286–6291.
18. Werhli AV, Grzegorczyk M, Husmeier D. Comparative evaluation of reverse engineering gene regulatory networks with relevance networks, graphical gaussian models and bayesian networks. Bioinformatics 2006;22(20):2523–2531.
19. Eisen MB, Spellman PT, Brown PO, Botstein D. Cluster analysis and display of genome-wide expression patterns. Proceedings of the National Academy of Sciences 1998;95(25):14863–14868.
20. Rays M, Chen Y, Su YA. Use of a cDNA microarray to analyse gene expression patterns in human cancer. Nature ge-

netics 1996;14.
21. Ceci M, Pio G, Kuzmanovski V, Džeroski S. Semi-supervised multi-view learning for gene network reconstruction. PloS one 2015;10(12):e0144031.
22. Hempel S, Koseska A, Nikoloski Z. Data-driven reconstruction of directed networks. The European Physical Journal B 2013;86(6):250.
23. Zhang B, Horvath S, et al. A general framework for weighted gene co-expression network analysis. Statistical applications in genetics and molecular biology 2005;4(1):1128.
24. Horvath S, Dong J. Geometric interpretation of gene coexpression network analysis. PLoS comput biol 2008;4(8):e1000117.
25. Kendall MG. A new measure of Rank correlation. Biometrika 1938;30(1-2):81.
26. de Matos Simoes R, Emmert-Streib F. Influence of Statistical Estimators of Mutual Information and Data Heterogeneity on the Inference of Gene Regulatory Networks. PLOS ONE 2011 12;6(12):1–14.
27. Soranzo N, Bianconi G, Altafini C. Comparing association network algorithms for reverse engineering of large-scale gene regulatory networks: synthetic versus real data. Bioinformatics 2007;23(13):1640.
28. Paninski L. Estimation of entropy and mutual information. Neural computation 2003;15(6):1191–1253.
29. Schäfer J, Strimmer K, et al. A shrinkage approach to large-scale covariance matrix estimation and implications for functional genomics. Statistical applications in genetics and molecular biology 2005;4(1):32.
30. Yang Y, Webb GI. On why discretization works for naive-bayes classifiers. In: Australasian Joint Conference on Artificial Intelligence Springer; 2003. p. 440–452.
31. Meyer PE, Lafitte F, Bontempi G. minet: AR/Bioconductor package for inferring large transcriptional networks using mutual information. BMC bioinformatics 2008;9(1):461.
32. Prugovecki E. Quantum Mechanics in Hilbert Space. Pure and Applied Mathematics, Elsevier Science; 1982.
33. Sakoe H, Chiba S. Dynamic programming algorithm optimization for spoken word recognition. IEEE transactions on acoustics, speech, and signal processing 1978;26(1):43–49.
34. Aach J, Church GM. Aligning gene expression time series with time warping algorithms. Bioinformatics 2001;17(6):495–508.
35. Caiani E, Porta A, Baselli G, Turiel M, Muzzupappa S, Pagani M, et al. Analysis of cardiac left-ventricular volume based on time warping averaging. Medical and Biological Engineering and Computing 2002;40(2):225–233.
36. Giorgino T, et al. Computing and visualizing dynamic time warping alignments in R: the dtw package. Journal of statistical Software 2009;31(7):1–24.
37. Todorovski L, Cestnik B, Kline M, Lavrač N, Džeroski S. Qualitative clustering of short time-series: A case study of firms reputation data. IDDM-2002 2002;p. 141.
38. Slavkov I, Gjorgjioski V, Struyf J, Džeroski S. Finding explained groups of time-course gene expression profiles with predictive clustering trees. Molecular BioSystems 2010;6(4):729–740.
39. Wessel N, Suhrbier A, Riedl M, Marwan N, Malberg H, Bretthauer G, et al. Detection of time-delayed interactions in biosignals using symbolic coupling traces. EPL (Europhysics Letters) 2009;87(1):10004.
40. Marwan N, Romano MC, Thiel M, Kurths J. Recurrence plots for the analysis of complex systems. Physics reports 2007;438(5):237–329.
41. Cover TM, Thomas JA. Elements of information theory. Wiley series in telecommunications, Wiley; 1991.
42. Basso K, Margolin AA, Stolovitzky G, Klein U, Dalla-Favera R, Califano A. Reverse engineering of regulatory networks in human B cells. Nature genetics 2005;37(4):382–390.
43. Faith JJ, Hayete B, Thaden JT, Mogno I, Wierzbowski J, Cottarel G, et al. Large-Scale Mapping and Validation of Escherichia coli Transcriptional Regulation from a Compendium of Expression Profiles. PLOS Biology 2007 01;5(1):1–13. https://doi.org/10.1371/journal.pbio.0050008.
44. Meyer PE, Kontos K, Lafitte F, Bontempi G. Information-theoretic inference of large transcriptional regulatory networks. EURASIP journal on bioinformatics and systems biology 2007;2007(1):79879.
45. Yu D, Parlitz U. Inferring Network Connectivity by Delayed Feedback Control. PLOS ONE 2011 09;6(9):1–12.
46. Van den Bulcke T, Van Leemput K, Naudts B, van Remortel P, Ma H, Verschoren A, et al. SynTReN: a generator of synthetic gene expression data for design and analysis of structure learning algorithms. BMC bioinformatics 2006;7(1):43.
47. Barrett T, Troup DB, Wilhite SE, Ledoux P, Evangelista C, Kim IF, et al. NCBI GEO: archive for functional genomics data sets—10 years on. Nucleic acids research 2011;39(suppl 1):D1005–D1010.
48. Stehman SV. Selecting and interpreting measures of thematic classification accuracy. Remote sensing of Environment 1997;62(1):77–89.
49. Sokolova M, Lapalme G. A systematic analysis of performance measures for classification tasks. Information Processing & Management 2009;45(4):427–437.
50. Fawcett T. An introduction to ROC analysis. Pattern recognition letters 2006;27(8):861–874.
51. Goadrich M, Oliphant L, Shavlik J. Learning ensembles of first-order clauses for recall-precision curves: A case study in biomedical information extraction. In: International Conference on Inductive Logic Programming Springer; 2004. p. 98–115.
52. Powers DM. Evaluation: from precision, recall and F-measure to ROC, informedness, markedness and correlation 2011;.
53. Davis J, Goadrich M. The relationship between Precision-Recall and ROC curves. In: Proceedings of the 23rd international conference on Machine learning ACM; 2006. p. 233–240.
54. Keilwagen J, Grosse I, Grau J. Area under precision-recall curves for weighted and unweighted data. PLoS One 2014;9(3):e92209.
55. Brodersen KH, Ong CS, Stephan KE, Buhmann JM. The binormal assumption on precision-recall curves. In: Pattern Recognition (ICPR), 2010 20th International Conference on IEEE; 2010. p. 4263–4266.
56. Srinivas N, Deb K. Muiltiobjective optimization using non-dominated sorting in genetic algorithms. Evolutionary computation 1994;2(3):221–248.
57. Cox TF, Cox MAA. Multidimensional Scaling, Second Edition. Chapman & Hall: CRC Monographs on Statistics & Applied Probability, CRC Press; 2000.
58. Sing T, Sander O, Beerenwinkel N, Lengauer T. ROCR: visualizing classifier performance in R. Bioinformatics 2005;21(20):3940–3941.
59. Mersmann O. emoa: Evolutionary multiobjective optimization algorithms. R package version 05-0 2012;.
60. Kuzmanovski V, Todorovski L, Džeroski S, Supporting data for "Extensive evaluation of the generalized relevance network approach to inferring gene regulatory networks". GigaScience Database; 2018. http://dx.doi.org/10.5524/100492.

APPENDIX A: List of CRN variants

All variants of CRN-approach considered in the study are given in the following tables with full description of their abbreviations. As mentioned before, each variant consists of combination of association measure, scoring scheme and time-shifting, each of which is given as a column in the tables below. All variants are categorized in five categories, based on type of association measure: distance-based measures, dynamic time warping variants, correlation-based measures, mutual information-based measures, and symbolic measures. The last column in each table shows the data type(s) over which particular method has been applied: *ts* refers to "time-series" and *ss* refers to "steady-state" data type.

Table 1. List of CRN variants within *distance-based* association measures.

Abbreviation	Association measure	Scoring scheme	Time-shifting	Data type
**dmn-nn**	Manhattan	None	None	ss
**dmn-ns**	Manhattan	None	Spearman	ts
**dmn-an**	Manhattan	ARACNE	None	ss
**dmn-as**	Manhattan	ARACNE	Spearman	ts
**dmn-wn**	Manhattan	AWE	None	ts,ss
**dmn-ws**	Manhattan	AWE	Spearman	ts
**dmn-cn**	Manhattan	CLR	None	ss
**dmn-cs**	Manhattan	CLR	Spearman	ts
**dmn-mn**	Manhattan	MRNET	None	ss
**dmn-ms**	Manhattan	MRNET	Spearman	ts
**dec-nn**	Euclidean	None	None	ss
**dec-ns**	Euclidean	None	Spearman	ts
**dec-an**	Euclidean	ARACNE	None	ss
**dec-as**	Euclidean	ARACNE	Spearman	ts
**dec-wn**	Euclidean	AWE	None	ts,ss
**dec-ws**	Euclidean	AWE	Spearman	ts
**dec-cn**	Euclidean	CLR	None	ss
**dec-cs**	Euclidean	CLR	Spearman	ts
**dec-mn**	Euclidean	MRNET	None	ss
**dec-ms**	Euclidean	MRNET	Spearman	ts
**d10-nn**	L-10 norm (Minkowsky)	None	None	ss
**d10-ns**	L-10 norm (Minkowsky)	None	Spearman	ts
**d10-an**	L-10 norm (Minkowsky)	ARACNE	None	ss
**d10-as**	L-10 norm (Minkowsky)	ARACNE	Spearman	ts
**d10-wn**	L-10 norm (Minkowsky)	AWE	None	ts,ss
**d10-ws**	L-10 norm (Minkowsky)	AWE	Spearman	ts
**d10-cn**	L-10 norm (Minkowsky)	CLR	None	ss
**d10-cs**	L-10 norm (Minkowsky)	CLR	Spearman	ts
**d10-mn**	L-10 norm (Minkowsky)	MRNET	None	ss
**d10-ms**	L-10 norm (Minkowsky)	MRNET	Spearman	ts

Table 2. List of CRN variants within *dynamic time warping (DTW)* association measure with variants.

Abbreviation	Association measure	Scoring scheme	Time-shifting	Data type
**was-ns**	DTW - Asymmetric constraint path	None	Spearman	ts
**was-as**	DTW - Asymmetric constraint path	ARACNE	Spearman	ts
**was-wn**	DTW - Asymmetric constraint path	AWE	None	ts
**was-ws**	DTW - Asymmetric constraint path	AWE	Spearman	ts
**was-cs**	DTW - Asymmetric constraint path	CLR	Spearman	ts
**was-ms**	DTW - Asymmetric constraint path	MRNET	Spearman	ts
**ws1-ns**	DTW - Symmetric constraint path (1)	None	Spearman	ts
**ws1-as**	DTW - Symmetric constraint path (1)	ARACNE	Spearman	ts
**ws1-wn**	DTW - Symmetric constraint path (1)	AWE	None	ts
**ws1-ws**	DTW - Symmetric constraint path (1)	AWE	Spearman	ts
**ws1-cs**	DTW - Symmetric constraint path (1)	CLR	Spearman	ts
**ws1-ms**	DTW - Symmetric constraint path (1)	MRNET	Spearman	ts
**ws2-ns**	DTW - Symmetric constraint path (2)	None	Spearman	ts
**ws2-as**	DTW - Symmetric constraint path (2)	ARACNE	Spearman	ts
**ws2-wn**	DTW - Symmetric constraint path (2)	AWE	None	ts
**ws2-ws**	DTW - Symmetric constraint path (2)	AWE	Spearman	ts
**ws2-cs**	DTW - Symmetric constraint path (2)	CLR	Spearman	ts
**ws2-ms**	DTW - Symmetric constraint path (2)	MRNET	Spearman	ts

Table 3. List of CRN variants within *correlation-based* association measures.

Abbreviation	Association measure	Scoring scheme	Time-shifting	Data type
**cpr-nn**	Pearson	None	None	ss
**cpr-ns**	Pearson	None	Spearman	ts
**cpr-an**	Pearson	ARACNE	None	ss
**cpr-as**	Pearson	ARACNE	Spearman	ts
**cpr-wn**	Pearson	AWE	None	ts,ss
**cpr-ws**	Pearson	AWE	Spearman	ts
**cpr-cn**	Pearson	CLR	None	ss
**cpr-cs**	Pearson	CLR	Spearman	ts
**cpr-mn**	Pearson	MRNET	None	ss
**cpr-ms**	Pearson	MRNET	Spearman	ts
**csp-nn**	Spearman	None	None	ss
**csp-ns**	Spearman	None	Spearman	ts
**csp-an**	Spearman	ARACNE	None	ss
**csp-as**	Spearman	ARACNE	Spearman	ts
**csp-wn**	Spearman	AWE	None	ts,ss
**csp-ws**	Spearman	AWE	Spearman	ts
**csp-cn**	Spearman	CLR	None	ss
**csp-cs**	Spearman	CLR	Spearman	ts
**csp-mn**	Spearman	MRNET	None	ss
**csp-ms**	Spearman	MRNET	Spearman	ts
**ckd-nn**	Kendall	None	None	ss
**ckd-ns**	Kendall	None	Spearman	ts
**ckd-an**	Kendall	ARACNE	None	ss
**ckd-as**	Kendall	ARACNE	Spearman	ts
**ckd-wn**	Kendall	AWE	None	ts,ss
**ckd-ws**	Kendall	AWE	Spearman	ts
**ckd-cn**	Kendall	CLR	None	ss
**ckd-cs**	Kendall	CLR	Spearman	ts
**ckd-mn**	Kendall	MRNET	None	ss
**ckd-ms**	Kendall	MRNET	Spearman	ts

Table 4. List of CRN variants within *mutual information-based* association measures.

Abbreviation	Association measure	Scoring scheme	Time-shifting	Data type
**mfo-nn**	Mutual information – Equal frequency (Miller-Madow estimator)	None	None	ss
**mfo-ns**	Mutual information – Equal frequency (Miller-Madow estimator)	None	Spearman	ts
**mfo-an**	Mutual information – Equal frequency (Miller-Madow estimator)	ARACNE	None	ss
**mfo-as**	Mutual information – Equal frequency (Miller-Madow estimator)	ARACNE	Spearman	ts
**mfo-wn**	Mutual information – Equal frequency (Miller-Madow estimator)	AWE	None	ts,ss
**mfo-ws**	Mutual information – Equal frequency (Miller-Madow estimator)	AWE	Spearman	ts
**mfo-cn**	Mutual information – Equal frequency (Miller-Madow estimator)	CLR	None	ss
**mfo-cs**	Mutual information – Equal frequency (Miller-Madow estimator)	CLR	Spearman	ts
**mfo-mn**	Mutual information – Equal frequency (Miller-Madow estimator)	MRNET	None	ss
**mfo-ms**	Mutual information – Equal frequency (Miller-Madow estimator)	MRNET	Spearman	ts
**mwo-nn**	Mutual information – Equal width (Miller-Madow estimator)	None	None	ss
**mwo-ns**	Mutual information – Equal width (Miller-Madow estimator)	None	Spearman	ts
**mwo-an**	Mutual information – Equal width (Miller-Madow estimator)	ARACNE	None	ss
**mwo-as**	Mutual information – Equal width (Miller-Madow estimator)	ARACNE	Spearman	ts
**mwo-wn**	Mutual information – Equal width (Miller-Madow estimator)	AWE	None	ts,ss
**mwo-ws**	Mutual information – Equal width (Miller-Madow estimator)	AWE	Spearman	ts
**mwo-cn**	Mutual information – Equal width (Miller-Madow estimator)	CLR	None	ss
**mwo-cs**	Mutual information – Equal width (Miller-Madow estimator)	CLR	Spearman	ts
**mwo-mn**	Mutual information – Equal width (Miller-Madow estimator)	MRNET	None	ss
**mwo-ms**	Mutual information – Equal width (Miller-Madow estimator)	MRNET	Spearman	ts
**mfm-nn**	Mutual information – Equal frequency (Maximum likelihood estimator)	None	None	ss
**mfm-ns**	Mutual information – Equal frequency (Maximum likelihood estimator)	None	Spearman	ts
**mfm-an**	Mutual information – Equal frequency (Maximum likelihood estimator)	ARACNE	None	ss
**mfm-as**	Mutual information – Equal frequency (Maximum likelihood estimator)	ARACNE	Spearman	ts
**mfm-wn**	Mutual information – Equal frequency (Maximum likelihood estimator)	AWE	None	ts,ss
**mfm-ws**	Mutual information – Equal frequency (Maximum likelihood estimator)	AWE	Spearman	ts
**mfm-cn**	Mutual information – Equal frequency (Maximum likelihood estimator)	CLR	None	ss
**mfm-cs**	Mutual information – Equal frequency (Maximum likelihood estimator)	CLR	Spearman	ts
**mfm-mn**	Mutual information – Equal frequency (Maximum likelihood estimator)	MRNET	None	ss
**mfm-ms**	Mutual information – Equal frequency (Maximum likelihood estimator)	MRNET	Spearman	ts
**mfs-nn**	Mutual information – Equal frequency (Shrink entropy estimator)	None	None	ss
**mfs-ns**	Mutual information – Equal frequency (Shrink entropy estimator)	None	Spearman	ts
**mfs-an**	Mutual information – Equal frequency (Shrink entropy estimator)	ARACNE	None	ss
**mfs-as**	Mutual information – Equal frequency (Shrink entropy estimator)	ARACNE	Spearman	ts
**mfs-wn**	Mutual information – Equal frequency (Shrink entropy estimator)	AWE	None	ts,ss
**mfs-ws**	Mutual information – Equal frequency (Shrink entropy estimator)	AWE	Spearman	ts
**mfs-cn**	Mutual information – Equal frequency (Shrink entropy estimator)	CLR	None	ss
**mfs-cs**	Mutual information – Equal frequency (Shrink entropy estimator)	CLR	Spearman	ts
**mfs-mn**	Mutual information – Equal frequency (Shrink entropy estimator)	MRNET	None	ss
**mfs-ms**	Mutual information – Equal frequency (Shrink entropy estimator)	MRNET	Spearman	ts

Table 5. List of CRN variants within *symbolic* association measures.

Abbreviation	Association measure	Scoring scheme	Time-shifting	Data type
**sqd-ns**	Simple **q**ualitative **d**istance	None	Spearman	ts
**sqd-as**	Simple **q**ualitative **d**istance	ARACNE	Spearman	ts
**sqd-wn**	Simple **q**ualitative **d**istance	AWE	None	ts
**sqd-ws**	Simple **q**ualitative **d**istance	AWE	Spearman	ts
**sqd-cs**	Simple **q**ualitative **d**istance	CLR	Spearman	ts
**sqd-ms**	Simple **q**ualitative **d**istance	MRNET	Spearman	ts
**sss-ns**	Symbol sequence similarity	None	Spearman	ts
**sss-as**	Symbol sequence similarity	ARACNE	Spearman	ts
**sss-wn**	Symbol sequence similarity	AWE	None	ts
**sss-ws**	Symbol sequence similarity	AWE	Spearman	ts
**sss-cs**	Symbol sequence similarity	CLR	Spearman	ts
**sss-ms**	Symbol sequence similarity	MRNET	Spearman	ts
**smw-ns**	Mutual information (equal **w**idth) over symbol vectors	None	Spearman	ts
**smw-as**	Mutual information (equal **w**idth) over symbol vectors	ARACNE	Spearman	ts
**smw-wn**	Mutual information (equal **w**idth) over symbol vectors	AWE	None	ts
**smw-ws**	Mutual information (equal **w**idth) over symbol vectors	AWE	Spearman	ts
**smw-cs**	Mutual information (equal **w**idth) over symbol vectors	CLR	Spearman	ts
**smw-ms**	Mutual information (equal **w**idth) over symbol vectors	MRNET	Spearman	ts
**smf-ns**	Mutual information (equal **f**requency) over symbol vectors	None	Spearman	ts
**smf-as**	Mutual information (equal **f**requency) over symbol vectors	ARACNE	Spearman	ts
**smf-wn**	Mutual information (equal **f**requency) over symbol vectors	AWE	None	ts
**smf-ws**	Mutual information (equal **f**requency) over symbol vectors	AWE	Spearman	ts
**smf-cs**	Mutual information (equal **f**requency) over symbol vectors	CLR	Spearman	ts
**smf-ms**	Mutual information (equal **f**requency) over symbol vectors	MRNET	Spearman	ts
**saw-ns**	Average of *sss-ns* and *smw-ns*	None	Spearman	ts
**saw-as**	Average of *sss-as* and *smw-as*	ARACNE	Spearman	ts
**saw-wn**	Average of *sss-wn* and *smw-wn*	AWE	None	ts
**saw-ws**	Average of *sss-ws* and *smw-ws*	AWE	Spearman	ts
**saw-cs**	Average of *sss-cs* and *smw-cs*	CLR	Spearman	ts
**saw-ms**	Average of *sss-ms* and *smw-ms*	MRNET	Spearman	ts
**saf-ns**	Average of *sss-ns* and *smf-ns*	None	Spearman	ts
**saf-as**	Average of *sss-as* and *smf-as*	ARACNE	Spearman	ts
**saf-wn**	Average of *sss-wn* and *smf-wn*	AWE	None	ts
**saf-ws**	Average of *sss-ws* and *smf-ws*	AWE	Spearman	ts
**saf-cs**	Average of *sss-cs* and *smf-cs*	CLR	Spearman	ts
**saf-ms**	Average of *sss-ms* and *smf-ms*	MRNET	Spearman	ts
