# Supplementary material for: Extensive evaluation of the generalized relevance network approach to inferring gene regulatory networks
Source: Gigascience. 2018 Sep 18;7(11):giy118. doi: 10.1093/gigascience/giy118 (PMC6420648; doi:10.1093/gigascience/giy118)
Supplement: GIGA-D-17-00222_Revision_1.pdf [file giy118_giga-d-17-00222_revision_1.pdf]

GigaScience

Extensive evaluation of the generalized relevance network approach to inferring gene regulatory networks

--Manuscript Draft--

**Manuscript Number:**	GIGA-D-17-00222R1
**Full Title:**	Extensive evaluation of the generalized relevance network approach to inferring gene regulatory networks
**Article Type:**	Research
**Funding Information:**	Javna Agencija za Raziskovalno Dejavnost RS (P2-0103) — Dr Vladimir Kuzmanovski, Prof. Dr. Sašo Džeroski<br>Javna Agencija za Raziskovalno Dejavnost RS (P5-0093) — Dr Ljupčo Todorovski<br>Javna Agencija za Raziskovalno Dejavnost RS (N2-0056) — Dr Ljupčo Todorovski, Prof. Dr. Sašo Džeroski<br>European Commission (ICT-2013-612944 MAESTRA) — Dr Ljupčo Todorovski, Prof. Dr. Sašo Džeroski
**Abstract:**	The generalized relevance network approach to network inference reconstructs network links based on the strength of associations between data in individual network nodes. It can reconstruct undirected networks, i.e. relevance networks, sensu stricto approach, as well as directed networks, approach referred to as causal relevance networks. The generalized approach allows the use of an arbitrary measure of pairwise association between nodes, an arbitrary scoring scheme that transforms the associations into weights of the network links, and a method for inferring the directions of the network links. While this makes the generalized relevance network approach powerful and flexible, it introduces the challenge of finding a combination of components that would perform well on a given inference task. We address this challenge by performing an extensive empirical analysis of the performance of 114 variants of the generalized relevance network approach on 47 tasks of gene network inference from time-series data and 39 tasks of gene network inference from steady-state data. The results of the evaluation reveal that the association measures based on correlation combined with a particular scoring scheme of asymmetric weighting lead to optimal performance of the relevance network approach in the general case. In the two special cases of inference tasks involving short time-series data and/or large networks, association measures based on identifying qualitative trends in the time series are more appropriate.
**Corresponding Author:**	Vladimir Kuzmanovski, Ph.D.<br>Institut Jozef Stefan<br>Ljubljana, SLOVENIA
**Corresponding Author Secondary Information:**	
**Corresponding Author's Institution:**	Institut Jozef Stefan
**Corresponding Author's Secondary Institution:**	
**First Author:**	Vladimir Kuzmanovski, Ph.D.
**First Author Secondary Information:**	
**Order of Authors:**	Vladimir Kuzmanovski, Ph.D.<br>Ljupčo Todorovski<br>Sašo Džeroski
**Order of Authors Secondary Information:**	

Powered by Editorial Manager® and ProduXion Manager® from Aries Systems Corporation

**Response to Reviewers:**	Reply to Reviewers for Manuscript #GIGA-D-17-00222<br>Vladimir Kuzmanovski<br>Ljupčo Todorovski<br>Sašo Džeroski<br><br>1 Overview of the Manuscript Revision<br>We would like to thank the referees for their detailed and very constructive comments. Before<br>going into detailed replies to the individual requests for revisions, we first introduce the two major<br>changes we have made to the manuscript that correspond to the referee suggestions for major<br>revisions. Additionally, please find enclosed a version of the revised manuscript which contains<br>marked up changes.<br><br>1.1 Relevance Networks and Causal Relevance Networks<br>As our first major change following the comments from the first reviewer, we have repositioned the<br>relevance network (RN) approach within the taxonomy of methods for inference of gene regulatory<br>networks. Instead of distinguishing between lazy and model-based methods, as in the initial version<br>of the manuscript, the revised manuscript distinguishes between the two prevailing perspectives of<br>statistical inference and mathematical modeling. While the methods from the later paradigm deal<br>with reverse engineering of the observed biological interaction, the focus of the paper is statistical<br>inference and not reverse engineering, as rightly pointed out by the first reviewer.<br><br>Our second major revision is related to the use of the term ”relevance network”. Initially, the<br>term was introduced to denote association networks, where the direction of the links is unknown.<br>Later on, however, many authors, including (Hempel et al. 2011) have extended the scope and use<br>of the same term ”reference network” to include a class of causal networks with directed links or<br>influences between genes and gene products. To avoid misunderstandings, we follow the suggestion<br>from the first reviewer to use the term ”relevance network” in its strict sense, and introduce the<br>term ”causal relevance networks” to refer to the directed networks. Therefore, we refer to the<br>generalized approach as ”causal relevance network approach” or ”CRN approach” throughout the<br>manuscript. In the title, we use the term ”generalized relevance network approach”.<br><br>1.2 New Experiments with Steady-State Data<br>To address the comments for both reviewers on steady-state data, we decided to perform a new<br>set of experiments on tasks of gene regulatory networks inference from steady-state data. We per-<br>formed the experiments with the same experimental setup used for time-series data and included<br>the results as well as the discussion thereof in a new subsection entitled ”Steady-state data”, of<br>the section ”Results”. We have also extended the conclusions of the manuscript to incorporate<br>the summary of the results obtained on the steady-set data.<br><br>2 Detailed Replies to Individual Comments

Powered by Editorial Manager® and ProduXion Manager® from Aries Systems Corporation

2.1 Reviewer #1

Overall, a very interesting study using an interesting approach. However, there are some errors
and questions that need to be addressed in order to make the paper publishable.

1 The authors talk about 'relevance networks', and this is even in the title mentioned. However, all
methods discussed (ARACNE, CLR, MRMR etc) aim at inferring causal networks NOT relevance
networks! Relevance networks are association networks.
This needs to be corrected throughout the paper, including the title, and a discussion distin-
guishing association networks from causal networks needs to be added. This is a difficult topic
and many people make this mistake. Detailed information can be found here:
https://www.ncbi.nlm.nih.gov/pubmed/25364745
https://www.ncbi.nlm.nih.gov/pubmed/25221572
https://www.ncbi.nlm.nih.gov/pubmed/21639592

Reply: We have changed the title of the article, revised the abstract, and rewritten the introduc-
tion. We have also revised the initial part of Section "Relevance network approach" (the new title
in the revised manuscript is "The causal relevance network approach") to make a clear distinction
between "relevance networks" and "causal relevance networks". See the explanation in Section 1.1
of this document.

2 abstract: It is a general algorithm that allows the use of an arbitrary measure of pairwise asso-
ciation between nodes, an arbitrary scoring scheme that transforms the associations into weights
of the network links, and a method for inferring the directions of the network links. First this
sentence is confusing. Second, all methods discussed in this paper (including the above mentioned
one) have been introduced by NOT inferring the direction between links. Hence this is only
optional.

Reply: This sentence has been corrected in the revised manuscript, in line with the explanation
from Section 1.1 of this document.

3 Inference of GRNs is a reverse engineering task
This is not correct, it is a statistical inference task. Remove citation [1] and use instead your
citation [4].

Reply: We have removed the problematic sentence, and reordered the references and citations,
so that citation [4] has now became citation [1].

4 the most general one, groups the approaches to network inference in the two broad categories
of model-based and lazy (unsupervised) methods, where the group of model-based approaches is
further split into supervised and semi-supervised methods [4, 1]. This is not correct. The most
general way to distinguish methods is separating association networks from causal networks. For
this reason, you are dealing in your paper with causal networks.

Powered by Editorial Manager® and ProduXion Manager® from Aries Systems Corporation

Reply: As already explained in Section 1.1 of this document, we have abandoned the discussion
on lazy and model-based approaches alltogether and rather distinguish between statistical and
mathematical modeling perspective.

5 Our paper focus on the relevance-network approach [8], a lazy approach to network inference
that predicts network link
Based on 4 this needs to be corrected.

Reply: Corrected.

6 The early variants of the RN approach Rays et al. [10], Eisen et al. [11] The paper in [11] does
not consider networks at all. Remove it. The literature about RN starts with Butte et al.

Reply: We have revised the initial paragraphs of the Section ”Relevance network approach”; the
revision takes into account this comment and rephrases the comments around the above-mentioned
citations.

7 The paper aims at analyzing time series data. However, none of the methods (ARACNE, CLR,
MRMR etc) have been introduced for analyzing such data. Instead, the methods have been
introduced to study condition specific data. Discuss this in detail and justify why the methods
can be used for time series data.

Reply: To this end, we refer to the article by Hempel et al. (2011) that provides empirical evidence
that the scoring methods mentioned in the comment are capable of handling time-series data. To
further clarify the issue of handling different types of data with the generalized relevance network
approach, we have also included a whole new set of experiments on inferring causal relevance
networks from steady-state data.

8 The comparative has been conducted using simulated and real The comparison has been The
paper contains many errors like this. They all need to be correct. I suggest to consult a native
speaker.

Reply: We have carefully reread the article and corrected many typos and linguistic inconsisten-
cies.

9 In the results section, I cannot find a listing of all 114 methods studied.

Reply: We have added an Appendix with a tables listing and explaining all the 114 variants of
the generalized relevance network approach considered in the comparative analysis.

10 The axis of the figures in fig 2, 3 and 4 are not labeled. what is shown?

Reply: We have added missing axes labels and added explanations in the figure captions and the
manuscript paragraphs that interpret the figures.

Powered by Editorial Manager® and ProduXion Manager® from Aries Systems Corporation

	11 From the discussion, it is unclear to me which method was best, which was second etc. This<br>is rather cryptic. It needs to be much more clear. A problem is the 'coding' introduced by the<br>authors. You need to convert it back because otherwise it is laborsome to understand what you<br>mean.<br><br>Reply: We have improved the presentation of the results, as well as revised graphs annotations<br>and figure captions in order to clarify the mentioned issues and further clarify the meaning of the<br>obtained results.<br><br>2.2 Reviewer #2<br><br>This paper performs an extensive empirical analysis of the performance of several variants of the<br>of gene network inference from time-series data. The paper is well written and easy to read. The<br>main limitation of the paper is that it just focuses on time-series. Although this is an important<br>problem, the lack of real big and reliable datasets on which an adequate assessment could be done<br>is a big limitation of the study and the significance of the results. Moreover they lack the use of<br>other important datasets such as the IRMA network (Cantone et al, Cell 2009). Therefore, the<br>overall benchmarking is adequate but the take home message is very limited.<br><br>Reply: The revised manuscript includes a report on a whole new set of experiments on inferring<br>causal relevance networks from steady-state data. Please see Section 1.2 in the overview at the<br>beginning of this document. The new experiments include also the task of reconstruction the<br>IRMA network.<br><br>We strongly believe that the addition of the new experimental results lifts the limitation of<br>the initial version of the manuscript mentioned in the comment. Furthermore, it improves the<br>generality and potential impact of the reported results. Finally, it strengthen the overall take-home<br>message of the manuscript.<br><br>I would suggest to improve their work extending the analysis steady state networks where they<br>can find much more data before trying to publish this research.<br><br>Reply: See the reply above.
**Additional Information:**	
**Question**	**Response**
Are you submitting this manuscript to a special series or article collection?	No
**Experimental design and statistics**<br><br>Full details of the experimental design and statistical methods used should be given in the Methods section, as detailed in our Minimum Standards Reporting Checklist.	Yes

Powered by Editorial Manager® and ProduXion Manager® from Aries Systems Corporation

Information essential to interpreting the data presented should be made available in the figure legends.<br><br>Have you included all the information requested in your manuscript?	
**Resources**<br><br>A description of all resources used, including antibodies, cell lines, animals and software tools, with enough information to allow them to be uniquely identified, should be included in the Methods section. Authors are strongly encouraged to cite Research Resource Identifiers (RRIDs) for antibodies, model organisms and tools, where possible.<br><br>Have you included the information requested as detailed in our Minimum Standards Reporting Checklist?	Yes
**Availability of data and materials**<br><br>All datasets and code on which the conclusions of the paper rely must be either included in your submission or deposited in publicly available repositories (where available and ethically appropriate), referencing such data using a unique identifier in the references and in the “Availability of Data and Materials” section of your manuscript.<br><br>Have you have met the above requirement as detailed in our Minimum Standards Reporting Checklist?	Yes
