# Supplementary material for: Extensive evaluation of the generalized relevance network approach to inferring gene regulatory networks
Source: Gigascience. 2018 Sep 18;7(11):giy118. doi: 10.1093/gigascience/giy118 (PMC6420648; doi:10.1093/gigascience/giy118)
Supplement: Authors_Response_To_Reviewer_Comments_Revision_1.pdf [file giy118_authors_response_to_reviewer_comments_revision_1.pdf]

Author's Response To Reviewer Comments

Close

Dear Editor,

As requested in the last minor revision, corresponding changes have been applied.
Following is the list of corrections in the order as given in the Decision Letter:
- Numbered citation of our GigaDB repository is added in the "Availability of Data and Materials" section, with the citation provided directly from the GigaDB repository.
- The software tool we developed for the purpose of the study that can be re-used later (tool that have implementation of all variants of the approach given in the manuscript) is registered in SciCrunch.org and corresponding RRID is provided in the manuscript (under "Availability of Data and Materials" section)
- The manuscript is reorganized to comply with the journal style, as requested. "Availability of Data and Materials" is moved towards the end, after the "Conclusion" section; "Funding" section has been introduced where fundings have been provided (previously they were under acknowledgment), "Authors' contribution" section has been added, and the Abstract has been corrected to comply with the requested structure (divided in three headings: "Background","Results","Conclusions") and limit of 250 words.

With the hope that the provided changes satisfy the requested minor revision, we are looking forward for a response from the production department.

Kind regards,
Vladimir Kuzmanovski

Close
