# Supplementary material for: Extensive evaluation of the generalized relevance network approach to inferring gene regulatory networks
Source: Gigascience. 2018 Sep 18;7(11):giy118. doi: 10.1093/gigascience/giy118 (PMC6420648; doi:10.1093/gigascience/giy118)
Supplement: Reviewer_1_Report_(Original_Submission) -- Frank Emmert-Streib — 6/10/2017 Reviewed [file giy118_reviewer_1_report_(original_submission).pdf]

Reviewer Report

Title: Extensive evaluation of the generalized relevance network approach to inferring gene regulatory networks

Version: Original Submission **Date:** 10/6/2017

Reviewer name: Frank Emmert-Streib

Reviewer Comments to Author:

Overall, a very interesting study using an interesting approach. However, there are some errors and questions that need to be addressed in order to make the paper publishable.

1 The authors talk about 'reference networks', and this is even in the title mentioned. However, all methods discussed (ARACNE, CLR, MRMR etc) aim at inferring causal networks NOT reference networks! Reference networks are association networks.
This needs to be corrected throught the paper, including the title, and a discussion distinguishing association networks from causal networks needs to be added. This is a difficult topic and many people make this mistake. Detailed information can be found here:
https://www.ncbi.nlm.nih.gov/pubmed/25364745
https://www.ncbi.nlm.nih.gov/pubmed/25221572
https://www.ncbi.nlm.nih.gov/pubmed/21639592

2 abstract:
It is a general algorithm that allows the use of an arbitrary measure of pairwise association between nodes, an arbitrary scoring scheme that transforms the associations into weights of the network links, and a method for inferring the directions of the network links.
First this sentence is confusing. Second, all methods discussed in this paper (including the above mentioned one) have been introduced by NOT inferring the direction between links. Hence this is only optional.

. 3 Inference of GRNs is a reverse engineering task
This is not correct, it is a statistical inference task. Remove citation [1] and use instead your citation [4].

4 the most general one, groups the approaches to network inference in the two broad categories of model-based and lazy (unsupervised) methods, where the group of model-based approaches is further split into super- vised and semi-supervised methods [4, 1].
This is not correct. The most general way to distinguish methods is separating association networks from causal networks. For this reason, you are dealing in your paper with causal networks.
. 5 Our paper focus on the relevance-network approach [8], a lazy approach to network inference that predicts network link
Based on 4 this needs to be corrected.
6 The early variants of the RN approach Rays et al. [10], Eisen et al. [11]
The paper in [11] does not consider networks at all. Remove it. The literature about RN starts with Butte et al.

7 The paper aims at analyzing time series data. However, none of the methods (ARACNE, CLR, MRMR etc) have been introduced for analyzing such data. Instead, the methods have been introduced to study condition specific data. Discuss this in detail and justify why the methods can be used for time series data.

8 The comparative has been conducted using simulated and real
The comparison has been…
The paper contains many errors like this. They all need to be correct. I suggest to consult a native speaker.

9 In the results section, I cannot find a listing of all 114 methods studied.

10 The axis of the figures in fig 2, 3 and 4 are not labeled. what is shown?

11 From the discussion, it is unclear to me which method was best, which was second etc. This is rather cryptic. It need to be much more clear. A problem is the 'coding' introduced by the authors. You need to convert it back because otherwise it is labersome to understand what you mean.

Methods

Are the methods appropriate to the aims of the study, are they well described, and are necessary controls included? No

Conclusions

Are the conclusions adequately supported by the data shown? No

Reporting Standards

Does the manuscript adhere to the journal's guidelines on minimum standards of reporting? Yes

Choose an item.

Statistics

Are you able to assess all statistics in the manuscript, including the appropriateness of statistical tests used? There are no statistics in the manuscript.

Quality of Written English

Please indicate the quality of language in the manuscript: Needs some language corrections before being published

Declaration of Competing Interests

Please complete a declaration of competing interests, considering the following questions:

- Have you in the past five years received reimbursements, fees, funding, or salary from an organisation that may in any way gain or lose financially from the publication of this manuscript, either now or in the future?
- Do you hold any stocks or shares in an organisation that may in any way gain or lose financially from the publication of this manuscript, either now or in the future?
- Do you hold or are you currently applying for any patents relating to the content of the manuscript?
- Have you received reimbursements, fees, funding, or salary from an organization that holds or has applied for patents relating to the content of the manuscript?

- Do you have any other financial competing interests?
- Do you have any non-financial competing interests in relation to this paper?

If you can answer no to all of the above, write 'I declare that I have no competing interests' below. If your reply is yes to any, please give details below.

1 no 2 no 3 no 4 no 5 no 6 no

I agree to the open peer review policy of the journal. I understand that my name will be included on my report to the authors and, if the manuscript is accepted for publication, my named report including any attachments I upload will be posted on the website along with the authors' responses. I agree for my report to be made available under an Open Access Creative Commons CC-BY license (http://creativecommons.org/licenses/by/4.0/). I understand that any comments which I do not wish to be included in my named report can be included as confidential comments to the editors, which will not be published.

I agree to the open peer review policy of the journal

To further support our reviewers, we have joined with Publons, where you can gain additional credit to further highlight your hard work (see: https://publons.com/journal/530/gigascience). On publication of this paper, your review will be automatically added to Publons, you can then choose whether or not to claim your Publons credit. I understand this statement.

Yes Choose an item.
